# The 2014–2015 Ebola virus disease outbreak and primary healthcare delivery in Liberia: Time-series analyses for 2010–2016

**DOI:** 10.1371/journal.pmed.1002508

**Published:** 2018-02-20

**Authors:** Bradley H. Wagenaar, Orvalho Augusto, Jason Beste, Stephen J. Toomay, Eugene Wickett, Nelson Dunbar, Luke Bawo, Chea Sanford Wesseh

**Affiliations:** 1 Department of Global Health, University of Washington, Seattle, Washington, United States of America; 2 Partners in Health, Monrovia, Liberia; 3 Health Alliance International, Seattle, Washington, United States of America; 4 Universidade Eduardo Mondlane, Maputo, Mozambique; 5 Brigham and Women’s Hospital, Boston, Massachusetts, United States of America; 6 Harvard Medical School, Boston, Massachusetts, United States of America; 7 Ministry of Health, Monrovia, Liberia; Harvard University, UNITED STATES

## Abstract

**Background:**

The aim of this study is to estimate the immediate and lasting effects of the 2014–2015 Ebola virus disease (EVD) outbreak on public-sector primary healthcare delivery in Liberia using 7 years of comprehensive routine health information system data.

**Methods and findings:**

We analyzed 10 key primary healthcare indicators before, during, and after the EVD outbreak using 31,836 facility-month service outputs from 1 January 2010 to 31 December 2016 across a census of 379 public-sector health facilities in Liberia (excluding Montserrado County). All indicators had statistically significant decreases during the first 4 months of the EVD outbreak, with all indicators having their lowest raw mean outputs in August 2014. Decreases in outputs comparing the end of the initial EVD period (September 2014) to May 2014 (pre-EVD) ranged in magnitude from a 67.3% decrease in measles vaccinations (95% CI: −77.9%, −56.8%, *p <* 0.001) and a 61.4% decrease in artemisinin-based combination therapy (ACT) treatments for malaria (95% CI: −69.0%, −53.8%, *p <* 0.001) to a 35.2% decrease in first antenatal care (ANC) visits (95% CI: −45.8%, −24.7%, *p <* 0.001) and a 38.5% decrease in medroxyprogesterone acetate doses (95% CI: −47.6%, −29.5%, *p <* 0.001). Following the nadir of system outputs in August 2014, all indicators showed statistically significant increases from October 2014 to December 2014. All indicators had significant positive trends during the post-EVD period, with every system output exceeding pre-Ebola forecasted trends for 3 consecutive months by November 2016. Health system outputs lost during and after the EVD outbreak were large and sustained for most indicators. Prior to exceeding pre-EVD forecasted trends for 3 months, we estimate statistically significant cumulative losses of −776,110 clinic visits (95% CI: −1,480,896, −101,357, *p =* 0.030); −24,449 bacille Calmette–Guérin vaccinations (95% CI: −45,947, −2,020, *p =* 0.032); −9,129 measles vaccinations (95% CI: −12,312, −5,659, *p <* 0.001); −17,191 postnatal care (PNC) visits within 6 weeks of birth (95% CI: −28,344, −5,775, *p =* 0.002); and −101,857 ACT malaria treatments (95% CI: −205,839, −2,139, *p =* 0.044) due to the EVD outbreak. Other outputs showed statistically significant cumulative losses only through December 2014, including losses of −12,941 first pentavalent vaccinations (95% CI: −20,309, −5,527, *p =* 0.002); −5,122 institutional births (95% CI: −8,767, −1,234, *p =* 0.003); and −45,024 acute respiratory infections treated (95% CI: −66,185, −24,019, *p <* 0.001). Compared to pre-EVD forecasted trends, medroxyprogesterone acetate doses and first ANC visits did not show statistically significant net losses. ACT treatment for malaria was the only indicator with an estimated net increase in system outputs through December 2016, showing an excess of +78,583 outputs (95% CI: −309,417, +450,661, *p =* 0.634) compared to pre-EVD forecasted trends, although this increase was not statistically significant. However, comparing December 2013 to December 2017, ACT malaria cases have increased 49.2% (95% CI: 33.9%, 64.5%, *p <* 0.001). Compared to pre-EVD forecasted trends, there remains a statistically significant loss of −15,144 PNC visits within 6 weeks (95% CI: −29,453, −787, *p =* 0.040) through December 2016.

**Conclusions:**

The Liberian public-sector primary healthcare system has made strides towards recovery from the 2014–2015 EVD outbreak. All primary healthcare indicators tracked have recovered to pre-EVD levels as of November 2016. Yet, for most indicators, it took more than 1 year to recover to pre-EVD levels. During this time, large losses of essential primary healthcare services occurred compared to what would have been expected had the EVD outbreak not occurred. The disruption of malaria case management during the EVD outbreak may have resulted in increased malaria cases. Large and sustained investments in public-sector primary care health system strengthening are urgently needed for EVD-affected countries.

## Abstract 2

Bradley Wagenaar and colleagues use health facility data to analyze losses to essential primary healthcare service outputs during and immediately after the EVD outbreak, and their recovery in the years after.

## Author summary

**Why was this study done?:**

- Almost 2 years after the final case of Ebola virus disease (EVD) was discharged in Liberia, it is essential to understand the lingering effects of the EVD outbreak on the public-sector primary healthcare system.
- Existing studies of the effects of the EVD outbreak on population health and health system functioning have relied on small survey samples, mathematical modeling approaches, and data collected at single points in time before, during, or immediately after the epidemic rather than using health system output data to understand long-term system trends.
- We aimed to improve on these limitations and extend existing analyses to cover a census of health facilities in Liberia (excluding Montserrado County) from January 2010 to December 2016.

**What did the authors do and find?:**

- We used health facility data across a census of facilities and over an 84-month period to analyze changes in essential primary healthcare service indicators during and after the EVD outbreak.
- We found that it took only 4 months (June–September 2014) to lose between 35% and 67% of essential primary care health system outputs across Liberian clinics, and that, as of November 2016 (19 months post-EVD), all health system indicators had recovered to their pre-EVD levels.
- Given that health system outputs are time-limited, the loss of an estimated 776,110 clinic visits, 24,449 bacille Calmette–Guérin vaccinations, 9,129 measles vaccinations, 12,941 first pentavalent vaccinations, 5,122 institutional births, 17,191 postnatal care visits within 6 weeks of birth, 101,857 artemisinin-based combination therapy treatments for malaria, and 45,024 treatments of acute respiratory infections due to the EVD outbreak will continue to severely affect population health.
- We estimate that the number of malaria cases treated has increased by 49% from December 2013 to December 2016—a finding potentially due to increases in the transmission of the disease following large-scale treatment interruptions during the EVD outbreak.

**What do these findings mean?:**

- These findings add to the body of literature suggesting that the 2014–2015 EVD outbreak across West Africa had devastating effects on the delivery of all types of public-sector primary healthcare services.
- For most primary healthcare indicators, it took more than 1 year post-EVD to recover to pre-EVD levels, with net losses persisting as of December 2016.
- Malaria cases may have increased since the EVD outbreak in Liberia, as predicted from previous modelling efforts across EVD-affected countries; the extent to which a similar trend for malaria has been recorded in Sierra Leone or Guinea is not clear.
- Public-sector primary healthcare system strengthening is urgently needed across West Africa, with specific focus targeted to general outpatient care, childhood vaccination, maternal health, and malaria prevention/treatment.

## Introduction

The 2014–2015 Ebola virus disease (EVD) outbreak across West Africa represented an international tragedy, directly leading to 28,616 cases of EVD and 11,310 deaths in total, and 10,675 confirmed, probable, and suspected cases in Liberia, resulting in 4,809 deaths [1]. Due to already weakened health systems, the EVD outbreak led to incredible disruption in the continued provision of life-saving public-sector primary healthcare across Sierra Leone, Guinea, and Liberia—the 3 countries most severely impacted by the epidemic [2]. Analyses using routine health information system (RHIS) data originating from Guinea and Sierra Leone have recently chronicled the effect of the EVD epidemic on the delivery of public-sector care for maternal, child, and reproductive health services, showing dramatic decreases during the Ebola outbreak (on the order of 50% declines) and sustained low levels not suggesting recovery [3–5]. Previous descriptive studies using RHIS data from Liberia have shown decreases in maternal and child health (MCH) indicators [6,7], malaria treatment [8], HIV testing [9], and tuberculosis diagnoses [10] during the EVD outbreak. Others have estimated that EVD-related disruptions in treating malaria alone will contribute to significantly more excess deaths than direct EVD-related mortality [11]. Mathematical modeling of malaria in Liberia has estimated that the disruption of treatment due to EVD will result in 520,000 untreated malaria cases, 57,200 new malaria cases that would not have occurred otherwise, and a 62% increase in malaria-attributable mortality [12]. Other modeling approaches have suggested that EVD-related deaths may significantly decrease life expectancy across Sierra Leone, Liberia, and Guinea [13]—not to mention collateral morbidity and mortality due to the disruption of these countries’ health systems. The majority of existing studies on the effects of the EVD outbreak on the delivery of primary healthcare have used survey sampling data or mathematical modeling methods [14–30].

Now, almost 2 years after the final case of EVD was discharged in Liberia, it is essential to understand the lingering effects of the EVD outbreak on the public-sector health system. Tragically, one of the most enduring legacies of this global health emergency was the death of health workers. Liberia lost 184 doctors, nurses, and midwives due to EVD—a loss of 8% of the nationwide total—leaving Liberia with only 2.1 doctors, nurses, and midwives per 10,000 population; this density ranks as one of the lowest in the world and is far below the minimum World Health Organization standard of 23 per 10,000 [31]. While there have been a significant number of studies examining the effect of the EVD outbreak on health systems, including a number from Liberia, the majority have had significant limitations, including the following: (1) analyzing a small sub-sample of health facilities in select districts; (2) using only 1 year of data to populate pre-EVD and/or post-EVD trends; (3) analyzing data at an aggregate district, provincial, or national level rather than at the facility level; (4) using strict linear time trends rather than more flexible functional forms; (5) using statistical methods that fail to account for data clustering, seasonality, or autocorrelation; (6) a lack of forecasting to examine the impact of the EVD outbreak versus a counterfactual; and (7) focusing on only 1 small subset of primary healthcare indicators in a given analysis.

The aim of the present study is to extend previous research estimating the effects of the EVD outbreak on health system outputs by analyzing monthly facility-level system outputs for 7 years across 10 key primary healthcare indicators in a census of public-sector health facilities in Liberia, excluding Montserrado County. We additionally aim to use more than 4 years of pre-EVD data to generate accurate forecasts, allowing estimation of lost system outputs due to the EVD outbreak in Liberia to aid in future targeting of health system strengthening.

## Methods

### Ethics statement

This paper was approved by the Liberian Ministry of Health (MoH) and used existing RHIS data that do not qualify as human subjects research.

### Data sources and outcomes

The primary outcome was monthly facility-level time-series data abstracted from the Liberian MoH RHIS, which is integrated with District Health Information Software 2 (DHIS 2) open-source software. We abstracted all available data from 1 January 2010 to 31 December 2016 across indicators and health facilities nationwide, with the exception of facilities in Montserrado County, which houses the capital of Monrovia (see S1 Fig for the county structure of Liberia). We excluded health facilities in Montserrado County for 2 reasons. First, Montserrado County has unique sociodemographic characteristics compared to the rest of Liberia in that 85% of its population is in the fourth or highest wealth quintile, compared to only 14% in these quintiles averaging across all other counties nationwide [32]. Second, and more pertinent to the present analyses, there exist numerous for-profit, religious, and not-for-profit clinics and pharmacies operating in Montserrado County, which do not report to the MoH and thus would not be captured in these analyses. This contrasts to the other 14 counties nationwide, where few private clinics exist, and the vast majority of care is provided by public-sector clinics reporting through the national RHIS. Our analyses included the following indicators: (1) clinic visits; (2) bacille Calmette–Guérin (BCG) vaccinations; (3) measles vaccinations; (4) first pentavalent vaccinations; (5) first antenatal care (ANC) visits; (6) institutional births; (7) postnatal care (PNC) visits within 6 weeks of birth; (8) artemisinin-based combination therapy (ACT) treatments for malaria; (9) acute respiratory infections (ARIs) treated; and (10) medroxyprogesterone acetate doses. These indicators were selected from DHIS 2 as they represent key outputs for the effective delivery of primary healthcare across Liberia that have not changed in definition and data collection procedures during the time period of interest (2010–2016). PNC visits within 6 weeks and ARIs treated were analyzed beginning 1 January 2012 due to inconsistent reporting in the MoH system prior to this time.

### Statistical analyses and data cleaning

The specific hypotheses we intended to test were as follows: (1) Did the EVD outbreak lead to statistically significant reductions in primary healthcare outputs? (2) What is the magnitude of lost primary healthcare outputs compared to pre-EVD forecasted trends, and how do these reductions differ by primary care indicator? (3) Have primary healthcare outputs recovered to pre-EVD levels as of December 2016? (4) What is the magnitude of recovery of primary healthcare outputs, and how does this differ by primary care indicator?

Our a priori analysis plan included (1) data cleaning and outlier identification using individual facility-level local regression analyses over time; (2) initial univariate analyses of indicators over time to determine functional forms for model parameterization; (3) analyses of the effect of the EVD outbreak on each indicator; and (4) forecasts to determine estimates of lost health system outputs due to the EVD outbreak.

Initial data abstraction identified 379 public-sector MoH facilities reporting through the DHIS 2 system from 1 January 2010 to 31 December 2016 across all counties excluding Montserrado County. Individual facility-level local regression analyses were conducted, with outliers identified and set to missing if they exceeded 8 standard deviations from the mean time trend. Measles vaccination was excluded from facility-level local regression outlier analyses due to a measles campaign during the Ebola outbreak that led to informative data that would have been incorrectly tagged as outliers by outlier analysis. Facilities were excluded from analyses if they failed to report at least 12 monthly observations over the 7-year period for a given indicator. After observing large facility-level heterogeneity in intercepts, as well as trends over time, we employed mixed-effects models with random intercepts for facility and random slopes over time (see equation below).

$${Indicator}_{it}=\beta_{0i}+\left(\sum_{y=1}^{7}\beta_{iy}{Spline}_{yt}\right)+\left(\sum_{m=2}^{12}\beta_{m}Month\right)+\beta_{c}{Catchment}_{it}+\beta_{E1}Ebola1+\beta_{E2}Ebola2+\beta_{AE}AfterEbola$$

where Indicator_*it*_ represents 1 of 10 key primary healthcare system outputs included in this study (e.g., clinic visits); subscripts *i*, *y*, and *t* indicate a health facility *i* (running from 1 to 379) in calendar year *y* (running from 1 to 7) and at month *t* (running from 1 to 84); β_0*i*_ represents the model intercept with both a fixed effect and facility-level random effects; Spline_*yt*_ indexes time within calendar year *y* at month *t* (these linear splines include fixed effects and random effects by facility); Month is an individual dummy variable indexing month of the year using the month of January as the reference category; Catchment_*it*_ is the catchment population for health facility *i* at month *t*; Ebola1 counts the months since the beginning of the Ebola outbreak in June 2014; Ebola2 counts the months since October 2014; and AfterEbola counts the months since the end of the Ebola outbreak in May 2015.

Since RHIS data are facility-level counts, our initial a priori analysis plan was to use Poisson or negative binomial models with health facility catchment population as an offset term. However, the final model we employed was a linear mixed-effects model with a normal residual distribution. We used a linear mixed-effects model for 3 reasons. First, attempts to run these complex models (>25,000 observations; 8 random effects) as Poisson models failed to converge without simplifications such as the elimination of facility-level random slopes and the elimination of an AR(1) structure for autocorrelation in residual errors. Second, a linear mixed model converged and resulted in a stable model with approximately normal residual errors and best linear unbiased predictors (predicted random effects). Third, using linear models allows the intuitive interpretation of absolute differences in health facility outputs over time, rather than ratios of rates on a multiplicative scale, as one would receive from a Poisson model, which are often less easy to interpret by policymakers and implementers.

After initial univariate analyses of indicators over time, we modeled the time effect using yearly splines, and the effect of Ebola was modeled using a segmented regression parameterization with pre-EVD trends (1 January 2010–31 May 2014), trends for the first 4 months of the EVD outbreak (1 June 2014–30 September 2014), trends for the middle 3 months of the EVD outbreak (1 October 2014–31 December 2014), trends for the last 4 months of the EVD outbreak (1 January 2015–30 April 2015), and post-Ebola trends (1 May 2015–31 December 2016). Due to large observed seasonal effects, we included fixed-effect monthly indicator variables in all models. An AR(1) structure was used to account for autocorrelation in residual errors. To control for clinic-level catchment population, we included a fixed effect of facility-level catchment population, which aids in explaining some of the non-random natural heterogeneity of health facilities. Missing data were accounted for in the mixed-effects models using standard maximum likelihood estimation. In order to develop forecasts, we reran the abovementioned mixed-effects linear models to extend pre-EVD trends through December 2016 as a “counterfactual” to the observed EVD outbreak. We simulated 1,000 predictions per month under each model (full and counterfactual) using the coefficients and covariance matrix of the fixed parts of each model using a multivariate normal distribution [33]. Lost health system outputs were computed as the differences between the mean simulated values under the full model and the forecasted (counterfactual) model. To compute 95% confidence intervals around these differences, we used the range from the percentiles 2.5 and 97.5 of simulated values. *p*-Values were calculated as the smaller of the proportion of simulated values falling either above or below zero. This value was then multiplied by 2 to represent a 2-sided *p*-value. All analyses were conducted using Stata 15 and a 2-sided alpha value of 0.05 (see S1 Text for Stata do file).

## Results

Across the 379 clinics and the 31,836 clinic-months in the analyses, the mean clinic-level catchment population was 6,912 (range: 214–67,381), serving an estimated total population of approximately 2.6 million (Table 1). Across the 14 counties included in the analyses, there were a total of 2,320 EVD cases, ranging from 2 cases in Grand Gedeh and River Cess to 746 cases in Lofa. Data from previous community surveys suggest that all counties except Sinoe and Grand Kru have more than 90% of women attending at least 1 ANC visit, although on average across all counties, only 52.7% of women deliver in a health facility (range: 34.6%–75.6%). The average BCG coverage across counties is 89.5%, with measles coverage lower, at 70.6%. Malaria prevalence in children is high, at 34.4%.

**Table 1:** Background information on a census of 379 public-sector health facilities reporting through DHIS 2 by county and by indicator across all Liberian counties excluding Montserrado, 2010–2016.

County	Total number of facilities (%)^†^	Mean facility catchment population (SD)^†^	Total catchment population served	Total confirmed EVD cases^‡^	Percent of population in lowest wealth quintile^‖^	Percent of women aged 15–49 years receiving prenatal care from skilled provider^‖^	Percent of live births delivered in health facility^‖^	BCG vaccine coverage^‖^	Measles vaccine coverage^‖^	Malaria prevalence in children aged 6–59 years using microscopy^¶^
All counties*	379 (100)	6,912 (6,942)	2,619,648	2,320^‡^	35.6	93.5	52.7	89.5	70.6	34.4
Bomi	22 (5.8)	4,246 (4,978)	93,412	68	27.8	91.3	64.1	98.4	85.3	29.0
Bong	33 (8.7)	11,221 (11,716)	370,293	163	34.1	95.3	34.6	88.9	68.6	35.0
Gbarpolu	14 (3.7)	6,593 (5,328)	92,302	24	41.1	94.9	47.7	86.0	74.2	29.0
Grand Bassa	23 (6.1)	10,530 (7,144)	242,190	27	43.0	92.4	40.2	91.7	66.4	26.2
Grand Cape Mount	32 (8.4)	4,410 (3,023)	141,120	71	28.1	96.4	39.0	96.5	83.1	29.0
Grand Gedeh	18 (4.8)	7,727 (7,696)	139,086	2	35.7	95.3	69.2	92.4	79.0	32.6
Grand Kru	16 (4.2)	3,843 (1,607)	61,488	9	53.3	86.3	51.1	74.8	56.4	49.2
Lofa	52 (13.7)	5,912 (5,145)	307,424	746	33.3	90.8	75.6	98.9	80.0	35.0
Margibi	25 (6.6)	10,030 (10,083)	250,750	179	13.6	97.4	51.3	94.8	76.8	26.2
Maryland	20 (5.3)	7,087 (9,105)	141,740	3	21.6	92.1	54.3	83.5	62.7	49.2
Nimba	54 (14.3)	9,297 (5,307)	502,038	25	14.5	98.3	48.0	93.4	73.3	35.0
River Cess	19 (5.0)	4,729 (2,055)	89,851	2	39.3	94.8	52.7	76.7	60.8	32.6
River Gee	19 (5.0)	3,904 (2,551)	74,176	4	71.3	96.8	58.8	92.4	57.4	49.2
Sinoe	32 (8.4)	3,541 (2,857)	113,312	17	41.3	87.2	51.3	84.7	64.5	32.6

*Excluding Montserrado County; direct unweighted average of the remaining counties except facility catchment population, which is a total.

^†^Source: Liberian Ministry of Health, from online DHIS 2 system, averaged across follow-up period (2010–2016).

^‡^Source: Liberian Ministry of Health. Note: total across counties includes 980 EVD cases with unknown county origin.

^‖^Source: Liberia Demographic and Health Survey 2013.

^¶^Source: Liberia Malaria Indicator Survey 2011. Note: only regional data were collected; no county-level information was presented.

BCG, bacille Calmette–Guérin; DHIS 2, District Health Information Software 2; EVD, Ebola virus disease.

Given that not all clinics perform, and thus report, all 10 primary care indicators, the number of facilities and facility-months included in analyses vary by indicator. Values range from 379 clinics and 31,736 clinic-months for clinic visits and ACT treatments for malaria to 275 clinics and 23,100 clinic-months for institutional births and 244 clinics and 14,640 clinic-months for treatment of ARIs (see Table 2). The proportion of clinic-months missing also varied from a low of 4.8% for outpatient clinic visits to a high of 14.3% for medroxyprogesterone acetate doses. The number of outliers identified through facility-level local regression analyses ranged from 1.1% of clinic-months for BCG vaccinations and first ANC visits to a low of 0.34% for ARIs. Rates of missing data varied over time, with most indicators showing more missing data earlier in the time series (2010–2014) compared to in later years. For some indicators, there was a slight increase in missing data during the first 4 months of the EVD outbreak (June–September 2014) compared to the 5 months just prior to the EVD outbreak (January–May 2014); however, these increases were relatively small (clinic visits: 4.3% to 7.3%; institutional births: 5.1% to 6.0%; ACT treatments: 4.6% to 8.0%) and not consistent for all indicators (BCG doses: 6.4% to 6.3%; measles doses: 8.4% to 7.1%).

**Table 2:** Dates system outputs surpassed pre-Ebola forecasted trends for 3 months, total system outputs estimated to be lost due to the Ebola virus disease (EVD) outbreak (June 2014–April 2015), and number of clinics and clinic-months included for 10 key health system outputs across a census of clinics providing services in Liberia excluding Montserrado County, 2010–2016.

System output	Number of clinics and clinic-months reporting at least 12 monthly observations, 2010–2016	Number (%) of missing values across reporting clinics	Number (%) of outliers exceeding 8 SD from mean in clinic-level local regressions	Calendar date at which system outputs surpass pre-EVD forecasted trend for 3 consecutive months	Total system outputs lost due to Ebola prior to exceeding pre-Ebola trend (95% CI), *p-*value	Total system outputs lost due to Ebola through December 2014 (95% CI), *p-*value	Total system outputs lost due to Ebola through December 2016 (95% CI), *p-*value
Clinic visits	379 clinics; 31,836 clinic−months	1,550 (4.9%)	234 (0.74%)	Apr 2016	−776,110 (−1,480,896, −101,357) *p =* 0.030	−496,475 (−733,903, −260,558) *p <* 0.001	−709,610 (−1,810,253, +355,125) *p =* 0.184
BCG vaccinations	319 clinics; 26,796 clinic−months	2,926 (10.9%)	300 (1.1%)	Aug 2016	−24,449 (−45,947, −20,20) *p =* 0.032	−12,642 (−18,431, −6,550) *p <* 0.001	−21,642 (−49,043, +6,430) *p =* 0.130
Measles vaccinations	319 clinics; 26,796 clinic−months	3,058 (11.4%)	n/a*	Feb 2015	−9,129 (−12,312, −5,659) *p <* 0.001	−7,719 (−11,383, −3,706) *p <* 0.001	−3,214 (−25,352, +18,698) *p =* 0.772
First pentavalent vaccinations	319 clinics; 26,796 clinic−months	2,017 (7.5%)	246 (0.92%)	Jun 2016	−23,077 (−47,704, +1,706) *p =* 0.058	−12,941 (−20,309, −5,527) *p =* .002	−14,448 (−48,172, +19,500) *p =* 0.382
First antenatal care visits	276 clinics; 23,184 clinic−months	1,532 (6.6%)	250 (1.1%)	Nov 2016	−13,189 (−49,765, +23,320) *p =* 0.482	−8,655 (−17,380, +209) *p =* 0.060	−12,426 (−53,898, +29,546) *p =* 0.558
Institutional births	275 clinics; 23,100 clinic−months	2,890 (12.5%)	162 (0.70%)	Dec 2015	−7,243 (−15,554, +1,502) *p =* 0.124	−5,122 (−8,767, −1,234) *p =* 0.006	−1,639 (−18,343, +16,229) *p =* 0.804
Postnatal care within 6 weeks^†^	274 clinics; 16,440 clinic−months	2,022 (12.3%)	62 (0.38%)	Sep 2016	−17,191 (−28,344, −5,775) *p =* 0.002	−6,041 (−9,044, −2,922) *p <* 0.001	−15,144 (−29,453, −787) *p =* 0.040
ACT treatments for malaria	379 clinics; 31,836 clinic−months	2,012 (6.3%)	224 (0.70%)	May 2015	−101,857 (−205,839, −2,139) *p =* 0.044	−99,454 (−180,972, −20,768) *p =* 0.014	+78,583 (−309,417, +450,661) *p =* 0.634
Acute respiratory infections treated^†^	244 clinics; 14,640 clinic−months	1,025 (7.0%)	50 (0.34%)	Feb 2016	−54,549 (−111,392, −2,264) *p =* 0.058	−45,024 (−66,185, −24,019) *p <* 0.001	−38,041 (−134,775, +59,411) *p =* 0.444
Medroxyprogesterone acetate doses	272 clinics; 22,848 clinic−months	3,275 (14.3%)	186 (0.81%)	Nov 2015	−15,889 (−41,066, +9,314) *p =* 0.232	−9,098 (−20,222, +2,126) *p =* 0.124	−3,951 (−57,745, +52,667) *p =* 0.88

*Measles vaccination was excluded from facility-level local regression outlier analyses due to a measles campaign during the Ebola outbreak.

^†^Postnatal care within 6 weeks and acute respiratory infections were analyzed from January 2012 – December 2016 due to inconsistent use in the MoH system prior to January 2012.

ACT, artemisinin-based combination therapy; BCG, bacille Calmette–Guérin; n/a, not applicable; MoH, Ministry of Health.

### Pre-EVD trends (January 2010–May 2014)

**Fig 1:**
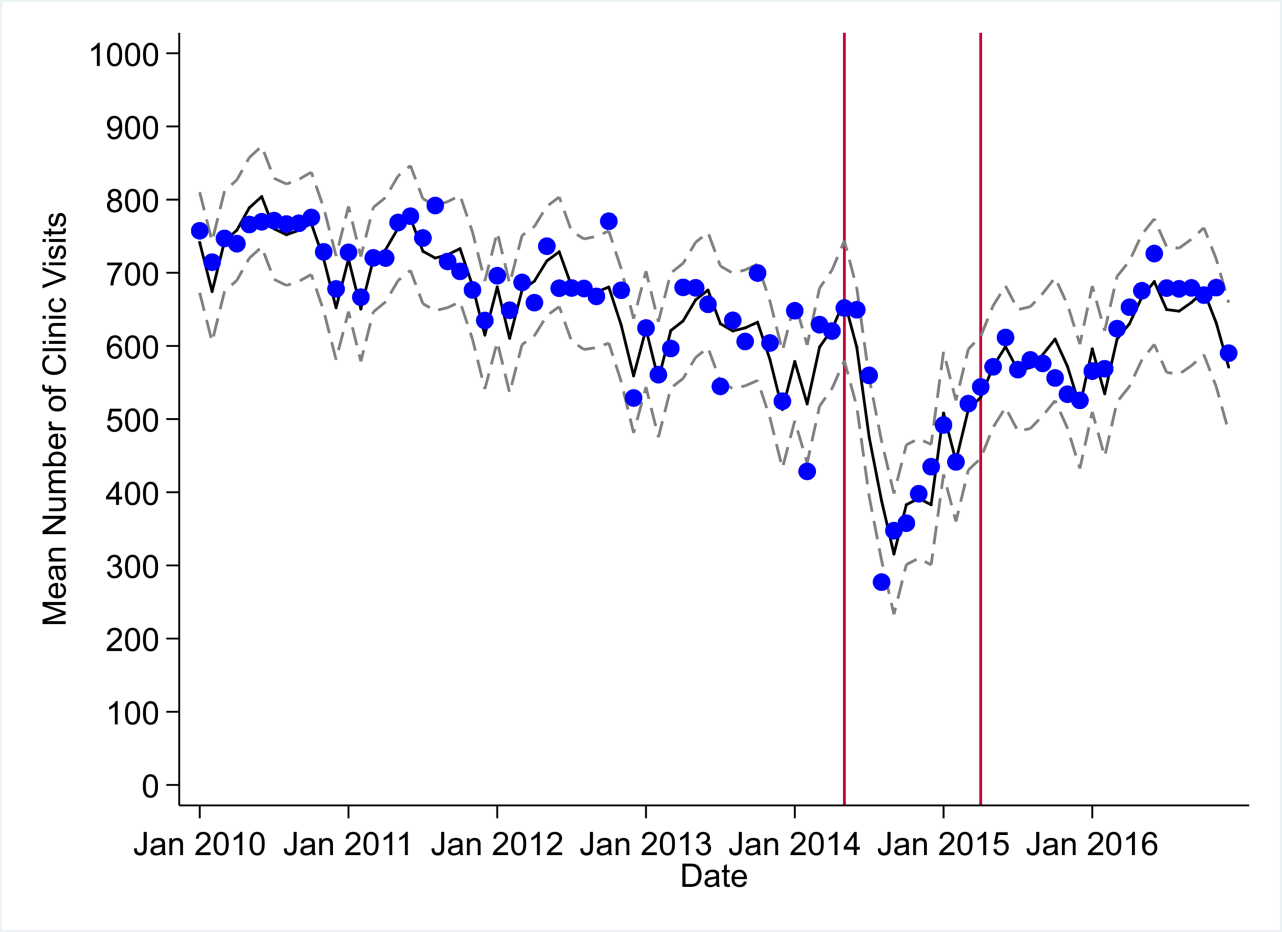
Mean trends and system losses due to Ebola virus disease (EVD) outbreak (June 2014–April 2015) for clinic visits in a census of 379 clinics providing services in Liberia from 2010–2016, excluding Montserrado County. The black solid line represents the fitted mean from a linear mixed model using a segmented regression parameterization, random intercepts and slopes by facility, monthly indicator variables to adjust for seasonality, a fixed effect to adjust for clinic-level catchment area, and an AR(1) structure to account for autocorrelation in residual errors. Gray dashed lines are 95% confidence intervals around the fitted mean. Red lines are placed at the final month before the start (May 2014) and end (April 2015) of the EVD outbreak in Liberia.

**Fig 2:**
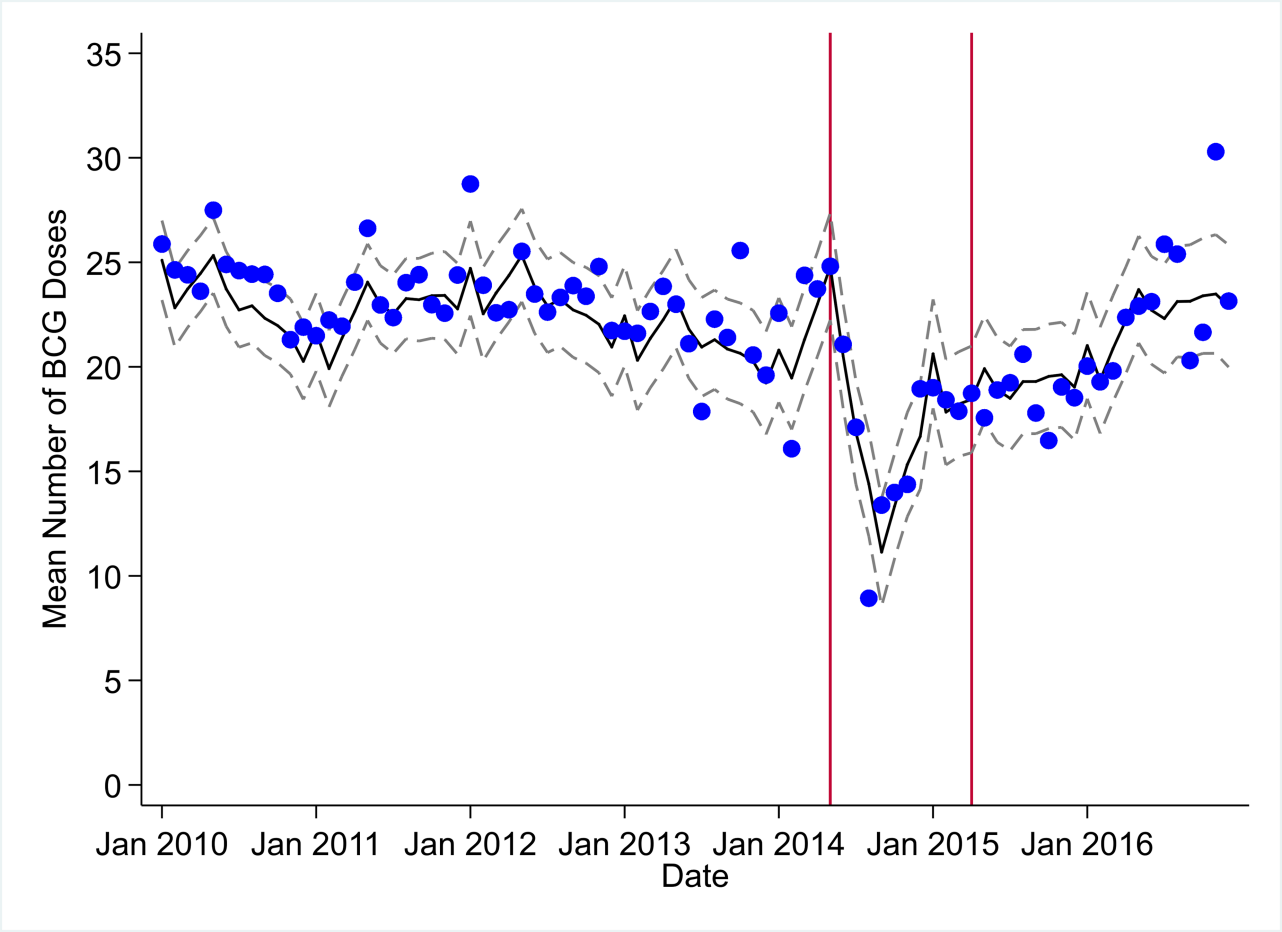
Mean trends and system losses due to Ebola virus disease (EVD) outbreak (June 2014–April 2015) for bacille Calmette–Guérin (BCG) vaccinations in a census of 379 clinics providing services in Liberia from 2010–2016, excluding Montserrado County. The black solid line represents the fitted mean from a linear mixed model using a segmented regression parameterization, random intercepts and slopes by facility, monthly indicator variables to adjust for seasonality, a fixed effect to adjust for clinic-level catchment area, and an AR(1) structure to account for autocorrelation in residual errors. Gray dashed lines are 95% confidence intervals around the fitted mean. Red lines are placed at the final month before the start (May 2014) and end (April 2015) of the EVD outbreak in Liberia.

**Fig 3:**
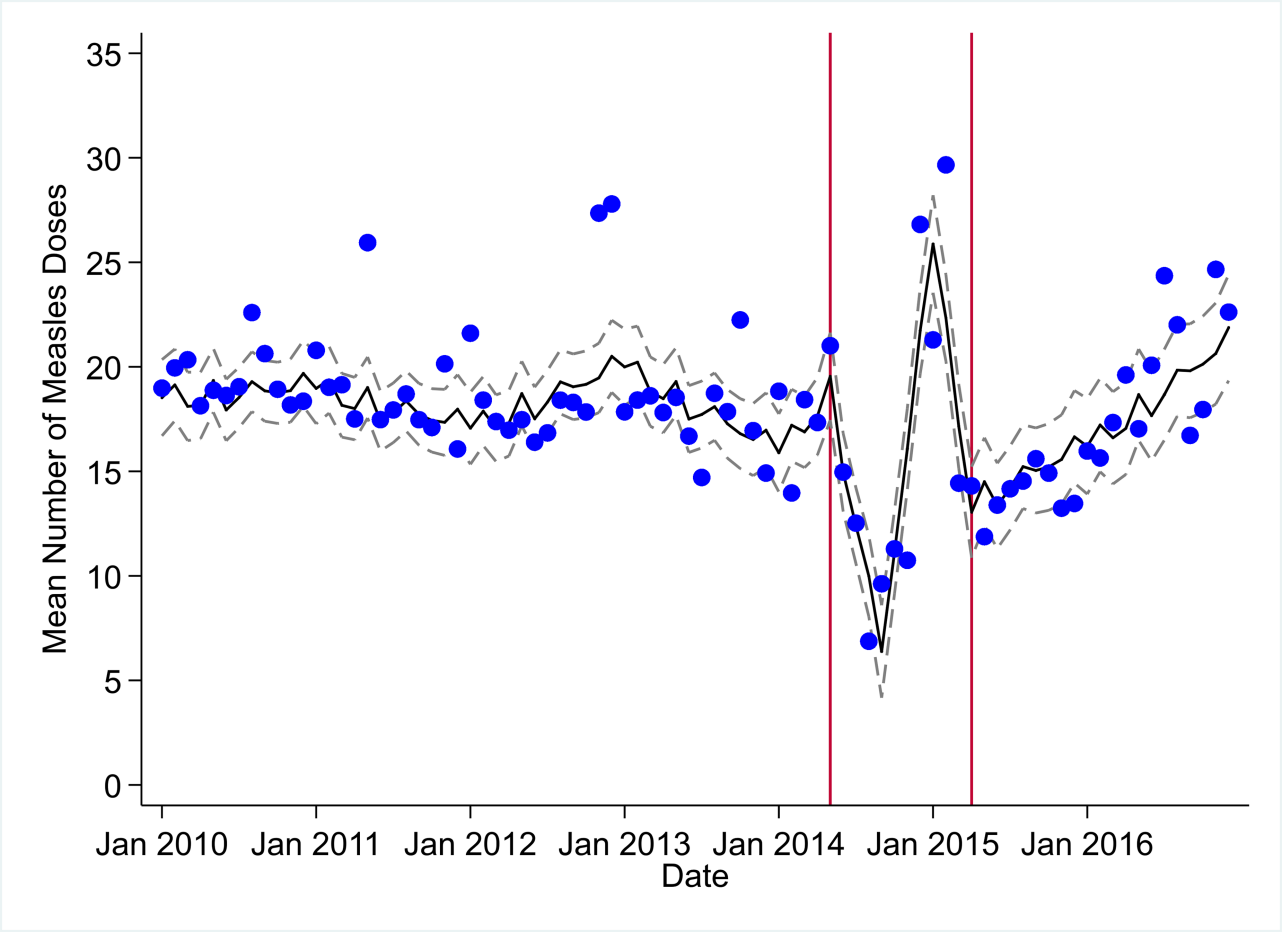
Mean trends and system losses due to Ebola virus disease (EVD) outbreak (June 2014–April 2015) for measles vaccinations in a census of 379 clinics providing services in Liberia from 2010–2016, excluding Montserrado County. The black solid line represents the fitted mean from a linear mixed model using a segmented regression parameterization, random intercepts and slopes by facility, monthly indicator variables to adjust for seasonality, a fixed effect to adjust for clinic-level catchment area, and an AR(1) structure to account for autocorrelation in residual errors. Gray dashed lines are 95% confidence intervals around the fitted mean. Red lines are placed at the final month before the start (May 2014) and end (April 2015) of the EVD outbreak in Liberia.

**Fig 4:**
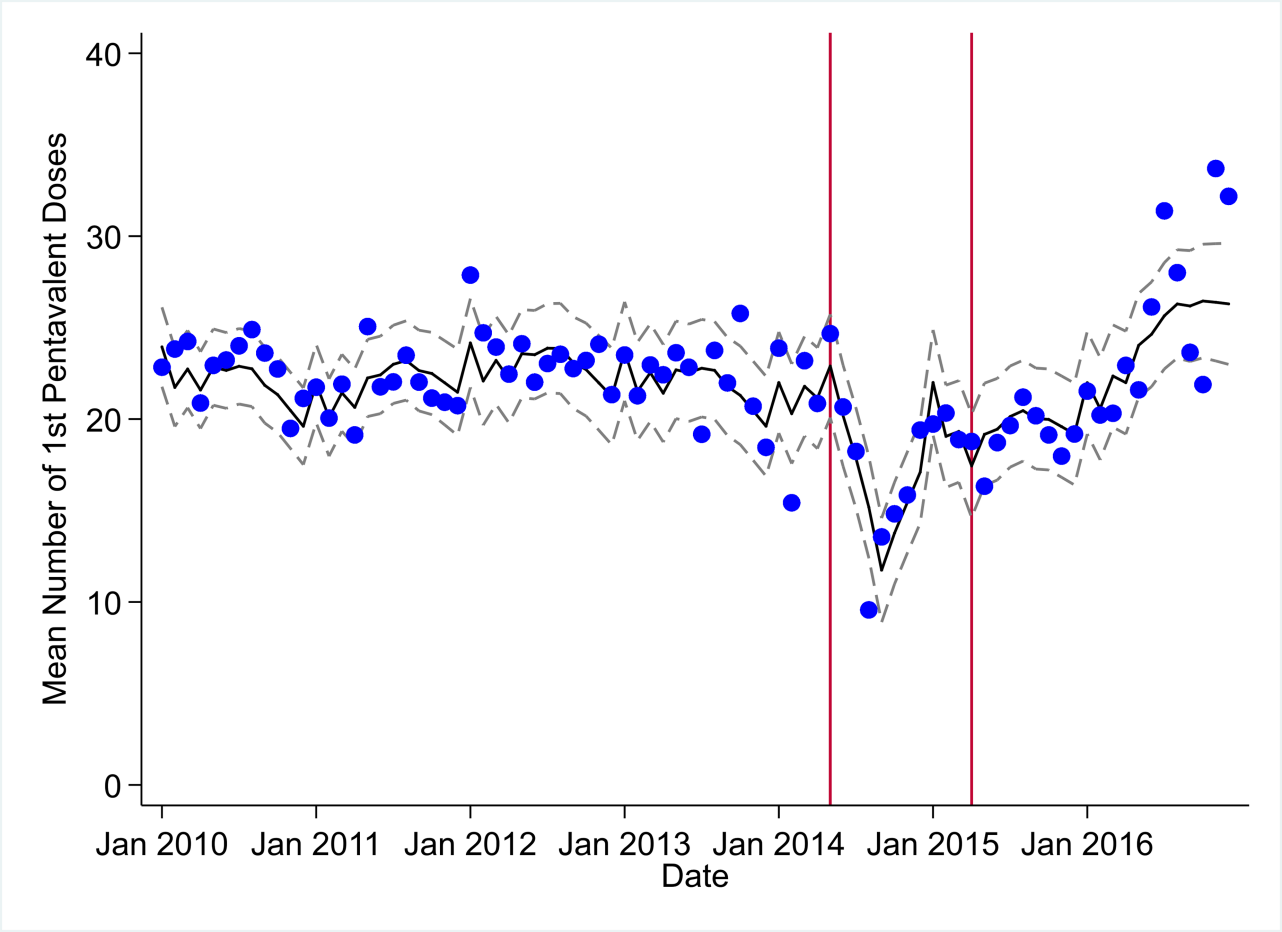
Mean trends and system losses due to Ebola virus disease (EVD) outbreak (June 2014–April 2015) for first pentavalent vaccinations in a census of 379 clinics providing services in Liberia from 2010–2016, excluding Montserrado County. The black solid line represents the fitted mean from a linear mixed model using a segmented regression parameterization, random intercepts and slopes by facility, monthly indicator variables to adjust for seasonality, a fixed effect to adjust for clinic-level catchment area, and an AR(1) structure to account for autocorrelation in residual errors. Gray dashed lines are 95% confidence intervals around the fitted mean. Red lines are placed at the final month before the start (May 2014) and end (April 2015) of the EVD outbreak in Liberia.

**Fig 5:**
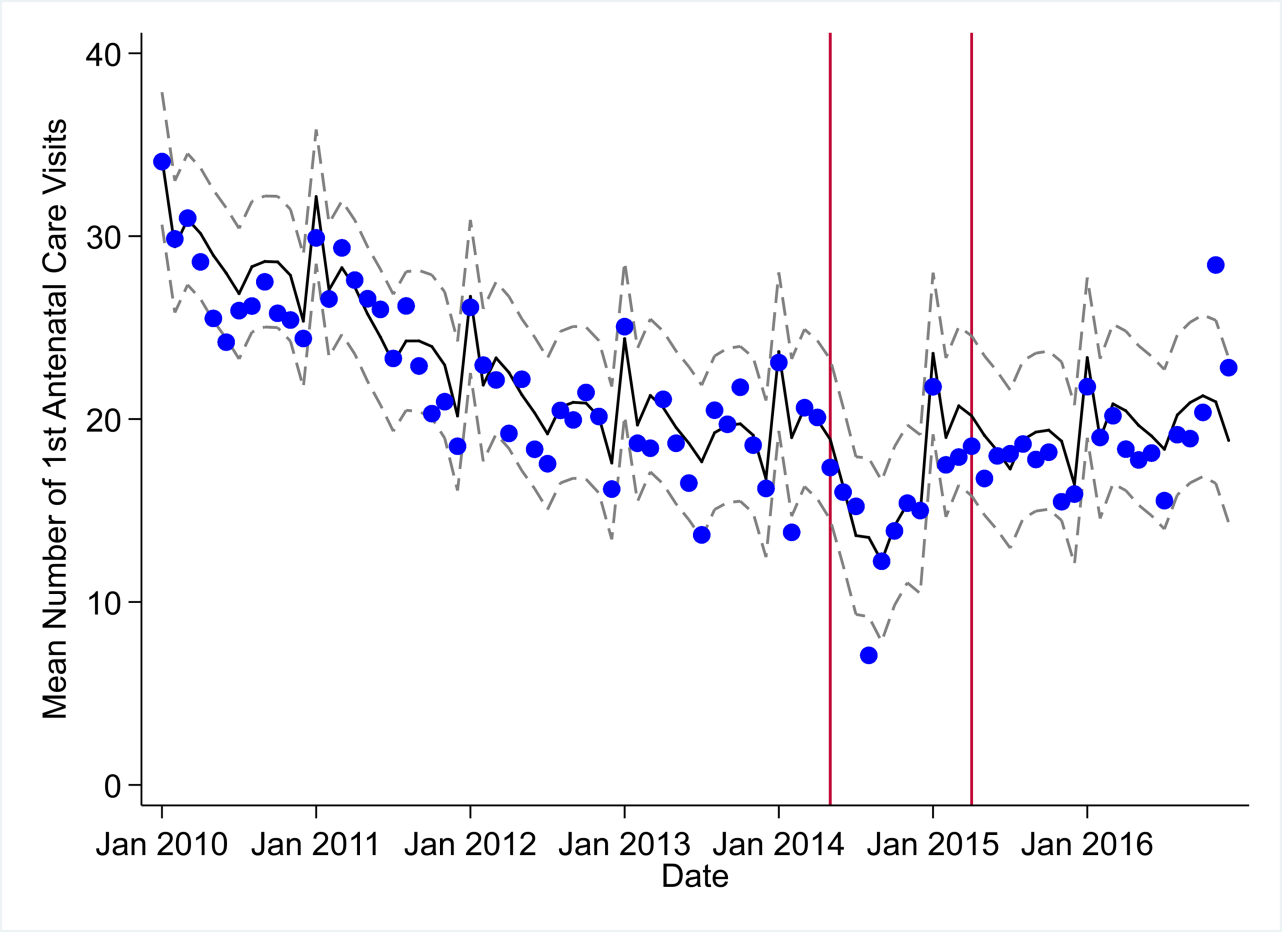
Mean trends and system losses due to Ebola virus disease (EVD) outbreak (June 2014–April 2015) for first antenatal care visits in a census of 379 clinics providing services in Liberia from 2010–2016, excluding Montserrado County. The black solid line represents the fitted mean from a linear mixed model using a segmented regression parameterization, random intercepts and slopes by facility, monthly indicator variables to adjust for seasonality, a fixed effect to adjust for clinic-level catchment area, and an AR(1) structure to account for autocorrelation in residual errors. Gray dashed lines are 95% confidence intervals around the fitted mean. Red lines are placed at the final month before the start (May 2014) and end (April 2015) of the EVD outbreak in Liberia.

**Fig 6:**
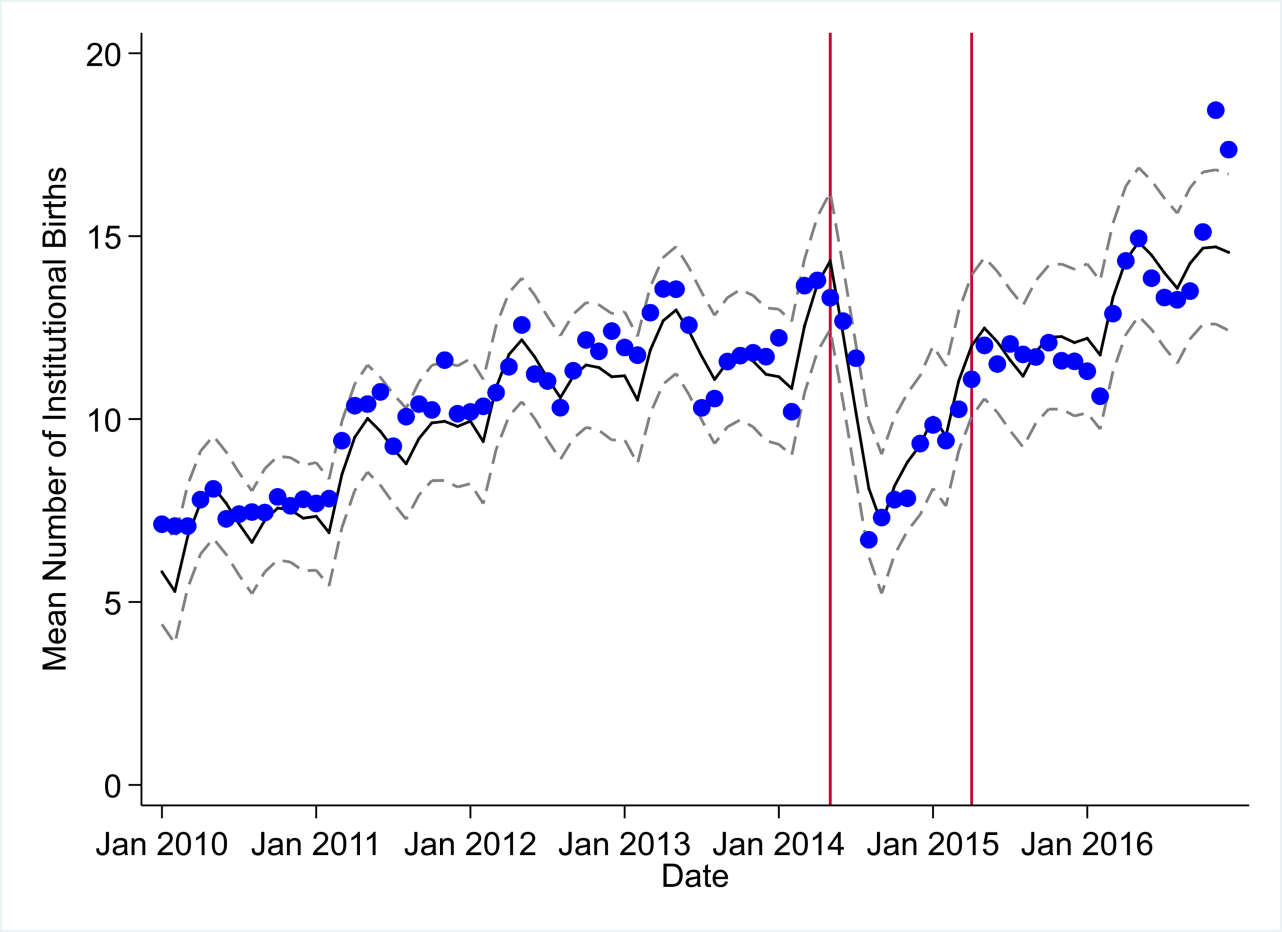
Mean trends and system losses due to Ebola virus disease (EVD) outbreak (June 2014–April 2015) for institutional births in a census of 379 clinics providing services in Liberia from 2010–2016, excluding Montserrado County. The black solid line represents the fitted mean from a linear mixed model using a segmented regression parameterization, random intercepts and slopes by facility, monthly indicator variables to adjust for seasonality, a fixed effect to adjust for clinic-level catchment area, and an AR(1) structure to account for autocorrelation in residual errors. Gray dashed lines are 95% confidence intervals around the fitted mean. Red lines are placed at the final month before the start (May 2014) and end (April 2015) of the EVD outbreak in Liberia.

**Fig 7:**
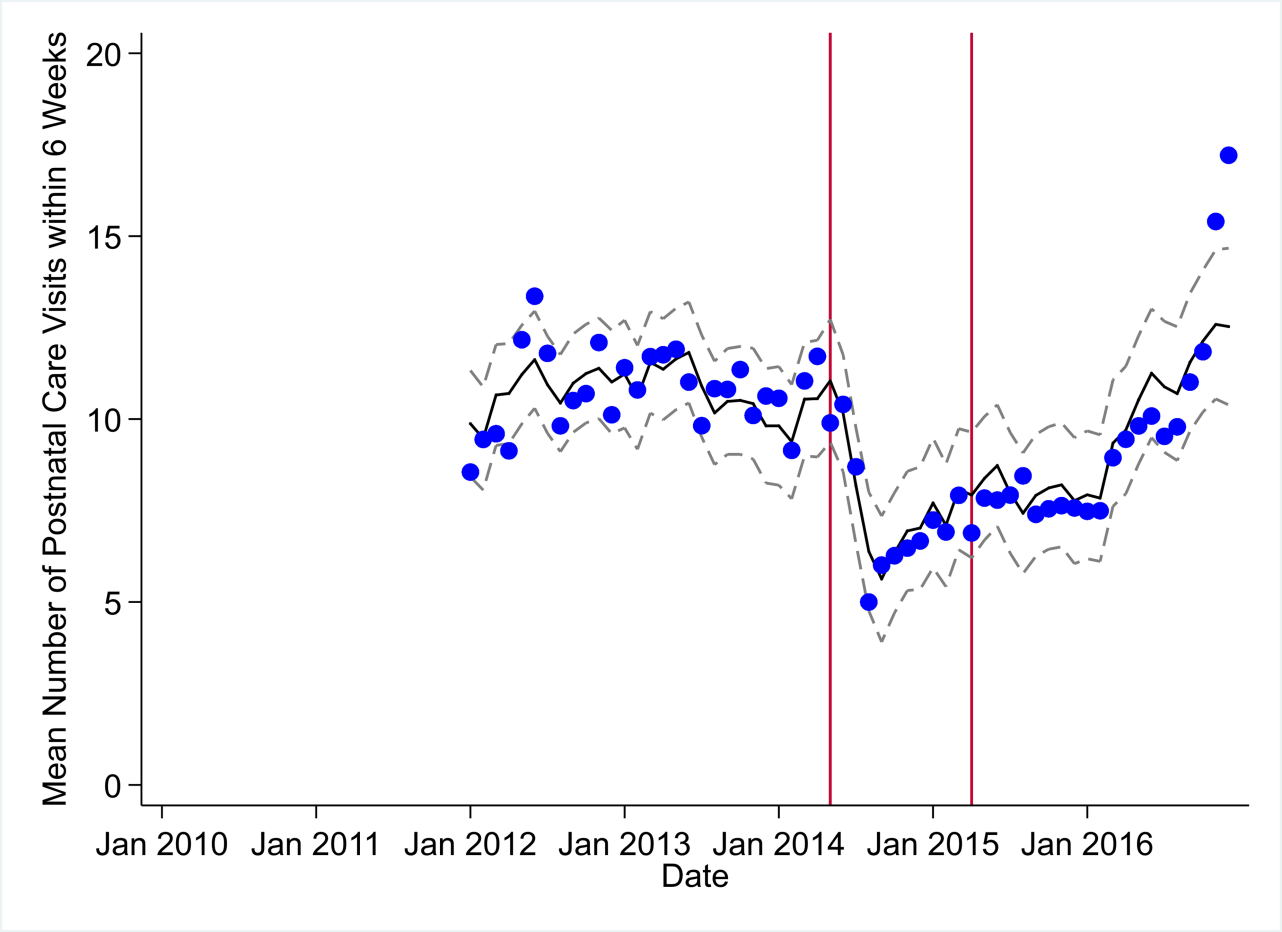
Mean trends and system losses due to Ebola virus disease (EVD) outbreak (June 2014–April 2015) for postnatal care within 6 weeks in a census of 379 clinics providing services in Liberia from 2010–2016, excluding Montserrado County. The black solid line represents the fitted mean from a linear mixed model using a segmented regression parameterization, random intercepts and slopes by facility, monthly indicator variables to adjust for seasonality, a fixed effect to adjust for clinic-level catchment area, and an AR(1) structure to account for autocorrelation in residual errors. Gray dashed lines are 95% confidence intervals around the fitted mean. Red lines are placed at the final month before the start (May 2014) and end (April 2015) of the EVD outbreak in Liberia.

**Fig 8:**
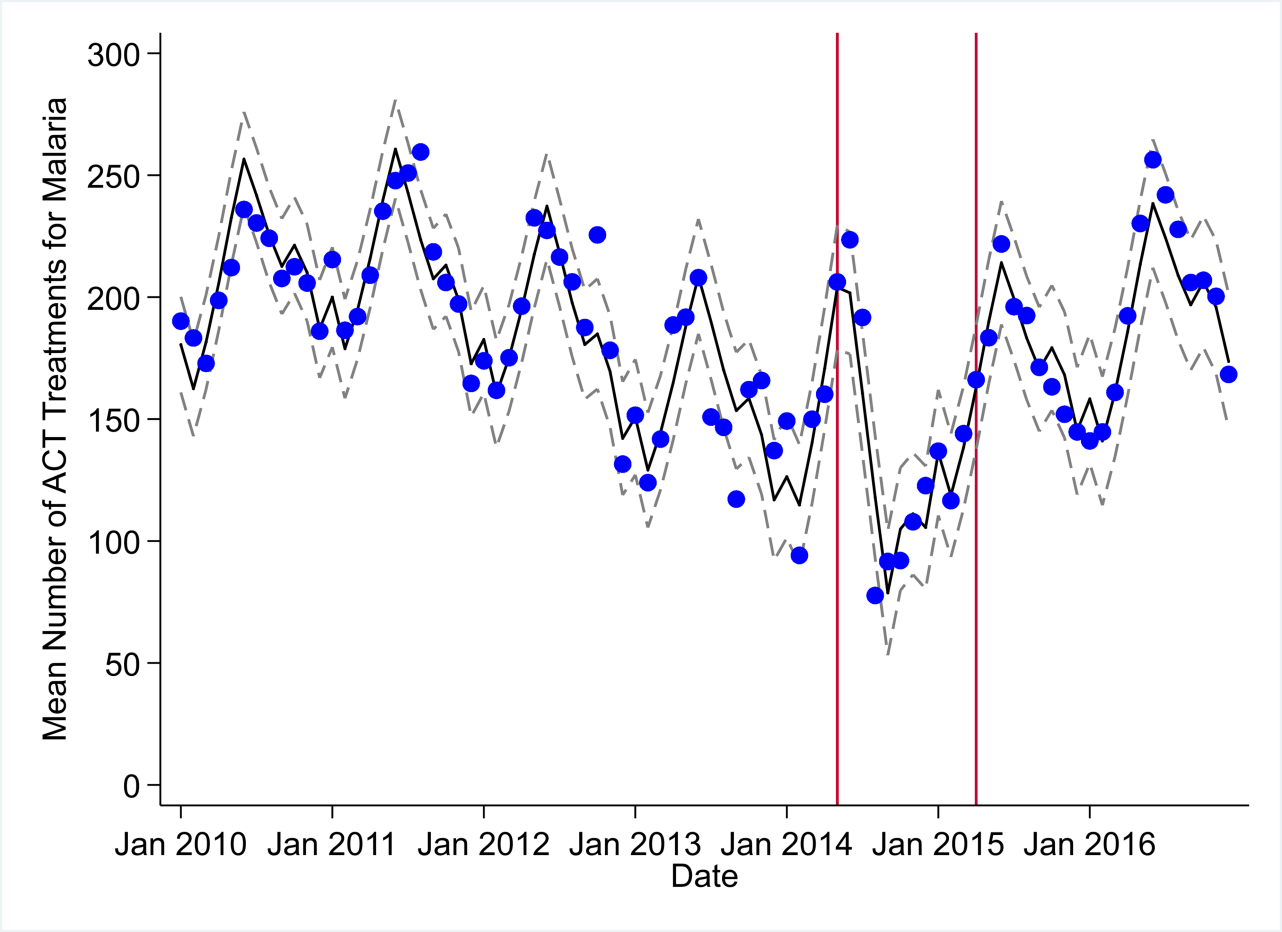
Mean trends and system losses due to Ebola virus disease (EVD) outbreak (June 2014–April 2015) for artemisinin-based combination therapy (ACT) treatments for malaria in a census of 379 clinics providing services in Liberia from 2010–2016, excluding Montserrado County. The black solid line represents the fitted mean from a linear mixed model using a segmented regression parameterization, random intercepts and slopes by facility, monthly indicator variables to adjust for seasonality, a fixed effect to adjust for clinic-level catchment area, and an AR(1) structure to account for autocorrelation in residual errors. Gray dashed lines are 95% confidence intervals around the fitted mean. Red lines are placed at the final month before the start (May 2014) and end (April 2015) of the EVD outbreak in Liberia.

**Fig 9:**
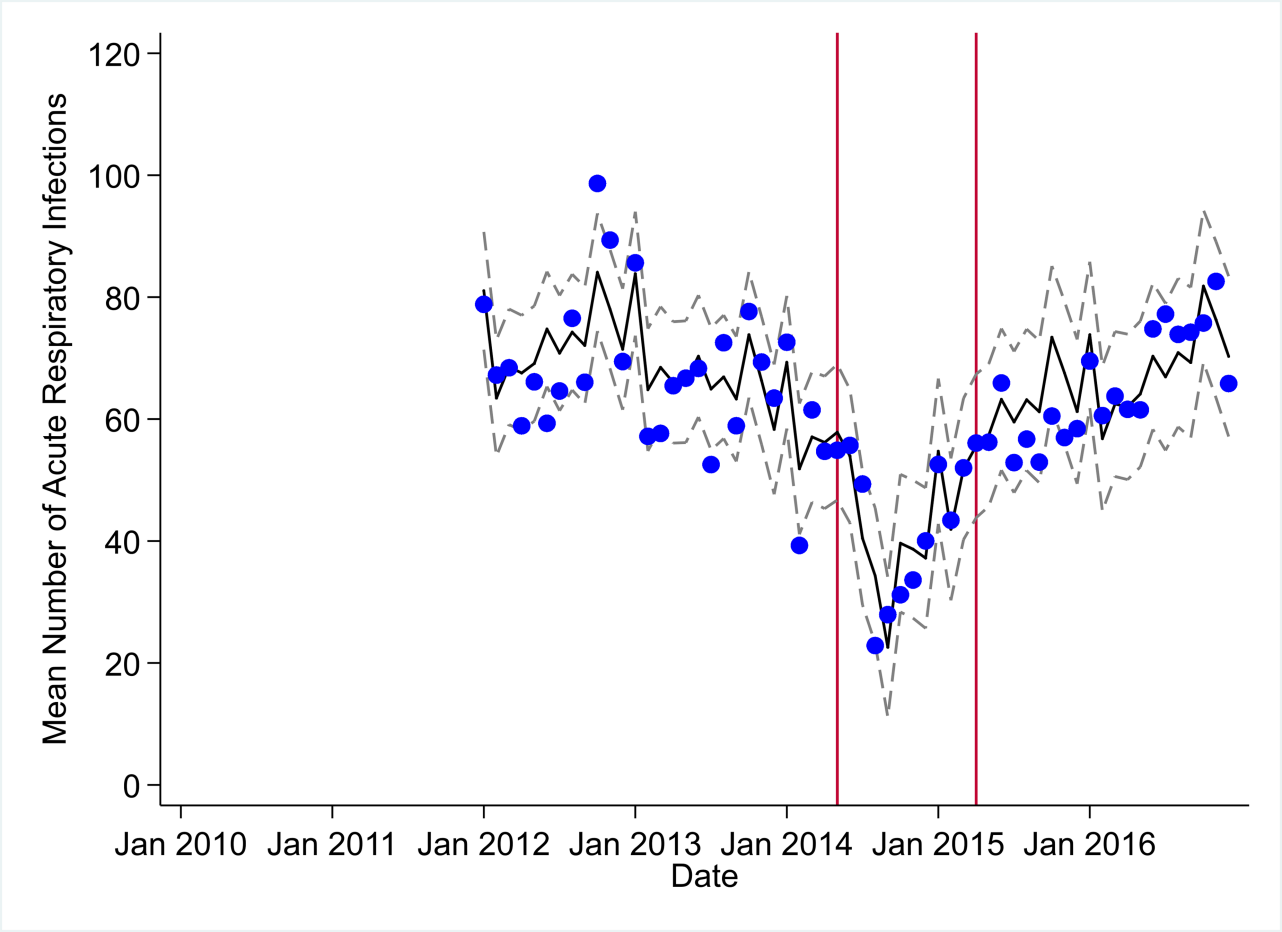
Mean trends and system losses due to Ebola virus disease (EVD) outbreak (June 2014–April 2015) for acute respiratory infections treated in a census of 379 clinics providing services in Liberia from 2010–2016, excluding Montserrado County. The black solid line represents the fitted mean from a linear mixed model using a segmented regression parameterization, random intercepts and slopes by facility, monthly indicator variables to adjust for seasonality, a fixed effect to adjust for clinic-level catchment area, and an AR(1) structure to account for autocorrelation in residual errors. Gray dashed lines are 95% confidence intervals around the fitted mean. Red lines are placed at the final month before the start (May 2014) and end (April 2015) of the EVD outbreak in Liberia.

**Fig 10:**
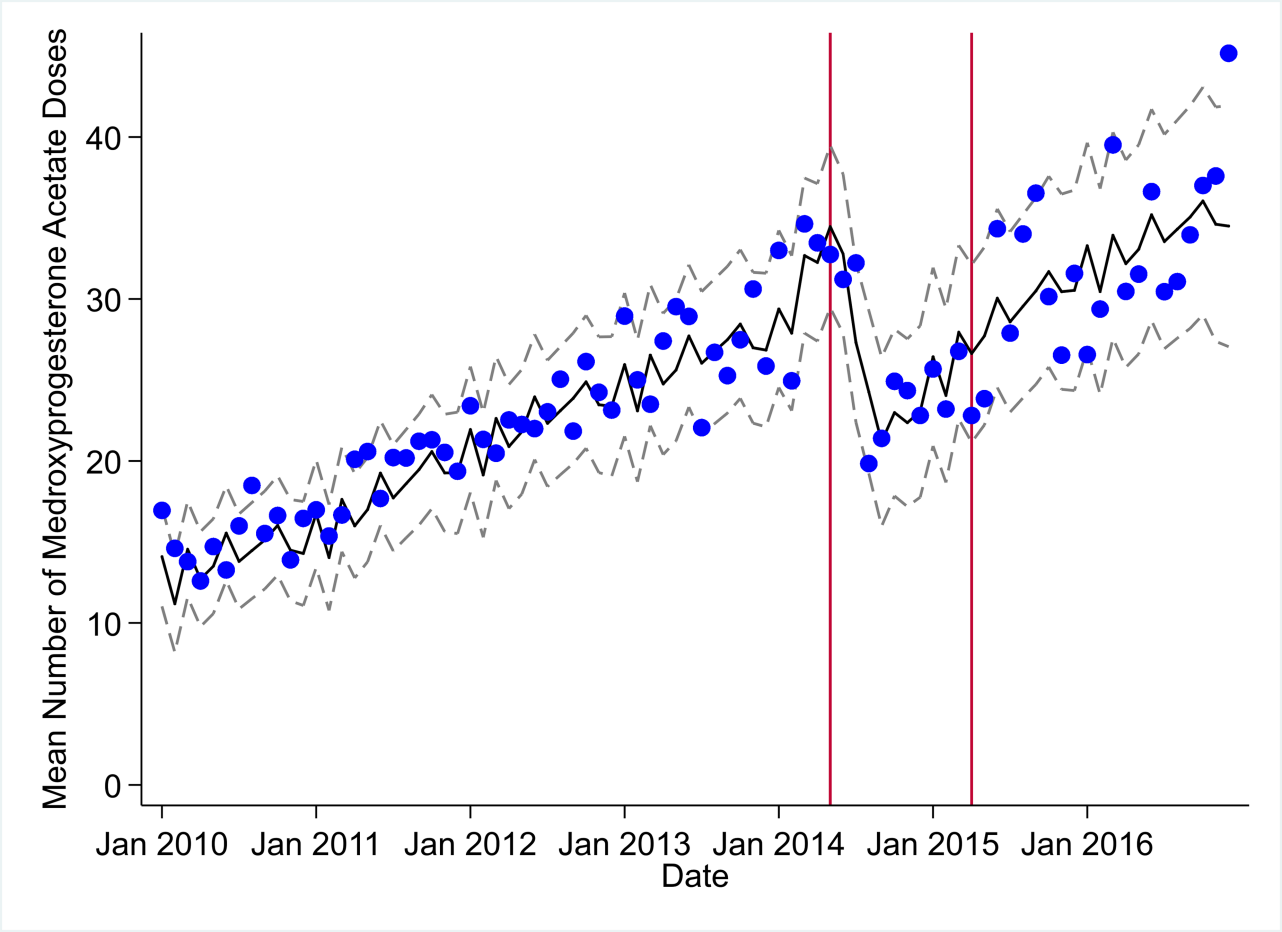
Mean trends and system losses due to Ebola virus disease (EVD) outbreak (June 2014–April 2015) for medroxyprogesterone acetate doses in a census of 379 clinics providing services in Liberia from 2010–2016, excluding Montserrado County. The black solid line represents the fitted mean from a linear mixed model using a segmented regression parameterization, random intercepts and slopes by facility, monthly indicator variables to adjust for seasonality, a fixed effect to adjust for clinic-level catchment area, and an AR(1) structure to account for autocorrelation in residual errors. Gray dashed lines are 95% confidence intervals around the fitted mean. Red lines are placed at the final month before the start (May 2014) and end (April 2015) of the EVD outbreak in Liberia.

Pre-EVD trends were heterogeneous by indicator. Clinic visits, first ANC visits, and ACT malaria treatments all showed strong and consistent decreases across the pre-EVD period, resulting in decreases in system outputs of −21.9% (95% CI: −27.6%, −16.3%, *p <* 0.001), −30.8% (95% CI: −38.4%, −23.3%, *p <* 0.001), and −30.0% (95% CI: −39.2%, −20.8%, *p <* 0.001), respectively, comparing January 2010 to January 2014. Other indicators showed strong and consistent increases, including institutional births (+91.6%; 95% CI: +61.4%, +121.9%, *p <* 0.001) and medroxyprogesterone acetate doses (+108.5%; 95% CI: +70.0, +146.9, *p <* 0.001). Remaining indicators showed mixed trends, with some years of significant increases and some years of significant decreases (see Tables 3–6; see Figs 1–10). Of note is that official MoH clinic catchment populations increased an average of 8.6% from January 2011 to January 2014.

**Table 3:** Parameter estimates and system losses due to Ebola virus disease (EVD) outbreak (June 2014–April 2015) for clinic visits, bacille Calmette–Guérin (BCG) vaccinations, and measles vaccinations across a census of clinics providing services in Liberia excluding Montserrado County, 2010–2016.

Time period	Clinic visits			BCG vaccinations			Measles vaccinations		
	β (95% CI)*	*p*-Value	Mean (95% CI)^†^	β (95% CI)*	*p*-Value	Mean (95% CI)^†^	β (95% CI)*	*p*-Value	Mean (95% CI)^†^
**Overall yearly trends pre-EVD**									
Monthly change Jan–Dec 2010	−2.5 (−4.5, −0.50)	0.014	746.6 (678.2, 815.1)	−0.31 (−0.41, −0.21)	<0.001	23.4 (21.0, 25.7)	0.02 (−0.14, 0.19	0.80	18.6 (17.2, 20.1)
Monthly change Jan–Dec 2011	−3.8 (−6.1, −1.5)	0.001	712.7 (644.7, 780.8)	0.23 (0.095, 0.36)	0.001	22.4 (19.9, 24.9)	−0.17 (−0.32, −0.03)	0.02	18.5 (17.0, 20.0)
Monthly change Jan–Dec 2012	−5.4 (−8.0, −2.9)	<0.001	665.3 (601.4, 729.2)	−0.21 (−0.32, −0.11)	<0.001	23.3 (20.4, 26.2)	0.23 (0.09, 0.37)	0.001	19.5 (17.7, 21.2)
Monthly change Jan–Dec 2013	−4.4 (−6.5, −2.2)	<0.001	616.6 (558.1, 675.0)	−0.16 (−0.27, −0.06)	0.002	21.1 (18.5, 23.6)	−0.36 (−0.51, −0.21)	<0.001	17.2 (15.5, 19.0)
Monthly change Jan–May 2014	6.4 (−0.26, 13.1)	0.06	592.6 (531.3, 654.0)	0.63 (0.30, 0.96)	<0.001	21.9 (19.1, 24.6)	0.73 (0.17, 1.3)	0.01	17.5 (15.6, 19.4)
**EVD outbreak (Jun 2014–Apr 2015)**									
Monthly change first 4 months (Jun–Sep 2014)	−81.4 (−89.2, −73.5)	<0.001	488.2 (439.8, 536.6)	−3.0 (−3.4, −2.6)	<0.001	15.9 (14.0, 17.9)	−3.1 (−3.8, −2.5)	<0.001	11.3 (10.2, 12.5)
Monthly change middle 3 months (Oct–Dec 2014)	55.3 (47.2, 63.3)	<0.001	394.2 (351.6, 436.8)	2.2 (1.8, 2.6)	<0.001	15.7 (13.5, 18.0)	4.9 (4.2, 5.6)	<0.001	16.8 (14.3, 19.3)
Monthly change last 4 months (Jan–Apr 2015)	−0.96 (−10.5, 8.6)	0.84	502.3 (452.9, 551.6)	−0.83 (−1.3, −0.35)	0.001	18.4 (16.1, 20.7)	−4.1 (−4.9, −3.3)	<0.001	19.6 (16.0, 23.2)
**Post-EVD (May 2015–Dec 2016)**									
Monthly change May–Dec 2015	9.2 (6.0, 12.5)	<0.001	562.8 (506.4, 619.3)	0.29 (0.15, 0.42)	<0.001	18.6 (16.2, 20.9)	0.28 (0.42, 0.52)	0.02	13.8 (12.3, 15.4)
Monthly change Jan–Dec 2016	3.4 (0.72, 6.0)	0.013	640.6 (577.6, 703.7)	0.30 (0.14, 0.46)	<0.001	22.1 (19.4, 24.8)	0.43 (0.24, 0.63)	<0.001	18.7 (16.6, 20.7)

*All analyses include fixed-effect control variables for monthly indicator variables and facility-level catchment population.

^†^Mean represents average monthly number of system outputs across facilities included in a given analysis and across a given time period using a linear mixed model controlling for clustering at the facility level and employing an AR(1) structure to control for autocorrelation in residual errors.

**Table 4:** Parameter estimates and system losses due to Ebola virus disease (EVD) outbreak (June 2014–April 2015) for first pentavalent vaccinations, first antenatal care visits, and institutional births across a census of clinics providing services in Liberia excluding Montserrado County, 2010–2016.

Time period	First pentavalent vaccinations			First antenatal care visits			Institutional births		
	β (95% CI)*	*p*-Value	Mean (95% CI)^†^	β (95% CI)*	*p*-Value	Mean (95% CI)^†^	β (95% CI)*	*p*-Value	Mean (95% CI)^†^
**Overall yearly trends pre-EVD**									
Monthly change Jan–Dec 2010	−0.19 (−0.29, −0.09)	<0.001	22.4 (19.9, 24.8)	−0.20 (−0.32, −0.09)	0.001	27.6 (23.8, 31.5)	0.12 (0.06, 0.18)	<0.001	6.8 (5.4, 8.2)
Monthly change Jan–Dec 2011	0.16 (0.037, 0.29)	0.011	21.4 (19.3, 23.5)	−0.49 (−0.68, −0.30)	<0.001	24.5 (21.1, 28.0)	0.21 (0.12, 0.29)	<0.001	9.1 (7.4, 10.7)
Monthly change Jan–Dec 2012	−0.06 (−0.19, 0.07)	0.35	23.2 (20.9, 25.5)	−0.22 (−0.32, −0.13)	<0.001	20.2 (17.0, 23.4)	0.09 (0.04, 0.15)	0.001	10.7 (8.7, 12.6)
Monthly change Jan–Dec 2013	−0.17 (−0.26, −0.07)	0.001	21.9 (19.7, 24.0)	−0.09 (−0.22, 0.04)	0.16	18.8 (15.8, 21.8)	−0.01 (−0.08, 0.06)	0.69	11.4 (9.6, 13.2)
Monthly change Jan–May 2014	0.31 (−0.03, 0.65)	0.07	21.1 (18.7, 23.4)	−0.08 (−0.48, 0.32)	0.69	18.7 (15.9, 21.4)	0.33 (0.14, 0.52)	0.001	12.3 (10.4, 14.1)
**EVD outbreak (Jun 2014–Apr 2015)**									
Monthly change first 4 months (Jun–Sep 2014)	−2.7 (−3.1, −2.3)	<0.001	16.0 (14.3, 17.7)	−1.8 (−2.3, −1.3)	<0.001	12.9 (11.1, 14.8)	−1.5 (−1.7, −1.2)	<0.001	9.9 (8.4, 11.4)
Monthly change middle 3 months (Oct–Dec 2014)	2.3 (1.9, 2.8)	<0.001	16.7 (14.8, 18.5)	1.7 (1.2, 2.2)	<0.001	14.6 (12.3, 16.9)	0.81 (0.59, 1.0)	<0.001	8.2 (6.9, 9.5)
Monthly change last 4 months (Jan–Apr 2015)	−0.92 (−1.4, −0.43)	<0.001	19.3 (17.4, 21.2)	0.03 (−0.56, 0.61)	0.93	18.9 (16.1, 21.6)	0.14 (−0.13, 0.40)	0.32	10.1 (8.5, 11.7)
**Post-EVD (May 2015–Dec 2016)**									
Monthly change May–Dec 2015	0.28 (0.14, 0.41)	<0.001	18.9 (16.9, 20.9)	−0.07 (−0.24, 0.09)	0.38	17.3 (14.5, 20.1)	0.18 (0.09, 0.27)	<0.001	11.7 (9.8, 13.7)
Monthly change Jan–Dec 2016	0.60 (0.41, 0.79)	<0.001	24.1 (21.6, 26.6)	0.19 (0.04, 0.35)	0.014	19.1 (16.1, 22.0)	0.20 (0.11, 0.28)	<0.001	13.3 (11.2, 15.4)

*All analyses include fixed-effect control variables for monthly indicator variables and facility-level catchment population.

^†^Mean represents average monthly number of system outputs across facilities included in a given analysis and across a given time period using a linear mixed model controlling for clustering at the facility level and employing an AR(1) structure to control for autocorrelation in residual errors.

**Table 5:** Parameter estimates and system losses due to Ebola virus disease (EVD) outbreak (June 2014–April 2015) for postnatal care visits within 6 weeks, artemisinin-based combination therapy (ACT) treatments for malaria, and acute respiratory infections treated across a census of clinics providing services in Liberia excluding Montserrado County, 2010–2016.

Time period	Postnatal care within 6 weeks^‡^			ACT treatments for malaria			Acute respiratory infections treated^‡^		
	β (95% CI)*	*p*-Value	Mean (95% CI)^†^	β (95% CI)*	*p*-Value	Mean (95% CI)^†^	β (95% CI)*	*p*-Value	Mean (95% CI)^†^
**Overall yearly trends pre-EVD**									
Monthly change Jan–Dec 2010	n/a^‡^	n/a^‡^	n/a^‡^	1.4 (0.60, 2.2)	0.001	204.2 (185.6, 222.8)	n/a^‡^	n/a^‡^	n/a^‡^
Monthly change Jan–Dec 2011	n/a^‡^	n/a^‡^	n/a^‡^	−1.6 (−2.6, −0.69)	0.001	211.1 (191.4, 230.9)	n/a^‡^	n/a^‡^	n/a^‡^
Monthly change Jan–Dec 2012	0.10 (0.006, 0.20)	0.04	11.0 (8.9, 13.1)	−2.8 (−3.8, −1.9)	<0.001	186.9 (169.7, 204.1)	0.19 (−0.28, 0.66)	0.43	72.4 (63.6, 81.1)
Monthly change Jan–Dec 2013	−0.13 (−0.24, 0.02)	0.016	10.6 (9.3, 11.9)	−2.3 (−3.2, −1.3)	<0.001	155.7 (142.9, 168.7)	−1.3 (−1.7, −0.77)	<0.001	66.1 (57.9, 74.2)
Monthly change Jan–May 2014	0.08 (−0.25, 0.40)	0.65	9.9 (8.6, 11.2)	7.8 (5.1, 10.5)	<0.001	159.6 (145.4, 173.7)	0.31 (−1.2, 1.8)	0.68	54.9 (47.5, 62.4)
**EVD outbreak (Jun 2014–Apr 2015)**									
Monthly change first 4 months (Jun–Sep 2014)	−1.2 (−1.6, 0.81)	<0.001	7.8 (6.4, 9.1)	−24.9 (−28.0, −21.8)	<0.001	153.8 (141.0, 166.6)	−9.4 (−11.1, −7.7)	<0.001	41.0 (35.3, 46.7)
Monthly change middle 3 months (Oct–Dec 2014)	0.56 (0.16, 0.95)	0.006	6.3 (5.1, 7.5)	18.9 (15.7, 22.1)	<0.001	106.8 (97.7, 115.9)	5.3 (3.5, 7.0)	<0.001	35.5 (29.8, 41.2)
Monthly change last 4 months (Jan–Apr 2015)	−0.10 (−0.57, 0.37)	0.67	7.2 (6.1, 8.3)	2.0 (−1.9, 5.8)	0.32	142.0 (129.9, 154.1)	5.0 (2.9, 7.0)	<0.001	51.6 (43.9, 59.3)
**Post-EVD (May 2015–Dec 2016)**									
Monthly change May–Dec 2015	0.04 (−0.09, 0.18)	0.52	7.7 (6.6, 8.8)	1.5 (0.24, 2.8)	0.02	176.0 (161.5, 190.5)	0.41 (−0.21, 1.0)	0.20	58.1 (49.6, 66.7)
Monthly change Jan–Dec 2016	0.42 (0.27, 0.56)	<0.001	9.9 (8.6, 11.2)	2.2 (1.1, 3.4)	<0.001	188.6 (171.9, 205.3)	0.74 (0.09, 1.4)	0.026	68.3 (60.3, 76.3)

*All analyses include fixed-effect control variables for monthly indicator variables and facility-level catchment population.

^†^Mean represents average monthly number of system outputs across facilities included in a given analysis and across a given time period using a linear mixed model controlling for clustering at the facility level and employing an AR(1) structure to control for autocorrelation in residual errors.

^‡^Postnatal care within 6 weeks and acute respiratory infections treated were analyzed from January 2012 to December 2016 due to inconsistent recording in the Ministry of Health system prior to January 2012.

n/a, not applicable.

**Table 6:** Parameter estimates and system losses due to Ebola virus disease (EVD) outbreak (June 2014–April 2015) for medroxyprogesterone acetate doses across a census of clinics providing services in Liberia excluding Montserrado County, 2010–2016.

Time period	Medroxyprogesterone acetate doses		
	β (95% CI)*	*p*-Value	Mean (95% CI)^†^
**Overall yearly trends pre-EVD**			
Monthly change Jan–Dec 2010	0.21 (−0.0001, 0.41)	0.05	14.5 (12.0, 17.0)
Monthly change Jan–Dec 2011	0.42 (0.18, 0.66)	0.001	17.8 (14.6, 21.0)
Monthly change Jan–Dec 2012	0.32 (0.09, 0.55)	0.006	22.1 (17.9, 26.3)
Monthly change Jan–Dec 2013	0.27 (0.04, 0.50)	0.02	26.1 (21.5, 30.7)
Monthly change Jan–May 2014	1.6 (0.96, 2.3)	<0.001	31.3 (26.0, 36.5)
**EVD outbreak (Jun 2014–Apr 2015)**			
Monthly change first 4 months (Jun–Sep 2014)	−3.5 (−4.3, −2.7)	<0.001	26.1 (22.6, 29.6)
Monthly change middle 3 months (Oct–Dec 2014)	1.1 (0.29, 1.9)	0.008	23.8 (20.0, 27.6)
Monthly change last 4 months (Jan–Apr 2015)	0.74 (−0.24, 1.7)	0.14	24.4 (20.6, 28.2)
**Post-EVD (May 2015–Dec 2016)**			
Monthly change May–Dec 2015	0.50 (0.11, 0.88)	0.01	30.5 (24.7, 36.3)
Monthly change Jan–Dec 2016	0.30 (−0.10, 0.70)	0.14	32.8 (27.7, 37.9)

*All analyses include fixed-effect control variables for monthly indicator variables and facility-level catchment population.

^†^Mean represents average monthly number of system outputs across facilities included in a given analysis and across a given time period using a linear mixed model controlling for clustering at the facility level and employing an AR(1) structure to control for autocorrelation in residual errors.

All indicators, except measles vaccinations, exhibited significant seasonal effects. Clinic visits were most common in the month of June, first ANC visits were most common in January, institutional births were most common in May, ACT malaria treatments were most common in June, and the most ARIs were treated in October.

### Early effects of the EVD outbreak (June 2014–September 2014)

All indicators had statistically significant and clinically important decreases in system outputs during the first 4 months of the EVD outbreak (June 2014–September 2014) (Tables 3–6; Figs 1–10). Using raw means, all indicators had their lowest system outputs in the month of August 2014. Model-fitted decreases in outputs comparing the end of the initial EVD period (September 2014) to May 2014 (pre-EVD) ranged in magnitude from a 67.3% decrease in measles vaccinations (95% CI: −77.9%, −56.8%, *p <* 0.001) and a 61.4% decrease in ACT malaria treatments (95% CI: −69.0%, −53.8%, *p <* 0.001) to a 35.2% decrease in first ANC visits (95% CI: −45.8%, −24.7%, *p <* 0.001) and a 38.5% decrease in medroxyprogesterone acetate doses (95% CI: −47.6%, −29.5%, *p <* 0.001). All remaining indicators showed decreases in system outputs ranging from a 48.7% decrease in first pentavalent doses (95% CI: −56.0%, −41.5%, *p <* 0.001) to a 61.0% decrease in ARIs treated (95% CI: −73.6%, −48.3%, *p <* 0.001).

### System resilience during the EVD outbreak (October 2014–April 2015)

Following the nadir of system outputs in August 2014, all indicators showed statistically significant increases from October 2014 to December 2014—beginning the recovery of health system outputs to pre-EVD levels (Tables 3–6). By January 2015, measles vaccination had increased to 162.9% of the pre-EVD levels from January 2014 (95% CI: 144.4%, 181.3%, *p <* 0.001), likely due to targeted vaccination campaigns. Furthermore, all other indicators increased from the nadir to a range of 107.8% of pre-EVD (January 2014) levels for ACT treatments (95% CI: 91.1%, 116.4%, *p <* 0.001) to 78.5% for PNC visits (95% CI: 65.9%, 91.1%, *p <* 0.001). Following these initial system increases, indicators showed mixed progress over the next 4 months (January 2015–April 2015), with clinic visits, first ANC visits, institutional births, PNC visits, ACT treatments for malaria, and medroxyprogesterone acetate doses showing no significant increases. BCG and first pentavalent vaccinations also showed significant decreases during this time period. In contrast to all other indicators, treatment for ARIs continued to significantly increase during this time period. By May 2015, measles vaccinations normalized to 74.2% of pre-EVD (May 2014) levels (95% CI: 64.6%, 83.8%, *p <* 0.001) as targeted campaigns ended.

### Post-EVD trends (May 2015–December 2016)

All indicators had significant positive trends during the post-EVD period, with every system output exceeding pre-Ebola forecasted trends for 3 consecutive months by November 2016. Measles vaccinations exceeded pre-Ebola forecasted trends earliest, achieving the 3-month benchmark in February 2015 even prior to the EVD outbreak finishing (Table 2). Other indicators notable for their rapid recovery to pre-EVD levels included ACT treatments for malaria (recovered by May 2015), medroxyprogesterone acetate doses (recovered by November 2015), and institutional births (recovered by December 2015). First ANC visits, PNC visits within 6 weeks, and ARIs treated did not show significant increases during the initial 8-month period post-EVD (May–December 2015); however, all showed significant increases from January to December 2016. PNC visits and first ANC visits took longest to recover to pre-EVD levels, achieving 3 months of output exceeding the pre-Ebola forecast in September and November 2016, respectively.

### Estimates of system outputs lost due to the EVD outbreak

Health system outputs lost due to the EVD outbreak were large and sustained for most indicators. Prior to exceeding pre-EVD forecasted trends for 3 months, we estimate a cumulative loss of −776,110 clinic visits (95% CI: −1,480,896, −101,357, *p =* 0.030); −24,449 BCG vaccinations (95% CI: −45,947, −2,020, *p =* 0.032); −9,129 measles vaccinations (95% CI: −12,312, −5,659, *p <* 0.001); −23,077 first pentavalent vaccinations (95% CI: −47,704, +1,706, *p =* 0.058); −13,189 first ANC visits (95% CI: −49,765, +23,320, *p =* 0.482); −7,243 institutional births (95% CI: −15,554, +1,502, *p =* 0.124); −17,191 PNC visits within 6 weeks (95% CI: −28,344, −5,775, *p =* 0.002); −101,857 ACT malaria treatments (95% CI: −205,839, −2,139, *p =* 0.044); −54,549 ARIs treated (95% CI: −111,392, +2,264, *p =* 0.058); and −15,889 medroxyprogesterone acetate doses (95% CI: −41,066, +9,314, *p =* 0.232) due to the EVD outbreak (Table 2). When tabulating cumulative system losses compared to pre-EVD forecasted trends through December 2014, all indicators except first ANC visits and medroxyprogesterone acetate doses had statistically significant cumulative losses of system outputs.

All indicators exceeded pre-EVD levels by November 2016 and showed sustained, statistically significant positive trends post-EVD. However, only ACT treatments for malaria had a point estimate of system output change that exceeded zero compared to pre-EVD forecasted trends through December 2016—showing an excess of +78,583 treatments (95% CI: −309,417, +450,661, *p =* 0.634), although it is important to note that this confidence interval is wide and contains zero. Comparing model-fitted ACT malaria treatments from December 2013 and December 2016, cases significantly increased by +49.2% (95% CI: +33.9%, +64.5%, *p <* 0.001). Compared to pre-EVD forecasted trends, PNC visits within 6 weeks was the only indicator showing a statistically significant change in outputs through December 2016, showing a loss of −15,144 PNC visits within 6 weeks (95% CI: −29,453, −787, *p =* 0.040). With the exception of ACT malaria treatments, all other indicators had large negative point estimates for total change in cumulative system outputs through December 2016, although all confidence intervals are large and contain zero (except PNC visits within 6 weeks; Table 2).

## Discussion

To our knowledge, this study is the first analytical assessment of public-sector primary healthcare delivery before, during, and after the 2014–2015 EVD outbreak. This study uses data from 7 years of service delivery in Liberia (excluding Montserrado County) to accurately estimate trends before, during, and after the outbreak, as well as forecast system losses attributable to the EVD outbreak. We observed large and significant changes in the delivery of public-sector primary healthcare across Liberia during and after the EVD outbreak. It took only 4 months to lose between 35% and 67% of essential primary care health system outputs across Liberian clinics after the beginning of the EVD outbreak (with the time period of the outbreak defined as June 2014–April 2015). The Liberian health system showed early resilience during the EVD outbreak, with all primary healthcare indicators showing increases from October to December 2014. Unlike findings from recent studies in Guinea [4], our analyses show that, through December 2016, primary healthcare delivery across Liberia has shown significant evidence of recovery from the EVD outbreak. However, due to the large magnitude of health system output losses during the EVD outbreak, there remain estimated net losses of tens of thousands of key childhood vaccinations and essential MCH consultations and hundreds of thousands of clinic visits.

In addition, the EVD outbreak appears to have reversed multiple-year trends of decreases in the number of malaria cases. We hypothesize that the loss of over 100,000 treatments for malaria during the EVD outbreak may have contributed to an excess of malaria cases after the EVD outbreak, and a return to numbers of malaria cases not seen since 2011. Our analyses suggest that malaria cases have increased by almost 50% comparing December 2013 before the EVD outbreak to December 2016. These measurements corroborate previous mathematical models that have hypothesized a large increase in malaria cases due to the disruption of malaria case management in EVD-affected countries [11,12,24]. In addition to the disruption of acute malaria treatment, the observed increase in malaria cases may be partially attributable to interruption of prevention activities (e.g., bednet distribution and utilization programs), as well as an increased fraction of malaria cases being treated post-EVD compared to pre-EVD.

We found that it took 23 months after the beginning of the EVD outbreak, and 11 months after Liberia was declared free of EVD, for health-seeking behavior to return to pre-Ebola levels, leading to a cumulative loss of more than 770,000 clinic visits. This gap in primary care service delivery lasted longer for first ANC visits, PNC visits within 6 weeks, and BCG and first pentavalent vaccinations. While some indicators such as clinic visits, ANC visits, PNC visits, and vaccinations may potentially be “made up” after the EVD epidemic (although deferred care could still be detrimental), other indicators such as institutional births, treatment for episodes of malaria and ARI, and medroxyprogesterone acetate doses that are inherently time-limited cannot be made up through increased access to care post-EVD. In the present paper, we calculated net losses of health system outputs for all 10 primary care indicators, with the caveat that increased access to care for acute and time-limited indicators does not offset the deleterious effects of gaps in treatment access during the EVD outbreak.

These findings suggest the need for the development of best practices, coordinated responses, and larger global investments in public-sector health system strengthening efforts when responding to future global public health emergencies [34]. The emerging evidence from Sierra Leone [2,16,17,22], Guinea [3,4,11,15], and Liberia ([6–10] and our study) suggests that, in terms of morbidity and mortality, the collateral effects from the EVD outbreak on public-sector primary healthcare delivery will greatly exceed the direct effects from EVD infection. Analyses using population-based surveys in Liberia suggest that distrust of the government and the health system was the primary source of reduced health service demand during the EVD outbreak, rather than supply-side factors [29]. Thus, during future health system emergencies, policymakers should address demand-side factors during periods of crisis. In addition, sustained and long-term investments in public-sector health system strengthening is needed to ensure that EVD-affected countries have the resilience to detect, treat, and eradicate future emerging epidemics prior to large-scale spread, all while maintaining the delivery of life-saving public-sector primary healthcare.

Prior to the EVD outbreak, Liberia had one of the highest rates of maternal mortality in the world, with a rate of 1,072 deaths per 100,000 live births according to the 2013 Demographic and Health Survey (DHS) [32]—representing an 8% increase from 2007 DHS estimates [35]. Nationwide, maternal deaths are estimated to represent 38% of all deaths to women aged 15–49 years [32]. As of 2013, community-level estimates of institutional birth coverage (56%) and use of any modern contraceptive method (21%) remained low [32]. For these reasons, it was positive to document the large increases in institutional births (+92%) and medroxyprogesterone acetate doses (+109%) from 2010 to 2014 in our study. These increases are corroborated by community survey data showing a more than 50% increase in institutional births and a 130% increase in injectable contraceptive use from 2007 to 2013 [32,35]. Given these successes, it is especially concerning to see the loss of 7,243 institutional births, along with gaps in other essential MCH services (estimated losses of 13,189 first ANC visits, 17,191 PNC visits within 6 weeks, and 15,889 medroxyprogesterone acetate doses), that occurred during the EVD outbreak, all of which continue to have point estimates of net losses in system outputs through December 2016, over 20 months post-EVD. However, it is important to note that PNC visits within 6 weeks is the only indicator with a confidence interval of lost system outputs through December 2016 that does not contain zero.

The loss of medroxyprogesterone acetate doses may have lasting negative impacts on MCH across Liberia as unintended pregnancies are linked to elevated risks of low birth weight, child malnutrition and mortality, and maternal mortality [36]. The disruption of access to long-acting reversible contraception during the EVD outbreak could also partially explain the observed increases in recorded births post-EVD [37]. The slow recovery of first ANC visits and PNC visits to pre-EVD levels may warrant targeted interventions, especially considering the persistent gaps in PNC coverage pre-EVD [32], as well as the essential role that first ANC visits play in the health of mother and child. Given the near universal coverage (>95%) of at least 1 ANC visit nationwide [32], the observed decreases in first ANC visits before the EVD outbreak could at least partially reflect declining total fertility rates [32], although we hypothesize that the majority of this decrease is due to improved routine data management, such as better tracking of repeat ANC visits. A recent study in the capital city of Monrovia (which was excluded from the present analyses) showed that during the EVD outbreak, deliveries in public clinics decreased dramatically and were substituted by a similar magnitude increase in deliveries in private clinics [30].

In regards to child vaccination, prior to the EVD outbreak, reported numbers of BCG, first pentavalent, and measles vaccinations were relatively stable, with some decreases also hypothesized to be due to decreasing fertility rates. The EVD outbreak led to large losses in vaccination outputs, leaving young children at significant risk for infection with life-threatening illnesses, as well as potentially putting adults at risk through the breakdown of community-level herd immunity. Overall, we recorded significant and persistent net losses of child vaccinations that continue to number over 20,000 for BCG, 14,000 for first pentavalent, and 3,200 for measles, although, again, these are point estimates, and confidence intervals around these forecasts as of December 2016 contain zero. In the short term, significant efforts should be launched across EVD-affected countries to ensure that coverage of all essential child vaccinations reaches the minimum levels necessary to maintain herd immunity. In the longer term, system strengthening efforts are needed to ensure that routine immunization activities can maintain high levels of coverage in the post-EVD environment across West Africa. Our finding that it took only 7 months (June–December 2014) to accumulate more than 12,000 lost BCG vaccinations, 9,000 lost measles vaccinations, and 12,000 lost first pentavalent vaccinations suggests that during future public health emergencies, significant and rapid efforts must be made to maintain the delivery of essential child vaccinations in public-sector primary care even during the most challenging times.

Our study has several important limitations. First, due to inconsistent indicators in the national RHIS, we were unable to include any HIV or tuberculosis data in the present analyses. Second, Montserrado County was excluded from this study, which limits generalizability and means that the calculated system losses due to EVD are likely an underestimate. Third, these analyses relied on aggregate RHIS data that have not undergone systematic or large-scale data quality audits, and thus we cannot confirm the consistency, reliability, or validity of these data. We found some small increases in the amount of missing data during the first 4 months of the EVD epidemic compared to the 5 months just previous to the EVD epidemic—however, there were also some indicators that had no changes in missingness. Without knowledge of data quality over time pre- and post-EVD, it is likely that the health system disruption during the EVD outbreak may have caused some bias in the effect sizes presented in this study. Future studies could attempt to understand how the EVD outbreak affected RHIS data quality to inform use of RHIS data to track future epidemics or health system emergencies. Fourth, our assessment of system outputs did not quantify potential changes in the quality of care provided before, during, and after the EVD outbreak, including disruptions in essential supplies, medicines, reagents, and staff. Last, given the scope of this paper, we were unable to analyze facility- or county-level factors that may have influenced changes in system outputs. This is an area that could be examined in future studies.

Despite these limitations, our study has some notable strengths. We analyzed RHIS data at the facility level, using robust statistical methods and flexible time trends to accurately estimate the functional forms of reductions in health services due to the EVD outbreak. In addition, we used 7 years of data across a census of all public-sector MoH facilities in Liberia outside Montserrado County, resulting in a database of over 30,000 facility-month observations. We analyzed multiple nonoverlapping essential primary healthcare indicators, all of which had relatively low rates of missing data and limited changes in missing data patterns over the 7-year period. These analyses serve as an example of the value of RHIS data for rapidly and effectively tracking health system performance in low- and middle-income countries [38].

In summary, there were rapid and significant disruptions to public-sector primary healthcare delivery during and immediately after the EVD outbreak in Liberia, with system losses of 36% to 67% of pre-EVD levels. The Liberian health system showed early signs of resilience during and after the EVD outbreak, but not before accruing large gaps in essential primary care service delivery. As of November 2016, all indicators tracked have recovered to pre-EVD levels, although large net losses likely exist for essential childhood vaccinations, maternal health services, and primary care visits. Due to the disruption of malaria case management during the EVD outbreak, malaria cases have rebounded from steady decreases pre-EVD to an increasing trend and 50% higher caseloads in December 2016 compared to December 2013. More than 20 months after Liberia was declared free of EVD, persistent gaps in public-sector health service delivery continue due to lingering effects from the EVD outbreak. During future public health emergencies, funding should be explicitly allocated to maintain public-sector primary healthcare delivery and to promote recovery of systems functioning post-emergency. RHIS data should be considered an essential tool to track future epidemics and health system emergencies in real time and should receive more funding, attention, and use. Sustained investments in public-sector health system strengthening are needed across EVD-affected countries to close gaps in primary care that occurred during the EVD outbreak, and to build resilient primary healthcare systems capable of mitigating collateral effects of the next emerging epidemic.

## Supporting information

S1 Fig Political map of the counties of Liberia. Source: http://www.un.org/Depts/Cartographic/map/profile/liberia.pdf.
(TIF)

S1 Text Comprehensive Stata do file for data cleaning and analyses. (DOCX)

## Abbreviations

ACT: artemisinin-based combination therapy
ANC: antenatal care
ARI: acute respiratory infection
BCG: bacille Calmette–Guérin
DHIS 2: District Health Information Software 2
EVD: Ebola virus disease
MCH: maternal and child health
MoH: Ministry of Health
PNC: postnatal care visits
RHIS: routine health information system
