# Supplementary material for: The 2014–2015 Ebola virus disease outbreak and primary healthcare delivery in Liberia: Time-series analyses for 2010–2016
Source: PLoS Med. 2018 Feb 20;15(2):e1002508. doi: 10.1371/journal.pmed.1002508 (PMC5819774; doi:10.1371/journal.pmed.1002508)
Supplement: S1 Text — (DOCX) [file pmed.1002508.s002.docx]

*******************************************************

* DHIS-2 Liberia Analyses

* Programmer: Brad Wagenaar

*******************************************************

use "FILEPATH HERE.dta", clear

set more off

*************************************************************

*** Data cleaning and variable creation commands

*************************************************************

egen cluster = group(organisationunitid)

egen countycluster = group(county)

sort cluster periodid

by cluster: gen month=_n

drop if periodid==.

*removing first 2 years of data before 2010

drop if month<25

*generate yearly spline

mkspline time1 49 time2 61 time3 73 time4 85 time5 97 time6 109 time7 = month

*generate year indicators

gen y2007=0

replace y2007=1 if month<13

gen y2008=0

replace y2008=1 if month>12 & month<25

gen y2009=0

replace y2009=1 if month>24 & month<37

gen y2010=0

replace y2010=1 if month>36 & month<49

gen y2011=0

replace y2011=1 if month>48 & month<61

gen y2012=0

replace y2012=1 if month>60 & month<73

gen y2013=0

replace y2013=1 if month>72 & month<85

gen y2014=0

replace y2014=1 if month>84 & month<97

gen y2015=0

replace y2015=1 if month>96 & month<109

gen y2016=0

replace y2016=1 if month>108

*Ebola indicator (May 2014 - April 2015)

gen ebola=0

replace ebola=1 if month>89

*Time after Ebola (first 4 months)

gen timeafterebola1=0

by cluster: replace timeafterebola1=(_n-65) if month>89

*Time after Ebola (second 6 months)

gen timeafterebola2=0

by cluster: replace timeafterebola2=(_n-69) if month>93

*Generate no Ebola Indicator

gen postebola=0

replace postebola=1 if month>100

*Time after no Ebola

gen timeafternoebola=0

by cluster: replace timeafternoebola=(_n-76) if month>100

*Dropping Montserrado county clinics

drop if county=="Montserrado"

*Generate month indicators

gen Jan=0

gen Feb=0

gen Mar=0

gen Apr=0

gen May=0

gen Jun=0

gen July=0

gen Aug=0

gen Sep=0

gen Oct=0

gen Nov=0

gen Dec=0

replace Jan=1 if month==25 | month==37 | month==49 | month==61 | month==73 | month==85 | month==97 | month==109

replace Feb=1 if month==26 | month==38 | month==50 | month==62 | month==74 | month==86 | month==98 | month==110

replace Mar=1 if month==27 | month==39 | month==51 | month==63 | month==75 | month==87 | month==99 | month==111

replace Apr=1 if month==28 | month==40 | month==52 | month==64 | month==76 | month==88 | month==100 | month==112

replace May=1 if month==29 | month==41 | month==53 | month==65 | month==77 | month==89 | month==101 | month==113

replace Jun=1 if month==30 | month==42 | month==54 | month==66 | month==78 | month==90 | month==102 | month==114

replace July=1 if month==31 | month==43 | month==55 | month==67 | month==79 | month==91 | month==103 | month==115

replace Aug=1 if month==32 | month==44 | month==56 | month==68 | month==80 | month==92 | month==104 | month==116

replace Sep=1 if month==33 | month==45 | month==57 | month==69 | month==81 | month==93 | month==105 | month==117

replace Oct=1 if month==34 | month==46 | month==58 | month==70 | month==82 | month==94 | month==106 | month==118

replace Nov=1 if month==35 | month==47 | month==59 | month==71 | month==83 | month==95 | month==107 | month==119

replace Dec=1 if month==36 | month==48 | month==60 | month==72 | month==84 | month==96 | month==108 | month==120

*************************************************

*DATA CLEANING AND OUTLIER IDENTIFICATION

*DATA CLEANING BCG*

sort cluster

egen bcgnmis=rmiss(bcgdosesgiven)

by cluster: egen bcgmiss=sum(bcgnmis)

generate allmisbcg=0

replace allmisbcg=1 if bcgmiss>84

by cluster: egen meanbcggiven=mean(bcgdosesgiven)

gen bcgexclude=0

replace bcgexclude=1 if meanbcggiven==0

generate bcgdosesoutlier=.

generate bcgCIlow=.

generate bcgCIhigh=.

gen bcgstandarderror_ = .

gen bcgsmooth_grid_ = .

gen bcgsmoothed_values_ = .

sort cluster

levelsof cluster if allmisbcg==0, local(facility)

foreach l in `facility' {

qui {

quietly lpoly bcgdosesgiven month if cluster==`l' & allmisbcg==0, se(bcgstandarderror) generate(bcgsmooth_grid bcgsmoothed_values) kernel(epan2) at(month) nograph

replace bcgCIlow=(bcgsmoothed_values-(invnormal(.999999999999999)*bcgstandarderror)) if cluster==`l' & allmisbcg==0

replace bcgCIhigh=(bcgsmoothed_values+(invnormal(.999999999999999)*bcgstandarderror)) if cluster==`l' & allmisbcg==0

replace bcgdosesoutlier=1 if bcgdosesgiven>bcgCIhigh & cluster==`l' & allmisbcg==0 & bcgdosesgiven!=.

replace bcgdosesoutlier=1 if bcgdosesgiven<bcgCIlow & cluster==`l' & allmisbcg==0 & bcgdosesgiven!=.

replace bcgstandarderror_ = bcgstandarderror if cluster==`l'

replace bcgsmooth_grid_ = bcgsmooth_grid if cluster==`l'

replace bcgsmoothed_values_ = bcgsmoothed_values if cluster==`l'

**

drop bcgstandarderror bcgsmooth_grid bcgsmoothed_values

}

}

*DATA CLEANING Measles*

egen measlesnmis=rmiss(measlesdosesgiven)

by cluster: egen measlesmiss=sum(measlesnmis)

generate allmismeasles=0

replace allmismeasles=1 if measlesmiss>84

by cluster: egen meanmeaslesgiven=mean(measlesdosesgiven)

gen measlesexclude=0

replace measlesexclude=1 if meanmeaslesgiven==0

generate measlesdosesoutlier=.

generate measlesCIlow=.

generate measlesCIhigh=.

gen measlesstandarderror_ = .

gen measlessmooth_grid_ = .

gen measlessmoothed_values_ = .

sort cluster

levelsof cluster if allmismeasles==0, local(facility)

foreach l in `facility' {

qui {

quietly lpoly measlesdosesgiven month if cluster==`l' & allmismeasles==0, se(measlesstandarderror) generate(measlessmooth_grid measlessmoothed_values) kernel(epan2) at(month) nograph

replace measlesCIlow=(measlessmoothed_values-(invnormal(.999999999999999)*measlesstandarderror)) if cluster==`l' & allmismeasles==0

replace measlesCIhigh=(measlessmoothed_values+(invnormal(.999999999999999)*measlesstandarderror)) if cluster==`l' & allmismeasles==0

replace measlesdosesoutlier=1 if measlesdosesgiven>measlesCIhigh & cluster==`l' & allmismeasles==0 & measlesdosesgiven!=.

replace measlesdosesoutlier=1 if measlesdosesgiven<measlesCIlow & cluster==`l' & allmismeasles==0 & measlesdosesgiven!=.

replace measlesstandarderror_ = measlesstandarderror if cluster==`l'

replace measlessmooth_grid_ = measlessmooth_grid if cluster==`l'

replace measlessmoothed_values_ = measlessmoothed_values if cluster==`l'

**

drop measlesstandarderror measlessmooth_grid measlessmoothed_values

}

}

*DATA CLEANING Headcount*

egen headnmis=rmiss(headcount)

by cluster: egen headmiss=sum(headnmis)

generate allmishead=0

replace allmishead=1 if headmiss>84

by cluster: egen meanheadgiven=mean(headcount)

gen headexclude=0

replace headexclude=1 if meanheadgiven==0

generate headoutlier=.

generate headCIlow=.

generate headCIhigh=.

gen headstandarderror_ = .

gen headsmooth_grid_ = .

gen headsmoothed_values_ = .

sort cluster

levelsof cluster if allmishead==0, local(facility)

foreach l in `facility' {

qui {

quietly lpoly headcount month if cluster==`l' & allmishead==0, se(headstandarderror) generate(headsmooth_grid headsmoothed_values) kernel(epan2) at(month) nograph

replace headCIlow=(headsmoothed_values-(invnormal(.999999999999999)*headstandarderror)) if cluster==`l' & allmishead==0

replace headCIhigh=(headsmoothed_values+(invnormal(.999999999999999)*headstandarderror)) if cluster==`l' & allmishead==0

replace headoutlier=1 if headcount>headCIhigh & cluster==`l' & allmishead==0 & headcount!=.

replace headoutlier=1 if headcount<headCIlow & cluster==`l' & allmishead==0 & headcount!=.

replace headstandarderror_ = headstandarderror if cluster==`l'

replace headsmooth_grid_ = headsmooth_grid if cluster==`l'

replace headsmoothed_values_ = headsmoothed_values if cluster==`l'

**

drop headstandarderror headsmooth_grid headsmoothed_values

}

}

*DATA CLEANING delivery *

egen deliverynmis=rmiss(skilleddelfacility)

by cluster: egen deliverymiss=sum(deliverynmis)

generate allmisdelivery=0

replace allmisdelivery=1 if deliverymiss>84

by cluster: egen meandeliverygiven=mean(skilleddelfacility)

gen deliveryexclude=0

replace deliveryexclude=1 if meandeliverygiven==0

generate deliverydosesoutlier=.

generate deliveryCIlow=.

generate deliveryCIhigh=.

gen deliverystandarderror_ = .

gen deliverysmooth_grid_ = .

gen deliverysmoothed_values_ = .

sort cluster

levelsof cluster if allmisdelivery==0, local(facility)

foreach l in `facility' {

qui {

quietly lpoly skilleddelfacility month if cluster==`l' & allmisdelivery==0, se(deliverystandarderror) generate(deliverysmooth_grid deliverysmoothed_values) kernel(epan2) at(month) nograph

replace deliveryCIlow=(deliverysmoothed_values-(invnormal(.999999999999999)*deliverystandarderror)) if cluster==`l' & allmisdelivery==0

replace deliveryCIhigh=(deliverysmoothed_values+(invnormal(.999999999999999)*deliverystandarderror)) if cluster==`l' & allmisdelivery==0

replace deliverydosesoutlier=1 if skilleddelfacility>deliveryCIhigh & cluster==`l' & allmisdelivery==0 & skilleddelfacility!=.

replace deliverydosesoutlier=1 if skilleddelfacility<deliveryCIlow & cluster==`l' & allmisdelivery==0 & skilleddelfacility!=.

replace deliverystandarderror_ = deliverystandarderror if cluster==`l'

replace deliverysmooth_grid_ = deliverysmooth_grid if cluster==`l'

replace deliverysmoothed_values_ = deliverysmoothed_values if cluster==`l'

**

drop deliverystandarderror deliverysmooth_grid deliverysmoothed_values

}

}

*DATA CLEANING acttreatment*

egen actnmis=rmiss(acttreatment)

by cluster: egen actmiss=sum(actnmis)

generate allmisact=0

replace allmisact=1 if actmiss>84

by cluster: egen meanactgiven=mean(acttreatment)

gen actexclude=0

replace actexclude=1 if meanactgiven==0

generate actdosesoutlier=.

generate actCIlow=.

generate actCIhigh=.

gen actstandarderror_ = .

gen actsmooth_grid_ = .

gen actsmoothed_values_ = .

sort cluster

levelsof cluster if allmisact==0, local(facility)

foreach l in `facility' {

qui {

quietly lpoly acttreatment month if cluster==`l' & allmisact==0, se(actstandarderror) generate(actsmooth_grid actsmoothed_values) kernel(epan2) at(month) nograph

replace actCIlow=(actsmoothed_values-(invnormal(.999999999999999)*actstandarderror)) if cluster==`l' & allmisact==0

replace actCIhigh=(actsmoothed_values+(invnormal(.999999999999999)*actstandarderror)) if cluster==`l' & allmisact==0

replace actdosesoutlier=1 if acttreatment>actCIhigh & cluster==`l' & allmisact==0 & acttreatment!=.

replace actdosesoutlier=1 if acttreatment<actCIlow & cluster==`l' & allmisact==0 & acttreatment!=.

replace actstandarderror_ = actstandarderror if cluster==`l'

replace actsmooth_grid_ = actsmooth_grid if cluster==`l'

replace actsmoothed_values_ = actsmoothed_values if cluster==`l'

**

drop actstandarderror actsmooth_grid actsmoothed_values

}

}

*DATA CLEANING pentavalent1given*

egen pentanmis=rmiss(pentavalent1given)

by cluster: egen pentamiss=sum(pentanmis)

generate allmispenta=0

replace allmispenta=1 if pentamiss>84

by cluster: egen meanpentagiven=mean(pentavalent1given)

gen pentaexclude=0

replace pentaexclude=1 if meanpentagiven==0

generate pentadosesoutlier=.

generate pentaCIlow=.

generate pentaCIhigh=.

gen pentastandarderror_ = .

gen pentasmooth_grid_ = .

gen pentasmoothed_values_ = .

sort cluster

levelsof cluster if allmispenta==0, local(facility)

foreach l in `facility' {

qui {

quietly lpoly pentavalent1given month if cluster==`l' & allmispenta==0, se(pentastandarderror) generate(pentasmooth_grid pentasmoothed_values) kernel(epan2) at(month) nograph

replace pentaCIlow=(pentasmoothed_values-(invnormal(.999999999999999)*pentastandarderror)) if cluster==`l' & allmispenta==0

replace pentaCIhigh=(pentasmoothed_values+(invnormal(.999999999999999)*pentastandarderror)) if cluster==`l' & allmispenta==0

replace pentadosesoutlier=1 if pentavalent1given>pentaCIhigh & cluster==`l' & allmispenta==0 & pentavalent1given!=.

replace pentadosesoutlier=1 if pentavalent1given<pentaCIlow & cluster==`l' & allmispenta==0 & pentavalent1given!=.

replace pentastandarderror_ = pentastandarderror if cluster==`l'

replace pentasmooth_grid_ = pentasmooth_grid if cluster==`l'

replace pentasmoothed_values_ = pentasmoothed_values if cluster==`l'

**

drop pentastandarderror pentasmooth_grid pentasmoothed_values

}

}

*DATA CLEANING ari*

egen respiratorynmis=rmiss(ari)

by cluster: egen respiratorymiss=sum(respiratorynmis)

generate allmisrespiratory=0

replace allmisrespiratory=1 if respiratorymiss>84

by cluster: egen meanrespiratorygiven=mean(ari)

gen respiratoryexclude=0

replace respiratoryexclude=1 if meanrespiratorygiven==0

generate respiratorydosesoutlier=.

generate respiratoryCIlow=.

generate respiratoryCIhigh=.

gen respiratorystandarderror_ = .

gen respiratorysmooth_grid_ = .

gen respiratorysmoothed_values_ = .

sort cluster

levelsof cluster if allmisrespiratory==0, local(facility)

foreach l in `facility' {

qui {

quietly lpoly ari month if cluster==`l' & allmisrespiratory==0, se(respiratorystandarderror) generate(respiratorysmooth_grid respiratorysmoothed_values) kernel(epan2) at(month) nograph

replace respiratoryCIlow=(respiratorysmoothed_values-(invnormal(.999999999999999)*respiratorystandarderror)) if cluster==`l' & allmisrespiratory==0

replace respiratoryCIhigh=(respiratorysmoothed_values+(invnormal(.999999999999999)*respiratorystandarderror)) if cluster==`l' & allmisrespiratory==0

replace respiratorydosesoutlier=1 if ari>respiratoryCIhigh & cluster==`l' & allmisrespiratory==0 & ari!=.

replace respiratorydosesoutlier=1 if ari<respiratoryCIlow & cluster==`l' & allmisrespiratory==0 & ari!=.

replace respiratorystandarderror_ = respiratorystandarderror if cluster==`l'

replace respiratorysmooth_grid_ = respiratorysmooth_grid if cluster==`l'

replace respiratorysmoothed_values_ = respiratorysmoothed_values if cluster==`l'

**

drop respiratorystandarderror respiratorysmooth_grid respiratorysmoothed_values

}

}

*DATA CLEANING 1stancvisit*

egen antenatalnmis=rmiss(stancvisit)

by cluster: egen antenatalmiss=sum(antenatalnmis)

generate allmisantenatal=0

replace allmisantenatal=1 if antenatalmiss>84

by cluster: egen meanantenatalgiven=mean(stancvisit)

gen antenatalexclude=0

replace antenatalexclude=1 if meanantenatalgiven==0

generate antenataldosesoutlier=.

generate antenatalCIlow=.

generate antenatalCIhigh=.

gen antenatalstandarderror_ = .

gen antenatalsmooth_grid_ = .

gen antenatalsmoothed_values_ = .

sort cluster

levelsof cluster if allmisantenatal==0, local(facility)

foreach l in `facility' {

qui {

quietly lpoly stancvisit month if cluster==`l' & allmisantenatal==0, se(antenatalstandarderror) generate(antenatalsmooth_grid antenatalsmoothed_values) kernel(epan2) at(month) nograph

replace antenatalCIlow=(antenatalsmoothed_values-(invnormal(.999999999999999)*antenatalstandarderror)) if cluster==`l' & allmisantenatal==0

replace antenatalCIhigh=(antenatalsmoothed_values+(invnormal(.999999999999999)*antenatalstandarderror)) if cluster==`l' & allmisantenatal==0

replace antenataldosesoutlier=1 if stancvisit>antenatalCIhigh & cluster==`l' & allmisantenatal==0 & stancvisit!=.

replace antenataldosesoutlier=1 if stancvisit<antenatalCIlow & cluster==`l' & allmisantenatal==0 & stancvisit!=.

replace antenatalstandarderror_ = antenatalstandarderror if cluster==`l'

replace antenatalsmooth_grid_ = antenatalsmooth_grid if cluster==`l'

replace antenatalsmoothed_values_ = antenatalsmoothed_values if cluster==`l'

**

drop antenatalstandarderror antenatalsmooth_grid antenatalsmoothed_values

}

}

*DATA CLEANING depo provera*

egen deponmis=rmiss(depoprovera)

by cluster: egen depomiss=sum(deponmis)

generate allmisdepo=0

replace allmisdepo=1 if depomiss>84

by cluster: egen meandepogiven=mean(depoprovera)

gen depoexclude=0

replace depoexclude=1 if meandepogiven==0

generate depodosesoutlier=.

generate depoCIlow=.

generate depoCIhigh=.

gen depostandarderror_ = .

gen deposmooth_grid_ = .

gen deposmoothed_values_ = .

sort cluster

levelsof cluster if allmisdepo==0, local(facility)

foreach l in `facility' {

qui {

quietly lpoly depoprovera month if cluster==`l' & allmisdepo==0, se(depostandarderror) generate(deposmooth_grid deposmoothed_values) kernel(epan2) at(month) nograph

replace depoCIlow=(deposmoothed_values-(invnormal(.999999999999999)*depostandarderror)) if cluster==`l' & allmisdepo==0

replace depoCIhigh=(deposmoothed_values+(invnormal(.999999999999999)*depostandarderror)) if cluster==`l' & allmisdepo==0

replace depodosesoutlier=1 if depoprovera>depoCIhigh & cluster==`l' & allmisdepo==0 & depoprovera!=.

replace depodosesoutlier=1 if depoprovera<depoCIlow & cluster==`l' & allmisdepo==0 & depoprovera!=.

replace depostandarderror_ = depostandarderror if cluster==`l'

replace deposmooth_grid_ = deposmooth_grid if cluster==`l'

replace deposmoothed_values_ = deposmoothed_values if cluster==`l'

**

drop depostandarderror deposmooth_grid deposmoothed_values

}

}

*DATA CLEANING pncwithin6weeks*

egen pncnmis=rmiss(pncwithin6weeks)

by cluster: egen pncmiss=sum(pncnmis)

generate allmispnc=0

replace allmispnc=1 if pncmiss>84

by cluster: egen meanpncgiven=mean(pncwithin6weeks)

gen pncexclude=0

replace pncexclude=1 if meanpncgiven==0

generate pncdosesoutlier=.

generate pncCIlow=.

generate pncCIhigh=.

gen pncstandarderror_ = .

gen pncsmooth_grid_ = .

gen pncsmoothed_values_ = .

sort cluster

levelsof cluster if allmispnc==0, local(facility)

foreach l in `facility' {

qui {

quietly lpoly pncwithin6weeks month if cluster==`l' & allmispnc==0, se(pncstandarderror) generate(pncsmooth_grid pncsmoothed_values) kernel(epan2) at(month) nograph

replace pncCIlow=(pncsmoothed_values-(invnormal(.999999999999999)*pncstandarderror)) if cluster==`l' & allmispnc==0

replace pncCIhigh=(pncsmoothed_values+(invnormal(.999999999999999)*pncstandarderror)) if cluster==`l' & allmispnc==0

replace pncdosesoutlier=1 if pncwithin6weeks>pncCIhigh & cluster==`l' & allmispnc==0 & pncwithin6weeks!=.

replace pncdosesoutlier=1 if pncwithin6weeks<pncCIlow & cluster==`l' & allmispnc==0 & pncwithin6weeks!=.

replace pncstandarderror_ = pncstandarderror if cluster==`l'

replace pncsmooth_grid_ = pncsmooth_grid if cluster==`l'

replace pncsmoothed_values_ = pncsmoothed_values if cluster==`l'

**

drop pncstandarderror pncsmooth_grid pncsmoothed_values

}

}

save "FILE PATH2.dta", replace

**********************************************************************

** DATA ANALYSIS

**********************************************************************

use "FILEPATH2.dta", clear

set more off

cd "GENERAL FILE PATH"

**Including only data of interest (Jan 2010 - Dec 2016)

drop if month<37

*Sorting by month

sort month

*Fix missing population for a few clusters

egen popmiss=rmiss(population)

sort cluster

by cluster: egen popnmiss=sum(popmiss)

drop if popnmiss==84

by cluster: egen meanpop=mean(population)

replace population=meanpop if popnmiss==72

save analysisdataset, replace

*****************************************

**Analysis command BCG

*****************************************

xtmixed bcgdosesgiven time1-time7 population Feb Mar Apr May Jun July Aug Sep Oct Nov Dec timeafterebola1 timeafterebola2 timeafternoebola|| cluster:time1-time7 if bcgexclude==0 & bcgdosesoutlier!=1 & allmisbcg==0, residuals(ar, t(month))

*Predicting fitted values and CIs

predict fittedpoission

predict fitted_mean, xb

predict fitted_mean_se, stdp

generate upperci = fittedpoission + 1.96*fitted_mean_se

generate lowerci = fittedpoission - 1.96*fitted_mean_se

sort month

by month: egen BCGtest = mean(fitted_mean)

by month: egen BCGtestlowerci = mean(lowerci)

by month: egen BCGtestupperci = mean(upperci)

by month: egen BCGaverage = mean(bcgdosesgiven)

by month: egen fittedavg = mean(fittedpoission)

by month: egen meanBCG = mean(bcgdosesgiven) if bcgexclude==0 & bcgdosesoutlier!=1 & allmisbcg==0

est store bcg_full

est save bcg_full_file, replace

*BCG graph over time

twoway (line fittedavg month, xlabel(#7) lcolor(black) legend(off) ytitle("Mean Number of BCG Doses") ///

xtitle("Date") graphregion(fcolor(white)) plotregion(style(none)) ylabel(,nogrid angle(horizontal)) msize(small) ///

ylabel(0(5)35) xlabel(37 "Jan 2010" 49 "Jan 2011" 61 "Jan 2012" 73 "Jan 2013" 85 "Jan 2014" 97 "Jan 2015" 109 "Jan 2016")) (line BCGtestupperci month, lcolor(gray) lpattern(dash)) ///

(line BCGtestlowerci month, lcolor(gray) lpattern(dash)) ///

(scatter meanBCG month, xline(89) xline(100) msize(medium) lcolor(blue) mcolor(blue))

drop fittedpoission fitted_mean fitted_mean_se upperci lowerci BCGtest BCGtestlowerci BCGtestupperci fittedavg meanBCG BCGaverage

*Reduced model before ebola for forecasts

xtmixed bcgdosesgiven time1-time4 population Feb Mar Apr May Jun July Aug Sep Oct Nov Dec || cluster:time1-time4 if bcgexclude==0 & bcgdosesoutlier!=1 & allmisbcg==0 & month<=89, residuals(ar, t(month))

est store bcg_reduc

est save bcg_reduc_file

*****************************************

**Analysis command Head Count

*****************************************

xtmixed headcount time1-time7 population Feb Mar Apr May Jun July Aug Sep Oct Nov Dec timeafterebola1 timeafterebola2 timeafternoebola|| cluster:time1-time7 if headexclude==0 & headoutlier!=1 & allmishead==0 , residuals(ar, t(month))

*Predicting fitted values and CIs

predict fittedpoission

predict fitted_mean, xb

predict fitted_mean_se, stdp

generate upperci = fittedpoission + 1.96*fitted_mean_se

generate lowerci = fittedpoission - 1.96*fitted_mean_se

sort month

by month: egen BCGtest = mean(fitted_mean)

by month: egen BCGtestlowerci = mean(lowerci)

by month: egen BCGtestupperci = mean(upperci)

by month: egen BCGaverage = mean(bcgdosesgiven)

by month: egen fittedavg = mean(fittedpoission)

by month: egen meanBCG = mean(headcount) if headexclude==0 & headoutlier!=1 & allmishead==0

est store headcount_full

est save headcount_full_file, replace

*Head count graph over time

twoway (line fittedavg month, xlabel(#7) lcolor(black) legend(off) ytitle("Mean Number of Clinic Visits") ///

xtitle("Date") graphregion(fcolor(white)) plotregion(style(none)) ylabel(,nogrid angle(horizontal)) msize(small) ///

ylabel(0(100)1000) xlabel(37 "Jan 2010" 49 "Jan 2011" 61 "Jan 2012" 73 "Jan 2013" 85 "Jan 2014" 97 "Jan 2015" 109 "Jan 2016")) (line BCGtestupperci month, lcolor(gray) lpattern(dash)) ///

(line BCGtestlowerci month, lcolor(gray) lpattern(dash)) ///

(scatter meanBCG month, xline(89) xline(100) msize(medium) lcolor(blue) mcolor(blue))

drop fittedpoission fitted_mean fitted_mean_se upperci lowerci BCGtest BCGtestlowerci BCGtestupperci fittedavg meanBCG BCGaverage

*Reduced model before ebola for forecasts

xtmixed headcount time1-time4 population Feb Mar Apr May Jun July Aug Sep Oct Nov Dec || cluster:time1-time4 if headexclude==0 & headoutlier!=1 & allmishead==0 & month<=89, residuals(ar, t(month))

est store headcount_reduc

est save headcount_reduc

*****************************************

**Analysis command Measles

*****************************************

xtmixed measlesdosesgiven time1-time7 population Feb Mar Apr May Jun July Aug Sep Oct Nov Dec timeafterebola1 timeafterebola2 timeafternoebola|| cluster:time1-time7 if measlesexclude==0 & allmismeasles==0, residuals(ar, t(month))

*Predicting fitted values and CIs

predict fittedpoission

predict fitted_mean, xb

predict fitted_mean_se, stdp

generate upperci = fittedpoission + 1.96*fitted_mean_se

generate lowerci = fittedpoission - 1.96*fitted_mean_se

sort month

by month: egen BCGtest = mean(fitted_mean)

by month: egen BCGtestlowerci = mean(lowerci)

by month: egen BCGtestupperci = mean(upperci)

by month: egen BCGaverage = mean(bcgdosesgiven)

by month: egen fittedavg = mean(fittedpoission)

by month: egen meanBCG = mean(measlesdosesgiven) if measlesexclude==0 & allmismeasles==0

est store measles_full

est save measles_full_file, replace

*Measles graph over time

twoway (line fittedavg month, xlabel(#7) lcolor(black) legend(off) ytitle("Mean Number of Measles Doses") ///

xtitle("Date") graphregion(fcolor(white)) plotregion(style(none)) ylabel(,nogrid angle(horizontal)) msize(small) ///

ylabel(0(5)35) xlabel(37 "Jan 2010" 49 "Jan 2011" 61 "Jan 2012" 73 "Jan 2013" 85 "Jan 2014" 97 "Jan 2015" 109 "Jan 2016")) (line BCGtestupperci month, lcolor(gray) lpattern(dash)) ///

(line BCGtestlowerci month, lcolor(gray) lpattern(dash)) ///

(scatter meanBCG month, xline(89) xline(100) msize(medium) lcolor(blue) mcolor(blue))

sort cluster month

drop fittedpoission fitted_mean fitted_mean_se upperci lowerci BCGtest BCGtestlowerci BCGtestupperci fittedavg meanBCG BCGaverage

*Reduced model before ebola for forecasts

xtmixed measlesdosesgiven time1-time4 population Feb Mar Apr May Jun July Aug Sep Oct Nov Dec || cluster:time1-time4 if measlesexclude==0 & allmismeasles==0 & month<=89, residuals(ar, t(month))

est store measles_reduc

est save measles_reduc_file, replace

*****************************************

**Analysis command institutional births

*****************************************

xtmixed skilleddelfacility time1-time7 population Feb Mar Apr May Jun July Aug Sep Oct Nov Dec timeafterebola1 timeafterebola2 timeafternoebola|| cluster:time1-time7 if deliveryexclude==0 & deliverydosesoutlier!=1 & allmisdelivery==0, residuals(ar, t(month))

*Predicting fitted values and CIs

predict fittedpoission

predict fitted_mean, xb

predict fitted_mean_se, stdp

generate upperci = fittedpoission + 1.96*fitted_mean_se

generate lowerci = fittedpoission - 1.96*fitted_mean_se

sort month

by month: egen BCGtest = mean(fitted_mean)

by month: egen BCGtestlowerci = mean(lowerci)

by month: egen BCGtestupperci = mean(upperci)

by month: egen BCGaverage = mean(bcgdosesgiven)

by month: egen fittedavg = mean(fittedpoission)

by month: egen meanBCG = mean(skilleddelfacility) if deliveryexclude==0 & deliverydosesoutlier!=1 & allmisdelivery==0

est store delivery_full

est save delivery_full_file, replace

*Institutional births graph over time

twoway (line fittedavg month, xlabel(#7) lcolor(black) legend(off) ytitle("Mean Institutional Births") ///

xtitle("Date") graphregion(fcolor(white)) plotregion(style(none)) ylabel(,nogrid angle(horizontal)) msize(small) ///

ylabel(0(5)20) xlabel(37 "Jan 2010" 49 "Jan 2011" 61 "Jan 2012" 73 "Jan 2013" 85 "Jan 2014" 97 "Jan 2015" 109 "Jan 2016")) (line BCGtestupperci month, lcolor(gray) lpattern(dash)) ///

(line BCGtestlowerci month, lcolor(gray) lpattern(dash)) ///

(scatter meanBCG month, xline(89) xline(100) msize(medium) lcolor(blue) mcolor(blue))

drop fittedpoission fitted_mean fitted_mean_se upperci lowerci BCGtest BCGtestlowerci BCGtestupperci fittedavg meanBCG BCGaverage

*Reduced model before ebola for forecasts

xtmixed skilleddelfacility time1-time4 population Feb Mar Apr May Jun July Aug Sep Oct Nov Dec || cluster:time1-time4 if deliveryexclude==0 & deliverydosesoutlier!=1 & allmisdelivery==0 & month<=89, residuals(ar, t(month))

est store delivery_reduc

est save delivery_reduc_file, replace

*****************************************

**Analysis command ACT measles treatment

*****************************************

xtmixed acttreatment time1-time7 population Feb Mar Apr May Jun July Aug Sep Oct Nov Dec timeafterebola1 timeafterebola2 timeafternoebola|| cluster:time1-time7 if actexclude==0 & actdosesoutlier!=1 & allmisact==0, residuals(ar, t(month))

*Predicting fitted values and CIs

predict fittedpoission

predict fitted_mean, xb

predict fitted_mean_se, stdp

generate upperci = fittedpoission + 1.96*fitted_mean_se

generate lowerci = fittedpoission - 1.96*fitted_mean_se

sort month

by month: egen BCGtest = mean(fitted_mean)

by month: egen BCGtestlowerci = mean(lowerci)

by month: egen BCGtestupperci = mean(upperci)

by month: egen BCGaverage = mean(bcgdosesgiven)

by month: egen fittedavg = mean(fittedpoission)

by month: egen meanBCG = mean(acttreatment) if actexclude==0 & actdosesoutlier!=1 & allmisact==0

est store ACT_full

est save ACT_full_file, replace

*ACT graph over time

twoway (line fittedavg month, xlabel(#7) lcolor(black) legend(off) ytitle("Mean Number of ACT Treatments for Malaria") ///

xtitle("Date") graphregion(fcolor(white)) plotregion(style(none)) ylabel(,nogrid angle(horizontal)) msize(small) ///

ylabel(0(50)300) xlabel(37 "Jan 2010" 49 "Jan 2011" 61 "Jan 2012" 73 "Jan 2013" 85 "Jan 2014" 97 "Jan 2015" 109 "Jan 2016")) (line BCGtestupperci month, lcolor(gray) lpattern(dash)) ///

(line BCGtestlowerci month, lcolor(gray) lpattern(dash)) ///

(scatter meanBCG month, xline(89) xline(100) msize(medium) lcolor(blue) mcolor(blue))

drop fittedpoission fitted_mean fitted_mean_se upperci lowerci BCGtest BCGtestlowerci BCGtestupperci fittedavg meanBCG BCGaverage

*Reduced model before ebola for forecasts

xtmixed acttreatment time1-time4 population Feb Mar Apr May Jun July Aug Sep Oct Nov Dec || cluster:time1-time4 if actexclude==0 & actdosesoutlier!=1 & allmisact==0 & month<=89, residuals(ar, t(month))

est store ACT_reduc

est save ACT_reduc_file

*****************************************

**Analysis command 1st pentavalent vaccinations

*****************************************

xtmixed pentavalent1given time1-time7 population Feb Mar Apr May Jun July Aug Sep Oct Nov Dec timeafterebola1 timeafterebola2 timeafternoebola|| cluster:time1-time7 if pentaexclude==0 & pentadosesoutlier!=1 & allmispenta==0, residuals(ar, t(month))

*Predicting fitted values and CIs

predict fittedpoission

predict fitted_mean, xb

predict fitted_mean_se, stdp

generate upperci = fittedpoission + 1.96*fitted_mean_se

generate lowerci = fittedpoission - 1.96*fitted_mean_se

sort month

by month: egen BCGtest = mean(fitted_mean)

by month: egen BCGtestlowerci = mean(lowerci)

by month: egen BCGtestupperci = mean(upperci)

by month: egen BCGaverage = mean(bcgdosesgiven)

by month: egen fittedavg = mean(fittedpoission)

by month: egen meanBCG = mean(pentavalent1given) if pentaexclude==0 & pentadosesoutlier!=1 & allmispenta==0

est store penta_full

est save penta_full_file, replace

*1st penta graph over time

twoway (line fittedavg month, xlabel(#7) lcolor(black) legend(off) ytitle("Mean Pentavalent Doses Given") ///

xtitle("Date") graphregion(fcolor(white)) plotregion(style(none)) ylabel(,nogrid angle(horizontal)) msize(small) ///

ylabel(0(10)40) xlabel(37 "Jan 2010" 49 "Jan 2011" 61 "Jan 2012" 73 "Jan 2013" 85 "Jan 2014" 97 "Jan 2015" 109 "Jan 2016")) (line BCGtestupperci month, lcolor(gray) lpattern(dash)) ///

(line BCGtestlowerci month, lcolor(gray) lpattern(dash)) ///

(scatter meanBCG month, xline(89) xline(100) msize(medium) lcolor(blue) mcolor(blue))

drop fittedpoission fitted_mean fitted_mean_se upperci lowerci BCGtest BCGtestlowerci BCGtestupperci fittedavg meanBCG BCGaverage

*Reduced model before ebola for forecasts

xtmixed pentavalent1given time1-time4 population Feb Mar Apr May Jun July Aug Sep Oct Nov Dec|| cluster:time1-time4 if month<=89 & pentaexclude==0 & pentadosesoutlier!=1 & allmispenta==0, residuals(ar, t(month))

est store penta_reduc

est save penta_reduc_file

*****************************************

**Analysis command ARIs treated

*****************************************

xtmixed ari time3-time7 population Feb Mar Apr May Jun July Aug Sep Oct Nov Dec timeafterebola1 timeafterebola2 timeafternoebola|| cluster:time3-time7 if month>=61 & respiratoryexclude==0 & respiratorydosesoutlier!=1 & allmisrespiratory==0, residuals(ar, t(month))

*Predicting fitted values and CIs

predict fittedpoission

predict fitted_mean, xb

predict fitted_mean_se, stdp

generate upperci = fittedpoission + 1.96*fitted_mean_se

generate lowerci = fittedpoission - 1.96*fitted_mean_se

sort month

by month: egen BCGtest = mean(fitted_mean)

by month: egen BCGtestlowerci = mean(lowerci)

by month: egen BCGtestupperci = mean(upperci)

by month: egen BCGaverage = mean(bcgdosesgiven)

by month: egen fittedavg = mean(fittedpoission)

by month: egen meanBCG = mean(ari) if respiratoryexclude==0 & respiratorydosesoutlier!=1 & allmisrespiratory==0

est store ARI_full

est save ARI_full_file, replace

*ARI graph over time

twoway (line fittedavg month if month>=61, xlabel(#7) lcolor(black) legend(off) ytitle("Mean Number of Acute Respiratory Infections") ///

xtitle("Date") graphregion(fcolor(white)) plotregion(style(none)) ylabel(,nogrid angle(horizontal)) msize(small) ///

ylabel(0(20)150) xlabel(37 "Jan 2010" 49 "Jan 2011" 61 "Jan 2012" 73 "Jan 2013" 85 "Jan 2014" 97 "Jan 2015" 109 "Jan 2016")) (line BCGtestupperci month if month>=61, lcolor(gray) lpattern(dash)) ///

(line BCGtestlowerci month if month>=61, lcolor(gray) lpattern(dash)) ///

(scatter meanBCG month if month>=61, xline(89) xline(100) msize(medium) lcolor(blue) mcolor(blue))

drop fittedpoission fitted_mean fitted_mean_se upperci lowerci BCGtest BCGtestlowerci BCGtestupperci fittedavg meanBCG BCGaverage

*Reduced model before ebola for forecasts

xtmixed ari time3-time4 population Feb Mar Apr May Jun July Aug Sep Oct Nov Dec|| cluster:time3-time4 if month<=89 & respiratoryexclude==0 & respiratorydosesoutlier!=1 & allmisrespiratory==0, residuals(ar, t(month))

est store ARI_reduc

est save ARI_reduc_file

*****************************************

**Analysis command 1st ANC visits

*****************************************

xtmixed stancvisit time1-time7 population Feb Mar Apr May Jun July Aug Sep Oct Nov Dec timeafterebola1 timeafterebola2 timeafternoebola|| cluster:time1-time7 if antenatalexclude==0 & antenataldosesoutlier!=1 & allmisantenatal==0, residuals(ar, t(month))

*Predicting fitted values and CIs

predict fittedpoission

predict fitted_mean, xb

predict fitted_mean_se, stdp

generate upperci = fittedpoission + 1.96*fitted_mean_se

generate lowerci = fittedpoission - 1.96*fitted_mean_se

sort month

by month: egen BCGtest = mean(fitted_mean)

by month: egen BCGtestlowerci = mean(lowerci)

by month: egen BCGtestupperci = mean(upperci)

by month: egen BCGaverage = mean(bcgdosesgiven)

by month: egen fittedavg = mean(fittedpoission)

by month: egen meanBCG = mean(stancvisit) if antenatalexclude==0 & antenataldosesoutlier!=1 & allmisantenatal==0

est store ANC_full

est save ANC_full_file, replace

*1st ANC graph over time

twoway (line fittedavg month, xlabel(#7) lcolor(black) legend(off) ytitle("Mean Number of 1st Antenatal Care Visits") ///

xtitle("Date") graphregion(fcolor(white)) plotregion(style(none)) ylabel(,nogrid angle(horizontal)) msize(small) ///

ylabel(0(10)40) xlabel(37 "Jan 2010" 49 "Jan 2011" 61 "Jan 2012" 73 "Jan 2013" 85 "Jan 2014" 97 "Jan 2015" 109 "Jan 2016")) (line BCGtestupperci month, lcolor(gray) lpattern(dash)) ///

(line BCGtestlowerci month, lcolor(gray) lpattern(dash)) ///

(scatter meanBCG month, xline(89) xline(100) msize(medium) lcolor(blue) mcolor(blue))

drop fittedpoission fitted_mean fitted_mean_se upperci lowerci BCGtest BCGtestlowerci BCGtestupperci fittedavg meanBCG BCGaverage

*Reduced model before ebola for forecasts

xtmixed stancvisit time1-time4 population Feb Mar Apr May Jun July Aug Sep Oct Nov Dec || cluster:time1-time4 if antenatalexclude==0 & antenataldosesoutlier!=1 & allmisantenatal==0 & month<=89, residuals(ar, t(month))

est store ANC_reduc

est save ANC_reduc_file, replace

*****************************************

**Analysis command depo provera

*****************************************

xtmixed depoprovera time1-time7 population Feb Mar Apr May Jun July Aug Sep Oct Nov Dec timeafterebola1 timeafterebola2 timeafternoebola|| cluster:time1-time7 if depoexclude==0 & depodosesoutlier!=1 & allmisdepo==0, residuals(ar, t(month))

*Predicting fitted values and CIs

predict fittedpoission

predict fitted_mean, xb

predict fitted_mean_se, stdp

generate upperci = fittedpoission + 1.96*fitted_mean_se

generate lowerci = fittedpoission - 1.96*fitted_mean_se

sort month

by month: egen BCGtest = mean(fitted_mean)

by month: egen BCGtestlowerci = mean(lowerci)

by month: egen BCGtestupperci = mean(upperci)

by month: egen BCGaverage = mean(bcgdosesgiven)

by month: egen fittedavg = mean(fittedpoission)

by month: egen meanBCG = mean(depoprovera) if depoexclude==0 & depodosesoutlier!=1 & allmisdepo==0

est store depo_full

est save depo_full_file, replace

*Depo graph over time

twoway (line fittedavg month, xlabel(#7) lcolor(black) legend(off) ytitle("Mean Number of Medroxyprogesterone Acetate Doses") ///

xtitle("Date") graphregion(fcolor(white)) plotregion(style(none)) ylabel(,nogrid angle(horizontal)) msize(small) ///

ylabel(0(10)40) xlabel(37 "Jan 2010" 49 "Jan 2011" 61 "Jan 2012" 73 "Jan 2013" 85 "Jan 2014" 97 "Jan 2015" 109 "Jan 2016")) (line BCGtestupperci month, lcolor(gray) lpattern(dash)) ///

(line BCGtestlowerci month, lcolor(gray) lpattern(dash)) ///

(scatter meanBCG month, xline(89) xline(100) msize(medium) lcolor(blue) mcolor(blue))

drop fittedpoission fitted_mean fitted_mean_se upperci lowerci BCGtest BCGtestlowerci BCGtestupperci fittedavg meanBCG BCGaverage

xtmixed depoprovera time1-time7 population Feb Mar Apr May Jun July Aug Sep Oct Nov Dec timeafterebola1 timeafterebola2 timeafternoebola|| cluster:time1-time7 if depoexclude==0 & depodosesoutlier!=1 & allmisdepo==0, residuals(ar, t(month))

est store depo_full

est save depo_full_file, replace

*****************************************

**Analysis command post-natal consultation

*****************************************

xtmixed pncwithin6weeks time3-time7 population Feb Mar Apr May Jun July Aug Sep Oct Nov Dec timeafterebola1 timeafterebola2 timeafternoebola|| cluster:time3-time7 if month>=61 & pncexclude==0 & pncdosesoutlier!=1 & allmispnc==0 , residuals(ar, t(month))

*Predicting fitted values and CIs

predict fittedpoission

predict fitted_mean, xb // just the fixed effects

predict fitted_mean_se, stdp // just the standard errors

generate upperci = fittedpoission + 1.96*fitted_mean_se

generate lowerci = fittedpoission - 1.96*fitted_mean_se

sort month

by month: egen BCGtest = mean(fitted_mean)

by month: egen BCGtestlowerci = mean(lowerci)

by month: egen BCGtestupperci = mean(upperci)

by month: egen BCGaverage = mean(bcgdosesgiven)

by month: egen fittedavg = mean(fittedpoission)

by month: egen meanBCG = mean(pncwithin6weeks) if month>=61 & pncexclude==0 & pncdosesoutlier!=1 & allmispnc==0

est store pnc_full

est save pnc_full_file, replace

*PNC graph over time

twoway (line fittedavg month if month>=61, xlabel(#7) lcolor(black) legend(off) ytitle("Mean Number of Postnatal Care Visits within 6 Weeks") ///

xtitle("Date") graphregion(fcolor(white)) plotregion(style(none)) ylabel(,nogrid angle(horizontal)) msize(small) ///

ylabel(0(5)20) xlabel(37 "Jan 2010" 49 "Jan 2011" 61 "Jan 2012" 73 "Jan 2013" 85 "Jan 2014" 97 "Jan 2015" 109 "Jan 2016")) (line BCGtestupperci month if month>=61, lcolor(gray) lpattern(dash)) ///

(line BCGtestlowerci month if month>=61, lcolor(gray) lpattern(dash)) ///

(scatter meanBCG month if month>=61, xline(89) xline(100) msize(medium) lcolor(blue) mcolor(blue))

drop fittedpoission fitted_mean fitted_mean_se upperci lowerci BCGtest BCGtestlowerci BCGtestupperci fittedavg meanBCG BCGaverage

*************************************************

* COMMANDS FOR TESTING DIFFERENCES OVER TIME

*************************************************

*********************

**** HEAD COUNT *****

*********************

** Jan2010 to January 2014

estimates use headcount_full_file

estimates replay

nlcom (jan2014: _b[_cons] + (_b[population]*(6491.185))+ (_b[time1]*(37))) ///

(jan2010: _b[_cons] + (_b[population]*(7052.435))+ (_b[time1]*(49)) +(_b[time2]*(12))+(_b[time3]*(12)) + _b[time4]*(12)), post

. nlcom _b[jan2010]/_b[jan2014]

_nl: _b[jan2010]/_b[jan2014]

nlcom log(_b[jan2010]) - log(_b[jan2014])

**September 2014 compared to May 2014

estimates use headcount_full_file

estimates replay

nlcom (May2014: _b[_cons] + (_b[population]*(7052.435))+ (_b[time1]*(49)) +(_b[time2]*(12))+(_b[time3]*(12)) + _b[time4]*(12) +(_b[time5]*(4) + (_b[May]*1))) ///

(September2014: _b[_cons] + (_b[population]*(7052.435))+ (_b[time1]*(49)) +(_b[time2]*(12))+(_b[time3]*(12)) + (_b[time4]*(12)) + (_b[time5]*(8)) + (_b[Sep]*(1)) + (_b[timeafterebola1]*(4))), post

. nlcom _b[September2014]/_b[May2014]

_nl: _b[May2014]/_b[August2014]

** May 2014 to January 2015

estimates use headcount_full_file

estimates replay

nlcom (May2014: _b[_cons] + (_b[population]*(7052.435))+ (_b[time1]*(49)) +(_b[time2]*(12))+(_b[time3]*(12)) + _b[time4]*(12) +(_b[time5]*(4) + (_b[May]*1))) ///

(January2015: _b[_cons] + (_b[population]*(7200.309))+ (_b[time1]*(49)) +(_b[time2]*(12))+(_b[time3]*(12)) + (_b[time4]*(12)) + (_b[time5]*(12)) + (_b[timeafterebola1]*(8)) + (_b[timeafterebola2]*(4))), post

. nlcom _b[January2015]/_b[May2014]

** January 2014 to January 2015

estimates use headcount_full_file

estimates replay

nlcom (January2014: _b[_cons] + (_b[population]*(7052.435))+ (_b[time1]*(49)) +(_b[time2]*(12))+(_b[time3]*(12)) + (_b[time4]*(12))) ///

(January2015: _b[_cons] + (_b[population]*(7200.309))+ (_b[time1]*(49)) +(_b[time2]*(12))+(_b[time3]*(12)) + (_b[time4]*(12)) + (_b[time5]*(12)) + (_b[timeafterebola1]*(8)) + (_b[timeafterebola2]*(4))), post

. nlcom _b[January2015]/_b[January2014]

lincom time5+timeafterebola1

lincom time5+timeafterebola1+timeafterebola2

lincom time6+timeafterebola1+timeafterebola2

lincom time6+timeafterebola1+timeafterebola2+timeafternoebola

lincom time7+timeafterebola1+timeafterebola2+timeafternoebola

tab headcount if headexclude==0 & allmishead==0, missing

tab headoutlier if headexclude==0 & allmishead==0, missing

** REPORTING BASIC YEARLY TRENDS FOR TABLE 2

xtmixed headcount if headexclude==0 & allmishead==0 & month<49 || cluster:, residuals(ar, t(month))

xtmixed headcount if headexclude==0 & allmishead==0 & month>48 & month<61 || cluster:, residuals(ar, t(month))

xtmixed headcount if headexclude==0 & allmishead==0 & month>60 & month<73 || cluster:, residuals(ar, t(month))

xtmixed headcount if headexclude==0 & allmishead==0 & month>72 & month<85 || cluster:, residuals(ar, t(month))

xtmixed headcount if headexclude==0 & allmishead==0 & month>84 & month<90 || cluster:, residuals(ar, t(month))

xtmixed headcount if headexclude==0 & headoutlier!=1 & allmishead==0 & month==91 || cluster:

xtmixed headcount if headexclude==0 & headoutlier!=1 & allmishead==0 & month==92 || cluster:

xtmixed headcount if headexclude==0 & headoutlier!=1 & allmishead==0 & month==93 || cluster:

xtmixed headcount if headexclude==0 & headoutlier!=1 & allmishead==0 & month==96 || cluster:

xtmixed headcount if headexclude==0 & allmishead==0 & timeafterebola1>0 & timeafterebola1<5 || cluster:, residuals(ar, t(month))

xtmixed headcount if headexclude==0 & allmishead==0 & timeafterebola2>0 & timeafterebola2<4 || cluster:, residuals(ar, t(month))

xtmixed headcount if headexclude==0 & allmishead==0 & timeafterebola2>3 & timeafterebola2<8 || cluster:, residuals(ar, t(month))

xtmixed headcount if headexclude==0 & allmishead==0 & timeafternoebola>0 & timeafternoebola<9 || cluster:, residuals(ar, t(month))

xtmixed headcount if headexclude==0 & allmishead==0 & timeafternoebola>8 || cluster:, residuals(ar, t(month))

*********************

**** BCG ************

*********************

estimates use bcg_full_file

estimates replay

** Jan2010 to January 2014

nlcom (jan2014: _b[_cons] + (_b[population]*(6491.185))+ (_b[time1]*(37))) ///

(jan2010: _b[_cons] + (_b[population]*(7052.435))+ (_b[time1]*(49)) +(_b[time2]*(12))+(_b[time3]*(12)) + _b[time4]*(12)), post

. nlcom _b[jan2010]/_b[jan2014]

_nl: _b[jan2010]/_b[jan2014]

**September 2014 compared to May 2014

estimates use bcg_full_file

estimates replay

nlcom (May2014: _b[_cons] + (_b[population]*(7052.435))+ (_b[time1]*(49)) +(_b[time2]*(12))+(_b[time3]*(12)) + _b[time4]*(12) +(_b[time5]*(4) + (_b[May]*1))) ///

(September2014: _b[_cons] + (_b[population]*(7052.435))+ (_b[time1]*(49)) +(_b[time2]*(12))+(_b[time3]*(12)) + (_b[time4]*(12)) + (_b[time5]*(8)) + (_b[Sep]*(1)) + (_b[timeafterebola1]*(4))), post

. nlcom _b[September2014]/_b[May2014]

_nl: _b[May2014]/_b[August2014]

** January 2014 to January 2015

estimates use bcg_full_file

estimates replay

nlcom (January2014: _b[_cons] + (_b[population]*(7052.435))+ (_b[time1]*(49)) +(_b[time2]*(12))+(_b[time3]*(12)) + (_b[time4]*(12))) ///

(January2015: _b[_cons] + (_b[population]*(7200.309))+ (_b[time1]*(49)) +(_b[time2]*(12))+(_b[time3]*(12)) + (_b[time4]*(12)) + (_b[time5]*(12)) + (_b[timeafterebola1]*(8)) + (_b[timeafterebola2]*(4))), post

. nlcom _b[January2015]/_b[January2014]

lincom time5+timeafterebola1

lincom time5+timeafterebola1+timeafterebola2

lincom time6+timeafterebola1+timeafterebola2

lincom time6+timeafterebola1+timeafterebola2+timeafternoebola

lincom time7+timeafterebola1+timeafterebola2+timeafternoebola

tab bcgdosesgiven if bcgexclude==0 & allmisbcg==0, missing

tab bcgdosesoutlier if bcgexclude==0 & allmisbcg==0, missing

** YEARLY TRENDS

xtmixed bcgdosesgiven if bcgexclude==0 & bcgdosesoutlier!=1 & allmisbcg==0 & month<49 || cluster:, residuals(ar, t(month))

xtmixed bcgdosesgiven if bcgexclude==0 & bcgdosesoutlier!=1 & allmisbcg==0 & month>48 & month<61 || cluster:, residuals(ar, t(month))

xtmixed bcgdosesgiven if bcgexclude==0 & bcgdosesoutlier!=1 & allmisbcg==0 & month>60 & month<73 || cluster:, residuals(ar, t(month))

xtmixed bcgdosesgiven if bcgexclude==0 & bcgdosesoutlier!=1 & allmisbcg==0 & month>72 & month<85 || cluster:, residuals(ar, t(month))

xtmixed bcgdosesgiven if bcgexclude==0 & bcgdosesoutlier!=1 & allmisbcg==0 & month>84 & month<90 || cluster:, residuals(ar, t(month))

xtmixed bcgdosesgiven if bcgexclude==0 & bcgdosesoutlier!=1 & allmisbcg==0 & month==91 || cluster:

xtmixed bcgdosesgiven if bcgexclude==0 & bcgdosesoutlier!=1 & allmisbcg==0 & month==92 || cluster:

xtmixed bcgdosesgiven if bcgexclude==0 & bcgdosesoutlier!=1 & allmisbcg==0 & month==93 || cluster:

xtmixed bcgdosesgiven if bcgexclude==0 & bcgdosesoutlier!=1 & allmisbcg==0 & month==96 || cluster:

**EBOLA

xtmixed bcgdosesgiven if bcgexclude==0 & bcgdosesoutlier!=1 & allmisbcg==0 & timeafterebola1>0 & timeafterebola1<5 || cluster:, residuals(ar, t(month))

xtmixed bcgdosesgiven if bcgexclude==0 & bcgdosesoutlier!=1 & allmisbcg==0 & timeafterebola2>0 & timeafterebola2<4 || cluster:, residuals(ar, t(month))

xtmixed bcgdosesgiven if bcgexclude==0 & bcgdosesoutlier!=1 & allmisbcg==0 & timeafterebola2>3 & timeafterebola2<8 || cluster:, residuals(ar, t(month))

xtmixed bcgdosesgiven if bcgexclude==0 & bcgdosesoutlier!=1 & allmisbcg==0 & timeafternoebola>0 & timeafternoebola<9 || cluster:, residuals(ar, t(month))

xtmixed bcgdosesgiven if bcgexclude==0 & bcgdosesoutlier!=1 & allmisbcg==0 & timeafternoebola>8 || cluster:, residuals(ar, t(month))

*********************

**** Measles ********

*********************

** Jan2010 to January 2014

estimates use measles_full_file

estimates replay

nlcom (jan2014: _b[_cons] + (_b[population]*(6491.185))+ (_b[time1]*(37))) ///

(jan2010: _b[_cons] + (_b[population]*(7052.435))+ (_b[time1]*(49)) +(_b[time2]*(12))+(_b[time3]*(12)) + _b[time4]*(12)), post

. nlcom _b[jan2010]/_b[jan2014]

_nl: _b[jan2010]/_b[jan2014]

**September 2014 compared to May 2014

estimates use measles_full_file

estimates replay

nlcom (May2014: _b[_cons] + (_b[population]*(7052.435))+ (_b[time1]*(49)) +(_b[time2]*(12))+(_b[time3]*(12)) + _b[time4]*(12) +(_b[time5]*(4) + (_b[May]*1))) ///

(September2014: _b[_cons] + (_b[population]*(7052.435))+ (_b[time1]*(49)) +(_b[time2]*(12))+(_b[time3]*(12)) + (_b[time4]*(12)) + (_b[time5]*(8)) + (_b[Sep]*(1)) + (_b[timeafterebola1]*(4))), post

. nlcom _b[September2014]/_b[May2014]

** January 2014 to January 2015

estimates use measles_full_file

estimates replay

nlcom (January2014: _b[_cons] + (_b[population]*(7052.435))+ (_b[time1]*(49)) +(_b[time2]*(12))+(_b[time3]*(12)) + (_b[time4]*(12))) ///

(January2015: _b[_cons] + (_b[population]*(7200.309))+ (_b[time1]*(49)) +(_b[time2]*(12))+(_b[time3]*(12)) + (_b[time4]*(12)) + (_b[time5]*(12)) + (_b[timeafterebola1]*(8)) + (_b[timeafterebola2]*(4))), post

. nlcom _b[January2015]/_b[January2014]

**May 2015 compared to May 2014

estimates use measles_full_file

estimates replay

nlcom (May2014: _b[_cons] + (_b[population]*(7052.435))+ (_b[time1]*(49)) +(_b[time2]*(12))+(_b[time3]*(12)) + _b[time4]*(12) +(_b[time5]*(4) + (_b[May]*1))) ///

(May2015: _b[_cons] + (_b[population]*(7200.309))+ (_b[time1]*(49)) +(_b[time2]*(12))+(_b[time3]*(12)) + (_b[time4]*(12)) + (_b[time5]*(12)) + (_b[time6]*(4)) + (_b[May]*(1)) + (_b[timeafterebola1]*(12)) + (_b[timeafterebola2]*(8)) + (_b[timeafternoebola]*(1))), post

. nlcom _b[May2015]/_b[May2014]

lincom time5+timeafterebola1

lincom time5+timeafterebola1+timeafterebola2

lincom time6+timeafterebola1+timeafterebola2

lincom time6+timeafterebola1+timeafterebola2+timeafternoebola

lincom time7+timeafterebola1+timeafterebola2+timeafternoebola

tab measlesdosesgiven if measlesexclude==0 & allmismeasles==0, missing

tab measlesdosesoutlier if bcgexclude==0 & allmisbcg==0, missing

** YEARLY TRENDS

xtmixed measlesdosesgiven if measlesexclude==0 & allmismeasles==0 & month<49 || cluster:, residuals(ar, t(month))

xtmixed measlesdosesgiven if measlesexclude==0 & allmismeasles==0 & month>48 & month<61 || cluster:, residuals(ar, t(month))

xtmixed measlesdosesgiven if measlesexclude==0 & allmismeasles==0 & month>60 & month<73 || cluster:, residuals(ar, t(month))

xtmixed measlesdosesgiven if measlesexclude==0 & allmismeasles==0 & month>72 & month<85 || cluster:, residuals(ar, t(month))

xtmixed measlesdosesgiven if measlesexclude==0 & allmismeasles==0 & month>84 & month<90 || cluster:, residuals(ar, t(month))

xtmixed measlesdosesgiven if measlesexclude==0 & allmismeasles==0 & month==91 || cluster:

xtmixed measlesdosesgiven if measlesexclude==0 & allmismeasles==0 & month==92 || cluster:

xtmixed measlesdosesgiven if measlesexclude==0 & allmismeasles==0 & month==93 || cluster:

xtmixed measlesdosesgiven if measlesexclude==0 & allmismeasles==0 & month==96 || cluster:

xtmixed measlesdosesgiven if measlesexclude==0 & allmismeasles==0 & month==100 || cluster:

**EBOLA

xtmixed measlesdosesgiven if measlesexclude==0 & allmismeasles==0 & timeafterebola1>0 & timeafterebola1<5 || cluster:, residuals(ar, t(month))

xtmixed measlesdosesgiven if measlesexclude==0 & allmismeasles==0 & timeafterebola2>0 & timeafterebola2<4 || cluster:, residuals(ar, t(month))

xtmixed measlesdosesgiven if measlesexclude==0 & allmismeasles==0 & timeafterebola2>3 & timeafterebola2<8 || cluster:, residuals(ar, t(month))

xtmixed measlesdosesgiven if measlesexclude==0 & allmismeasles==0 & timeafternoebola>0 & timeafternoebola<9 || cluster:, residuals(ar, t(month))

xtmixed measlesdosesgiven if measlesexclude==0 & allmismeasles==0 & timeafternoebola>8 || cluster:, residuals(ar, t(month))

*********************

**** Penta ********

*********************

** Jan2010 to January 2014

estimates use penta_full_file

estimates replay

nlcom (jan2014: _b[_cons] + (_b[population]*(6491.185))+ (_b[time1]*(37))) ///

(jan2010: _b[_cons] + (_b[population]*(7052.435))+ (_b[time1]*(49)) +(_b[time2]*(12))+(_b[time3]*(12)) + _b[time4]*(12)), post

. nlcom _b[jan2010]/_b[jan2014]

_nl: _b[jan2010]/_b[jan2014]

**September 2014 compared to May 2014

estimates use penta_full_file

estimates replay

nlcom (May2014: _b[_cons] + (_b[population]*(7052.435))+ (_b[time1]*(49)) +(_b[time2]*(12))+(_b[time3]*(12)) + _b[time4]*(12) +(_b[time5]*(4) + (_b[May]*1))) ///

(September2014: _b[_cons] + (_b[population]*(7052.435))+ (_b[time1]*(49)) +(_b[time2]*(12))+(_b[time3]*(12)) + (_b[time4]*(12)) + (_b[time5]*(8)) + (_b[Sep]*(1)) + (_b[timeafterebola1]*(4))), post

. nlcom _b[September2014]/_b[May2014]

** January 2014 to January 2015

estimates use penta_full_file

estimates replay

nlcom (January2014: _b[_cons] + (_b[population]*(7052.435))+ (_b[time1]*(49)) +(_b[time2]*(12))+(_b[time3]*(12)) + (_b[time4]*(12))) ///

(January2015: _b[_cons] + (_b[population]*(7200.309))+ (_b[time1]*(49)) +(_b[time2]*(12))+(_b[time3]*(12)) + (_b[time4]*(12)) + (_b[time5]*(12)) + (_b[timeafterebola1]*(8)) + (_b[timeafterebola2]*(4))), post

. nlcom _b[January2015]/_b[January2014]

lincom time5+timeafterebola1

lincom time5+timeafterebola1+timeafterebola2

lincom time6+timeafterebola1+timeafterebola2

lincom time6+timeafterebola1+timeafterebola2+timeafternoebola

lincom time7+timeafterebola1+timeafterebola2+timeafternoebola

** YEARLY TRENDS

xtmixed pentavalent1given if pentaexclude==0 & pentadosesoutlier!=1 & allmispenta==0 & month<49 || cluster:, residuals(ar, t(month))

xtmixed pentavalent1given if pentaexclude==0 & pentadosesoutlier!=1 & allmispenta==0 & month>48 & month<61 || cluster:, residuals(ar, t(month))

xtmixed pentavalent1given if pentaexclude==0 & pentadosesoutlier!=1 & allmispenta==0 & month>60 & month<73 || cluster:, residuals(ar, t(month))

xtmixed pentavalent1given if pentaexclude==0 & pentadosesoutlier!=1 & allmispenta==0 & month>72 & month<85 || cluster:, residuals(ar, t(month))

xtmixed pentavalent1given if pentaexclude==0 & pentadosesoutlier!=1 & allmispenta==0 & month>84 & month<90 || cluster:, residuals(ar, t(month))

xtmixed pentavalent1given if pentaexclude==0 & pentadosesoutlier!=1 & allmispenta==0 & month==91 || cluster:

xtmixed pentavalent1given if pentaexclude==0 & pentadosesoutlier!=1 & allmispenta==0 & month==92 || cluster:

xtmixed pentavalent1given if pentaexclude==0 & pentadosesoutlier!=1 & allmispenta==0 & month==93 || cluster:

xtmixed pentavalent1given if pentaexclude==0 & pentadosesoutlier!=1 & allmispenta==0 & month==96 || cluster:

**EBOLA

xtmixed pentavalent1given if pentaexclude==0 & pentadosesoutlier!=1 & allmispenta==0 & timeafterebola1>0 & timeafterebola1<5 || cluster:, residuals(ar, t(month))

xtmixed pentavalent1given if pentaexclude==0 & pentadosesoutlier!=1 & allmispenta==0 & timeafterebola2>0 & timeafterebola2<4 || cluster:, residuals(ar, t(month))

xtmixed pentavalent1given if pentaexclude==0 & pentadosesoutlier!=1 & allmispenta==0 & timeafterebola2>3 & timeafterebola2<8 || cluster:, residuals(ar, t(month))

xtmixed pentavalent1given if pentaexclude==0 & pentadosesoutlier!=1 & allmispenta==0 & timeafternoebola>0 & timeafternoebola<9 || cluster:, residuals(ar, t(month))

xtmixed pentavalent1given if pentaexclude==0 & pentadosesoutlier!=1 & allmispenta==0 & timeafternoebola>8 || cluster:, residuals(ar, t(month))

*********************

**** 1st ANC ********

*********************

** Jan2010 to January 2014

estimates use ANC_full_file

estimates replay

nlcom (jan2014: _b[_cons] + (_b[population]*(6491.185))+ (_b[time1]*(37))) ///

(jan2010: _b[_cons] + (_b[population]*(7052.435))+ (_b[time1]*(49)) +(_b[time2]*(12))+(_b[time3]*(12)) + _b[time4]*(12)), post

. nlcom _b[jan2010]/_b[jan2014]

_nl: _b[jan2010]/_b[jan2014]

**September 2014 compared to May 2014

estimates use ANC_full_file

estimates replay

nlcom (May2014: _b[_cons] + (_b[population]*(7052.435))+ (_b[time1]*(49)) +(_b[time2]*(12))+(_b[time3]*(12)) + _b[time4]*(12) +(_b[time5]*(4) + (_b[May]*1))) ///

(September2014: _b[_cons] + (_b[population]*(7052.435))+ (_b[time1]*(49)) +(_b[time2]*(12))+(_b[time3]*(12)) + (_b[time4]*(12)) + (_b[time5]*(8)) + (_b[Sep]*(1)) + (_b[timeafterebola1]*(4))), post

. nlcom _b[September2014]/_b[May2014]

** January 2014 to January 2015

estimates use ANC_full_file

estimates replay

nlcom (January2014: _b[_cons] + (_b[population]*(7052.435))+ (_b[time1]*(49)) +(_b[time2]*(12))+(_b[time3]*(12)) + (_b[time4]*(12))) ///

(January2015: _b[_cons] + (_b[population]*(7200.309))+ (_b[time1]*(49)) +(_b[time2]*(12))+(_b[time3]*(12)) + (_b[time4]*(12)) + (_b[time5]*(12)) + (_b[timeafterebola1]*(8)) + (_b[timeafterebola2]*(4))), post

. nlcom _b[January2015]/_b[January2014]

lincom time5+timeafterebola1

lincom time5+timeafterebola1+timeafterebola2

lincom time6+timeafterebola1+timeafterebola2

lincom time6+timeafterebola1+timeafterebola2+timeafternoebola

lincom time7+timeafterebola1+timeafterebola2+timeafternoebola

** YEARLY TRENDS

xtmixed stancvisit if antenatalexclude==0 & allmisantenatal==0 & month<49 || cluster:, residuals(ar, t(month))

xtmixed stancvisit if antenatalexclude==0 & allmisantenatal==0 & month>48 & month<61 || cluster:, residuals(ar, t(month))

xtmixed stancvisit if antenatalexclude==0 & allmisantenatal==0 & month>60 & month<73 || cluster:, residuals(ar, t(month))

xtmixed stancvisit if antenatalexclude==0 & allmisantenatal==0 & month>72 & month<85 || cluster:, residuals(ar, t(month))

xtmixed stancvisit if antenatalexclude==0 & allmisantenatal==0 & month>84 & month<90 || cluster:, residuals(ar, t(month))

xtmixed stancvisit if antenatalexclude==0 & allmisantenatal==0 & month==91 || cluster:

xtmixed stancvisit if antenatalexclude==0 & allmisantenatal==0 & month==92 || cluster:

xtmixed stancvisit if antenatalexclude==0 & allmisantenatal==0 & month==93 || cluster:

xtmixed stancvisit if antenatalexclude==0 & allmisantenatal==0 & month==96 || cluster:

**EBOLA

xtmixed stancvisit if antenatalexclude==0 & allmisantenatal==0 & timeafterebola1>0 & timeafterebola1<5 || cluster:, residuals(ar, t(month))

xtmixed stancvisit if antenatalexclude==0 & allmisantenatal==0 & timeafterebola2>0 & timeafterebola2<4 || cluster:, residuals(ar, t(month))

xtmixed stancvisit if antenatalexclude==0 & allmisantenatal==0 & timeafterebola2>3 & timeafterebola2<8 || cluster:, residuals(ar, t(month))

xtmixed stancvisit if antenatalexclude==0 & allmisantenatal==0 & timeafternoebola>0 & timeafternoebola<9 || cluster:, residuals(ar, t(month))

xtmixed stancvisit if antenatalexclude==0 & allmisantenatal==0 & timeafternoebola>8 || cluster:, residuals(ar, t(month))

*********************

**** BIRTHS********

*********************

** Jan2010 to January 2014

estimates use delivery_full_file

estimates replay

nlcom (jan2014: _b[_cons] + (_b[population]*(6491.185))+ (_b[time1]*(37))) ///

(jan2010: _b[_cons] + (_b[population]*(7052.435))+ (_b[time1]*(49)) +(_b[time2]*(12))+(_b[time3]*(12)) + _b[time4]*(12)), post

. nlcom _b[jan2010]/_b[jan2014]

_nl: _b[jan2010]/_b[jan2014]

**September 2014 compared to May 2014

estimates use delivery_full_file

estimates replay

nlcom (May2014: _b[_cons] + (_b[population]*(7052.435))+ (_b[time1]*(49)) +(_b[time2]*(12))+(_b[time3]*(12)) + _b[time4]*(12) +(_b[time5]*(4) + (_b[May]*1))) ///

(September2014: _b[_cons] + (_b[population]*(7052.435))+ (_b[time1]*(49)) +(_b[time2]*(12))+(_b[time3]*(12)) + (_b[time4]*(12)) + (_b[time5]*(8)) + (_b[Sep]*(1)) + (_b[timeafterebola1]*(4))), post

. nlcom _b[September2014]/_b[May2014]

** January 2014 to January 2015

estimates use delivery_full_file

estimates replay

nlcom (January2014: _b[_cons] + (_b[population]*(7052.435))+ (_b[time1]*(49)) +(_b[time2]*(12))+(_b[time3]*(12)) + (_b[time4]*(12))) ///

(January2015: _b[_cons] + (_b[population]*(7200.309))+ (_b[time1]*(49)) +(_b[time2]*(12))+(_b[time3]*(12)) + (_b[time4]*(12)) + (_b[time5]*(12)) + (_b[timeafterebola1]*(8)) + (_b[timeafterebola2]*(4))), post

. nlcom _b[January2015]/_b[January2014]

lincom time5+timeafterebola1

lincom time5+timeafterebola1+timeafterebola2

lincom time6+timeafterebola1+timeafterebola2

lincom time6+timeafterebola1+timeafterebola2+timeafternoebola

lincom time7+timeafterebola1+timeafterebola2+timeafternoebola

** YEARLY TRENDS

xtmixed skilleddelfacility if deliveryexclude==0 & deliverydosesoutlier!=1 & allmisdelivery==0 & month<49 || cluster:, residuals(ar, t(month))

xtmixed skilleddelfacility if deliveryexclude==0 & deliverydosesoutlier!=1 & allmisdelivery==0 & month>48 & month<61 || cluster:, residuals(ar, t(month))

xtmixed skilleddelfacility if deliveryexclude==0 & deliverydosesoutlier!=1 & allmisdelivery==0 & month>60 & month<73 || cluster:, residuals(ar, t(month))

xtmixed skilleddelfacility if deliveryexclude==0 & deliverydosesoutlier!=1 & allmisdelivery==0 & month>72 & month<85 || cluster:, residuals(ar, t(month))

xtmixed skilleddelfacility if deliveryexclude==0 & deliverydosesoutlier!=1 & allmisdelivery==0 & month>84 & month<90 || cluster:, residuals(ar, t(month))

xtmixed skilleddelfacility if deliveryexclude==0 & deliverydosesoutlier!=1 & allmisdelivery==0 & month==91 || cluster:

xtmixed skilleddelfacility if deliveryexclude==0 & deliverydosesoutlier!=1 & allmisdelivery==0 & month==92 || cluster:

xtmixed skilleddelfacility if deliveryexclude==0 & deliverydosesoutlier!=1 & allmisdelivery==0 & month==93 || cluster:

xtmixed skilleddelfacility if deliveryexclude==0 & deliverydosesoutlier!=1 & allmisdelivery==0 & month==96 || cluster:

**EBOLA

xtmixed skilleddelfacility if deliveryexclude==0 & deliverydosesoutlier!=1 & allmisdelivery==0 & timeafterebola1>0 & timeafterebola1<5 || cluster:, residuals(ar, t(month))

xtmixed skilleddelfacility if deliveryexclude==0 & deliverydosesoutlier!=1 & allmisdelivery==0 & timeafterebola2>0 & timeafterebola2<4 || cluster:, residuals(ar, t(month))

xtmixed skilleddelfacility if deliveryexclude==0 & deliverydosesoutlier!=1 & allmisdelivery==0 & timeafterebola2>3 & timeafterebola2<8 || cluster:, residuals(ar, t(month))

xtmixed skilleddelfacility if deliveryexclude==0 & deliverydosesoutlier!=1 & allmisdelivery==0 & timeafternoebola>0 & timeafternoebola<9 || cluster:, residuals(ar, t(month))

xtmixed skilleddelfacility if deliveryexclude==0 & deliverydosesoutlier!=1 & allmisdelivery==0 & timeafternoebola>8 || cluster:, residuals(ar, t(month))

*********************

**** ACT ********

*********************

estimates use ACT_full_file

estimates replay

** Jan2010 to January 2014

nlcom (jan2014: _b[_cons] + (_b[population]*(6491.185))+ (_b[time1]*(37))) ///

(jan2010: _b[_cons] + (_b[population]*(7052.435))+ (_b[time1]*(49)) +(_b[time2]*(12))+(_b[time3]*(12)) + _b[time4]*(12)), post

. nlcom _b[jan2010]/_b[jan2014]

_nl: _b[jan2010]/_b[jan2014]

**September 2014 compared to May 2014

estimates use ACT_full_file

estimates replay

nlcom (May2014: _b[_cons] + (_b[population]*(7052.435))+ (_b[time1]*(49)) +(_b[time2]*(12))+(_b[time3]*(12)) + _b[time4]*(12) +(_b[time5]*(4) + (_b[May]*1))) ///

(September2014: _b[_cons] + (_b[population]*(7052.435))+ (_b[time1]*(49)) +(_b[time2]*(12))+(_b[time3]*(12)) + (_b[time4]*(12)) + (_b[time5]*(8)) + (_b[Sep]*(1)) + (_b[timeafterebola1]*(4))), post

. nlcom _b[September2014]/_b[May2014]

** January 2014 to January 2015

estimates use ACT_full_file

estimates replay

nlcom (January2014: _b[_cons] + (_b[population]*(7052.435))+ (_b[time1]*(49)) +(_b[time2]*(12))+(_b[time3]*(12)) + (_b[time4]*(12))) ///

(January2015: _b[_cons] + (_b[population]*(7200.309))+ (_b[time1]*(49)) +(_b[time2]*(12))+(_b[time3]*(12)) + (_b[time4]*(12)) + (_b[time5]*(12)) + (_b[timeafterebola1]*(8)) + (_b[timeafterebola2]*(4))), post

. nlcom _b[January2015]/_b[January2014]

**Dec 2016 compared to Dec 2013

estimates use ACT_full_file

estimates replay

nlcom (Dec2013: (_b[_cons]) + (_b[population]*(6902.414))+ (_b[time1]*(49)) +(_b[time2]*(12))+(_b[time3]*(12)) + (_b[time4]*(11)) + (_b[Dec]*(1))) ///

(Dec2016: (_b[_cons]) + (_b[population]*(7377.204))+ (_b[time1]*(49)) +(_b[time2]*(12))+(_b[time3]*(12)) + (_b[time4]*(12)) + (_b[time5]*(12)) + (_b[time6]*(12)) + (_b[time7]*(11)) + (_b[Dec]*(1)) + (_b[timeafternoebola]*(20)) + (_b[timeafterebola1]*(31)) + (_b[timeafterebola2]*(27))), post

. nlcom _b[Dec2016]/_b[Dec2013]

nlcom log(_b[Dec2016]) - log(_b[Dec2013])

lincom time5+timeafterebola1

lincom time5+timeafterebola1+timeafterebola2

lincom time6+timeafterebola1+timeafterebola2

lincom time6+timeafterebola1+timeafterebola2+timeafternoebola

lincom time7+timeafterebola1+timeafterebola2+timeafternoebola

** YEARLY TRENDS

xtmixed acttreatment if actexclude==0 & actdosesoutlier!=1 & allmisact==0 & month<49 || cluster:, residuals(ar, t(month))

xtmixed acttreatment if actexclude==0 & actdosesoutlier!=1 & allmisact==0 & month>48 & month<61 || cluster:, residuals(ar, t(month))

xtmixed acttreatment if actexclude==0 & actdosesoutlier!=1 & allmisact==0 & month>60 & month<73 || cluster:, residuals(ar, t(month))

xtmixed acttreatment if actexclude==0 & actdosesoutlier!=1 & allmisact==0 & month>72 & month<85 || cluster:, residuals(ar, t(month))

xtmixed acttreatment if actexclude==0 & actdosesoutlier!=1 & allmisact==0 & month>84 & month<90 || cluster:, residuals(ar, t(month))

xtmixed acttreatment if actexclude==0 & actdosesoutlier!=1 & allmisact==0 & month==91 || cluster:

xtmixed acttreatment if actexclude==0 & actdosesoutlier!=1 & allmisact==0 & month==92 || cluster:

xtmixed acttreatment if actexclude==0 & actdosesoutlier!=1 & allmisact==0 & month==93 || cluster:

xtmixed acttreatment if actexclude==0 & actdosesoutlier!=1 & allmisact==0 & month==96 || cluster:

**EBOLA

xtmixed acttreatment if actexclude==0 & actdosesoutlier!=1 & allmisact==0 & timeafterebola1>0 & timeafterebola1<5 || cluster:, residuals(ar, t(month))

xtmixed acttreatment if actexclude==0 & actdosesoutlier!=1 & allmisact==0 & timeafterebola2>0 & timeafterebola2<4 || cluster:, residuals(ar, t(month))

xtmixed acttreatment if actexclude==0 & actdosesoutlier!=1 & allmisact==0 & timeafterebola2>3 & timeafterebola2<8 || cluster:, residuals(ar, t(month))

xtmixed acttreatment if actexclude==0 & actdosesoutlier!=1 & allmisact==0 & timeafternoebola>0 & timeafternoebola<9 || cluster:, residuals(ar, t(month))

xtmixed acttreatment if actexclude==0 & actdosesoutlier!=1 & allmisact==0 & timeafternoebola>8 || cluster:, residuals(ar, t(month))

*********************

**** PNC ********

*********************

** Jan2010 to January 2014

estimates use PNC_full_file

estimates replay

**September 2014 compared to May 2014

estimates use PNC_full_file

estimates replay

nlcom (May2014: _b[_cons] + (_b[population]*(7052.435))+ (_b[time3]*(12)) + _b[time4]*(12) +(_b[time5]*(4) + (_b[May]*1))) ///

(September2014: _b[_cons] + (_b[population]*(7052.435)) +(_b[time3]*(12)) + (_b[time4]*(12)) + (_b[time5]*(8)) + (_b[Sep]*(1)) + (_b[timeafterebola1]*(4))), post

. nlcom _b[September2014]/_b[May2014]

** January 2014 to January 2015

estimates use PNC_full_file

estimates replay

nlcom (January2014: _b[_cons] + (_b[population]*(7052.435))+ (_b[time3]*(12)) + (_b[time4]*(12))) ///

(January2015: _b[_cons] + (_b[population]*(7200.309))+ (_b[time3]*(12)) + (_b[time4]*(12)) + (_b[time5]*(12)) + (_b[timeafterebola1]*(8)) + (_b[timeafterebola2]*(4))), post

. nlcom _b[January2015]/_b[January2014]

lincom time5+timeafterebola1

lincom time5+timeafterebola1+timeafterebola2

lincom time6+timeafterebola1+timeafterebola2

lincom time6+timeafterebola1+timeafterebola2+timeafternoebola

lincom time7+timeafterebola1+timeafterebola2+timeafternoebola

** YEARLY TRENDS

xtmixed pncwithin6weeks if pncexclude==0 & pncdosesoutlier!=1 & allmispnc==0 & month>60 & month<73 || cluster:, residuals(ar, t(month))

xtmixed pncwithin6weeks if pncexclude==0 & pncdosesoutlier!=1 & allmispnc==0 & month>72 & month<85 || cluster:, residuals(ar, t(month))

xtmixed pncwithin6weeks if pncexclude==0 & pncdosesoutlier!=1 & allmispnc==0 & month>84 & month<90 || cluster:, residuals(ar, t(month))

xtmixed pncwithin6weeks if pncexclude==0 & pncdosesoutlier!=1 & allmispnc==0 & month==91 || cluster:

xtmixed pncwithin6weeks if pncexclude==0 & pncdosesoutlier!=1 & allmispnc==0 & month==92 || cluster:

xtmixed pncwithin6weeks if pncexclude==0 & pncdosesoutlier!=1 & allmispnc==0 & month==93 || cluster:

xtmixed pncwithin6weeks if pncexclude==0 & pncdosesoutlier!=1 & allmispnc==0 & month==96 || cluster:

**EBOLA

xtmixed pncwithin6weeks if pncexclude==0 & pncdosesoutlier!=1 & allmispnc==0 & timeafterebola1>0 & timeafterebola1<5 || cluster:, residuals(ar, t(month))

xtmixed pncwithin6weeks if pncexclude==0 & pncdosesoutlier!=1 & allmispnc==0 & timeafterebola2>0 & timeafterebola2<4 || cluster:, residuals(ar, t(month))

xtmixed pncwithin6weeks if pncexclude==0 & pncdosesoutlier!=1 & allmispnc==0 & timeafterebola2>3 & timeafterebola2<8 || cluster:, residuals(ar, t(month))

xtmixed pncwithin6weeks if pncexclude==0 & pncdosesoutlier!=1 & allmispnc==0 & timeafternoebola>0 & timeafternoebola<9 || cluster:, residuals(ar, t(month))

xtmixed pncwithin6weeks if pncexclude==0 & pncdosesoutlier!=1 & allmispnc==0 & timeafternoebola>8 || cluster:, residuals(ar, t(month))

*********************

**** ARIs ********

*********************

** Jan2010 to January 2014

estimates use ARI_full_file

estimates replay

**September 2014 compared to May 2014

estimates use ARI_full_file

estimates replay

nlcom (May2014: _b[_cons] + (_b[population]*(7052.435))+ (_b[time3]*(12)) + _b[time4]*(12) +(_b[time5]*(4) + (_b[May]*1))) ///

(September2014: _b[_cons] + (_b[population]*(7052.435)) +(_b[time3]*(12)) + (_b[time4]*(12)) + (_b[time5]*(8)) + (_b[Sep]*(1)) + (_b[timeafterebola1]*(4))), post

. nlcom _b[September2014]/_b[May2014]

** January 2014 to January 2015

estimates use ARI_full_file

estimates replay

nlcom (January2014: _b[_cons] + (_b[population]*(7052.435))+ (_b[time3]*(12)) + (_b[time4]*(12))) ///

(January2015: _b[_cons] + (_b[population]*(7200.309))+ (_b[time3]*(12)) + (_b[time4]*(12)) + (_b[time5]*(12)) + (_b[timeafterebola1]*(8)) + (_b[timeafterebola2]*(4))), post

. nlcom _b[January2015]/_b[January2014]

lincom time5+timeafterebola1

lincom time5+timeafterebola1+timeafterebola2

lincom time6+timeafterebola1+timeafterebola2

lincom time6+timeafterebola1+timeafterebola2+timeafternoebola

lincom time7+timeafterebola1+timeafterebola2+timeafternoebola

** YEARLY TRENDS

xtmixed ari if respiratoryexclude==0 & respiratorydosesoutlier!=1 & allmisrespiratory==0 & month<49 || cluster:, residuals(ar, t(month))

xtmixed ari if respiratoryexclude==0 & respiratorydosesoutlier!=1 & allmisrespiratory==0 & month>48 & month<61 || cluster:, residuals(ar, t(month))

xtmixed ari if respiratoryexclude==0 & respiratorydosesoutlier!=1 & allmisrespiratory==0 & month>60 & month<73 || cluster:, residuals(ar, t(month))

xtmixed ari if respiratoryexclude==0 & respiratorydosesoutlier!=1 & allmisrespiratory==0 & month>72 & month<85 || cluster:, residuals(ar, t(month))

xtmixed ari if respiratoryexclude==0 & respiratorydosesoutlier!=1 & allmisrespiratory==0 & month>84 & month<90 || cluster:, residuals(ar, t(month))

xtmixed ari if respiratoryexclude==0 & respiratorydosesoutlier!=1 & allmisrespiratory==0 & month==91 || cluster:

xtmixed ari if respiratoryexclude==0 & respiratorydosesoutlier!=1 & allmisrespiratory==0 & month==92 || cluster:

xtmixed ari if respiratoryexclude==0 & respiratorydosesoutlier!=1 & allmisrespiratory==0 & month==93 || cluster:

xtmixed ari if respiratoryexclude==0 & respiratorydosesoutlier!=1 & allmisrespiratory==0 & month==96 || cluster:

**EBOLA

xtmixed ari if respiratoryexclude==0 & respiratorydosesoutlier!=1 & allmisrespiratory==0 & timeafterebola1>0 & timeafterebola1<5 || cluster:, residuals(ar, t(month))

xtmixed ari if respiratoryexclude==0 & respiratorydosesoutlier!=1 & allmisrespiratory==0 & timeafterebola2>0 & timeafterebola2<4 || cluster:, residuals(ar, t(month))

xtmixed ari if respiratoryexclude==0 & respiratorydosesoutlier!=1 & allmisrespiratory==0 & timeafterebola2>3 & timeafterebola2<8 || cluster:, residuals(ar, t(month))

xtmixed ari if respiratoryexclude==0 & respiratorydosesoutlier!=1 & allmisrespiratory==0 & timeafternoebola>0 & timeafternoebola<9 || cluster:, residuals(ar, t(month))

xtmixed ari if respiratoryexclude==0 & respiratorydosesoutlier!=1 & allmisrespiratory==0 & timeafternoebola>8 || cluster:, residuals(ar, t(month))

*********************

**** DEPO ********

*********************

** Jan2010 to January 2014

estimates use depo_full_file

estimates replay

nlcom (jan2014: _b[_cons] + (_b[population]*(6491.185))+ (_b[time1]*(37))) ///

(jan2010: _b[_cons] + (_b[population]*(7052.435))+ (_b[time1]*(49)) +(_b[time2]*(12))+(_b[time3]*(12)) + _b[time4]*(12)), post

. nlcom _b[jan2010]/_b[jan2014]

_nl: _b[jan2010]/_b[jan2014]

**September 2014 compared to May 2014

estimates use depo_full_file

estimates replay

nlcom (May2014: _b[_cons] + (_b[population]*(7052.435))+ (_b[time1]*(49)) +(_b[time2]*(12))+(_b[time3]*(12)) + _b[time4]*(12) +(_b[time5]*(4) + (_b[May]*1))) ///

(September2014: _b[_cons] + (_b[population]*(7052.435))+ (_b[time1]*(49)) +(_b[time2]*(12))+(_b[time3]*(12)) + (_b[time4]*(12)) + (_b[time5]*(8)) + (_b[Sep]*(1)) + (_b[timeafterebola1]*(4))), post

. nlcom _b[September2014]/_b[May2014]

** January 2014 to January 2015

estimates use depo_full_file

estimates replay

nlcom (January2014: _b[_cons] + (_b[population]*(7052.435))+ (_b[time1]*(49)) +(_b[time2]*(12))+(_b[time3]*(12)) + (_b[time4]*(12))) ///

(January2015: _b[_cons] + (_b[population]*(7200.309))+ (_b[time1]*(49)) +(_b[time2]*(12))+(_b[time3]*(12)) + (_b[time4]*(12)) + (_b[time5]*(12)) + (_b[timeafterebola1]*(8)) + (_b[timeafterebola2]*(4))), post

. nlcom _b[January2015]/_b[January2014]

lincom time5+timeafterebola1

lincom time5+timeafterebola1+timeafterebola2

lincom time6+timeafterebola1+timeafterebola2

lincom time6+timeafterebola1+timeafterebola2+timeafternoebola

lincom time7+timeafterebola1+timeafterebola2+timeafternoebola

** YEARLY TRENDS

xtmixed depoprovera if depoexclude==0 & depodosesoutlier!=1 & allmisdepo==0 & month<49 || cluster:, residuals(ar, t(month))

xtmixed depoprovera if depoexclude==0 & depodosesoutlier!=1 & allmisdepo==0 & month>48 & month<61 || cluster:, residuals(ar, t(month))

xtmixed depoprovera if depoexclude==0 & depodosesoutlier!=1 & allmisdepo==0 & month>60 & month<73 || cluster:, residuals(ar, t(month))

xtmixed depoprovera if depoexclude==0 & depodosesoutlier!=1 & allmisdepo==0 & month>72 & month<85 || cluster:, residuals(ar, t(month))

xtmixed depoprovera if depoexclude==0 & depodosesoutlier!=1 & allmisdepo==0 & month>84 & month<90 || cluster:, residuals(ar, t(month))

xtmixed depoprovera if depoexclude==0 & depodosesoutlier!=1 & allmisdepo==0 & month==91 || cluster:

xtmixed depoprovera if depoexclude==0 & depodosesoutlier!=1 & allmisdepo==0 & month==92 || cluster:

xtmixed depoprovera if depoexclude==0 & depodosesoutlier!=1 & allmisdepo==0 & month==93 || cluster:

xtmixed depoprovera if depoexclude==0 & depodosesoutlier!=1 & allmisdepo==0 & month==96 || cluster:

**EBOLA

xtmixed depoprovera if depoexclude==0 & depodosesoutlier!=1 & allmisdepo==0 & timeafterebola1>0 & timeafterebola1<5 || cluster:, residuals(ar, t(month))

xtmixed depoprovera if depoexclude==0 & depodosesoutlier!=1 & allmisdepo==0 & timeafterebola2>0 & timeafterebola2<4 || cluster:, residuals(ar, t(month))

xtmixed depoprovera if depoexclude==0 & depodosesoutlier!=1 & allmisdepo==0 & timeafterebola2>3 & timeafterebola2<8 || cluster:, residuals(ar, t(month))

xtmixed depoprovera if depoexclude==0 & depodosesoutlier!=1 & allmisdepo==0 & timeafternoebola>0 & timeafternoebola<9 || cluster:, residuals(ar, t(month))

xtmixed depoprovera if depoexclude==0 & depodosesoutlier!=1 & allmisdepo==0 & timeafternoebola>8 || cluster:, residuals(ar, t(month))

**************************************************************

* COMMANDS FOR CREATING FORECASTS AND COMPARING TO OBSERVED

**************************************************************

*********************

**** HEAD COUNT *****

*********************

set scheme plotplain

clear

use analysisdataset

**HEAD COUNT XTMIXED_NO EBOLA

xtmixed headcount time1-time4 population Feb Mar Apr May Jun July Aug Sep Oct ///

Nov Dec ///

|| cluster:time1-time4 ///

if headexclude==0 & headoutlier!=1 & allmishead==0 & month<=89, residuals(ar, t(month))

preserve

// The betas from the fixed part comes from multivariate normal dist of B means and V covariances

// For reduced model

clear

est use headcount_reduc

mat b = e(b)

matselrc b B, col(1/17) // Extract the fixed part betas

mat v = e(V)

matselrc v V, col(1/17) row(1/17) // Extract the fixed part covariances

local nam = ""

forvalues i = 1/17 {

local nam = "`nam' red`i'"

}

// di "`nam'"

drawnorm `nam', cov(V) mean(B) n(1000) seed(846031265)

gen id = _n

save simbetas_reduced, replace

// For Full model

clear

est use headcount_full_file

mat b = e(b)

matselrc b B, col(1/23) // Extract the fixed part betas

mat v = e(V)

matselrc v V, col(1/23) row(1/23) // Extract the fixed part covariances

local nam = ""

forvalues i = 1/23 {

local nam = "`nam' ful`i'"

}

drawnorm `nam', cov(V) mean(B) n(1000) seed(846031265)

gen id = _n

save simbetas_full, replace

merge 1:1 id using simbetas_reduced

drop _merge

save simbetas, replace

restore

// Now lets predict from each month

gen one = 1

collapse (sum) one (p75) population , by(month time1-time7 /// population

Feb Mar Apr May Jun July Aug Sep Oct ///

Nov Dec timeafterebola1 timeafterebola2 timeafternoebola)

drop one

// We need 1000 predictions per month

expand 1000

bysort month: gen id = _n

order id month

sort id month

// Merge to our simulated betas

merge m:1 id using simbetas

gen predfull = time1 * ful1 + ///

time2 * ful2 + ///

time3 * ful3 + ///

time4 * ful4 + ///

time5 * ful5 + ///

time6 * ful6 + ///

time7 * ful7 + ///

population * ful8 + /// population coeficient

Feb * ful9 + ///

Mar * ful10 + ///

Apr * ful11 + ///

May * ful12 + ///

Jun * ful13 + ///

July * ful14 + ///

Aug * ful15 + ///

Sep * ful16 + ///

Oct * ful17 + ///

Nov * ful18 + ///

Dec * ful19 + ///

timeafterebola1 * ful20 + ///

timeafterebola2 * ful21 + ///

timeafternoebola * ful22 + ///

ful23 // The intercept

gen predred = time1 * red1 + ///

time2 * red2 + ///

time3 * red3 + ///

time4 * red4 + ///

population * red5 + /// population coeficient

Feb * red6 + ///

Mar * red7 + ///

Apr * red8 + ///

May * red9 + ///

Jun * red10 + ///

July * red11 + ///

Aug * red12 + ///

Sep * red13 + ///

Oct * red14 + ///

Nov * red15 + ///

Dec * red16 + ///

red17 // The intercept

************ OUR PRECIOUS

// Diference: Counterfactual - Observed

gen dif = predfull - predred if month > 89

// Our results should come from

egen meandif = mean(dif), by(month)

egen p2_5dif = pctile(dif), by(month) p(2.5)

egen p97_5dif = pctile(dif), by(month) p(97.5)

egen p_dif = mean(dif <= 0 ) if dif < ., by(month)

replace p_dif = 2*cond(p_dif < (1 - p_dif), p_dif, 1 - p_dif) if p_dif < .

egen firstobs = tag(month)

format %4.3f p_dif

format %4.1f meandif p2_5dif p97_5dif

list month meandif p2_5dif p97_5dif p_dif if firstobs == 1 & month > 89, clean noobs

twoway line meandif p2_5dif p97_5dif month if firstobs == 1 & month > 89, sort ///

ylab(-200(50)400) ytitle("Counts Diference:" "Counterfactual - Observed") ///

xlab(90 "Jun-2014" ///

97 "Jan-2015" ///

103 "Jul-2015" ///

109 "Jan-2016" ///

115 "Jul-2016" ///

121 "Jan-2017" ///

)xtitle("Calendar Time (months)") ///

legend(order(1 "Mean" 2 "95%CI LB" 3 "95%CI UB")) name("dif", replace)

graph export meandif.png, width(3000) replace

// Cumulative Diference: Counterfactual - Observed

drop if month < 90

sort id month

by id: gen cumdif = dif if _n == 1

by id: replace cumdif = cumdif[_n-1] + dif if _n > 1

egen meancumdif = mean(cumdif), by(month)

egen p2_5cumdif = pctile(cumdif), by(month) p(2.5)

egen p97_5cumdif = pctile(cumdif), by(month) p(97.5)

egen p_cumdif = mean(cumdif <= 0 ) if cumdif < ., by(month)

replace p_cumdif = 2*cond(p_cumdif < (1 - p_cumdif), p_cumdif, 1 - p_cumdif) if p_cumdif < .

gen totmeancumdif=meancumdif*(379)

gen totp2_5cumdif=p2_5cumdif*(379)

gen totp97_5cumdif=p97_5cumdif*(379)

// egen firstobs = tag(month)

format %4.3f p_cumdif

format %4.1f meancumdif p2_5cumdif p97_5cumdif

list month meancumdif p2_5cumdif p97_5cumdif p_cumdif if firstobs == 1 & month > 89, clean noobs

list month totmeancumdif totp2_5cumdif totp97_5cumdif p_cumdif if firstobs == 1 & month > 89, clean noobs

twoway line meancumdif p2_5cumdif p97_5cumdif month if firstobs == 1 & month > 89, sort ///

/// ylab(-200(50)400) ///

ytitle("Counts Cummulative Diference:" "Counterfactual - Observed") ///

xlab(90 "Jun-2014" ///

97 "Jan-2015" ///

103 "Jul-2015" ///

109 "Jan-2016" ///

115 "Jul-2016" ///

121 "Jan-2017" ///

)xtitle("Calendar Time (months)") ///

legend(order(1 "Mean" 2 "95%CI LB" 3 "95%CI UB")) name("cumdif", replace)

graph export meancumdif.png, width(3000) replace

*********************

**** BCG *****

*********************

*** MAKING PREDICTIONS

set scheme plotplain

clear

use analysisdataset

**BCG DOSES GIVEN XTMIXED NO EBOLA

xtmixed bcgdosesgiven time1-time4 population Feb Mar Apr May Jun July Aug Sep Oct Nov Dec ///

|| cluster:time1-time4 if bcgexclude==0 & bcgdosesoutlier!=1 & allmisbcg==0 & month<=89 ///

, residuals(ar, t(month))

preserve

// The betas from the fixed part comes from multivariate normal dist of B means and V covariances

// For reduced model

clear

est use bcg_reduc_file

mat b = e(b)

matselrc b B, col(1/17) // Extract the fixed part betas

mat v = e(V)

matselrc v V, col(1/17) row(1/17) // Extract the fixed part covariances

local nam = ""

forvalues i = 1/17 {

local nam = "`nam' red`i'"

}

// di "`nam'"

drawnorm `nam', cov(V) mean(B) n(1000) seed(846031265)

gen id = _n

save simbetas_reduced, replace

// For Full model

clear

est use bcg_full_file

mat b = e(b)

matselrc b B, col(1/23) // Extract the fixed part betas

mat v = e(V)

matselrc v V, col(1/23) row(1/23) // Extract the fixed part covariances

local nam = ""

forvalues i = 1/23 {

local nam = "`nam' ful`i'"

}

drawnorm `nam', cov(V) mean(B) n(1000) seed(846031265)

gen id = _n

save simbetas_full, replace

merge 1:1 id using simbetas_reduced

drop _merge

save simbetas, replace

restore

// Now lets predict from each month

gen one = 1

collapse (sum) one (p75) population , by(month time1-time7 /// population

Feb Mar Apr May Jun July Aug Sep Oct ///

Nov Dec timeafterebola1 timeafterebola2 timeafternoebola)

drop one

// We need 1000 predictions per month

expand 1000

bysort month: gen id = _n

order id month

sort id month

// Merge to our simulated betas

merge m:1 id using simbetas

gen predfull = time1 * ful1 + ///

time2 * ful2 + ///

time3 * ful3 + ///

time4 * ful4 + ///

time5 * ful5 + ///

time6 * ful6 + ///

time7 * ful7 + ///

population * ful8 + /// population coeficient

Feb * ful9 + ///

Mar * ful10 + ///

Apr * ful11 + ///

May * ful12 + ///

Jun * ful13 + ///

July * ful14 + ///

Aug * ful15 + ///

Sep * ful16 + ///

Oct * ful17 + ///

Nov * ful18 + ///

Dec * ful19 + ///

timeafterebola1 * ful20 + ///

timeafterebola2 * ful21 + ///

timeafternoebola * ful22 + ///

ful23 // The intercept

gen predred = time1 * red1 + ///

time2 * red2 + ///

time3 * red3 + ///

time4 * red4 + ///

population * red5 + /// population coeficient

Feb * red6 + ///

Mar * red7 + ///

Apr * red8 + ///

May * red9 + ///

Jun * red10 + ///

July * red11 + ///

Aug * red12 + ///

Sep * red13 + ///

Oct * red14 + ///

Nov * red15 + ///

Dec * red16 + ///

red17 // The intercept

************ OUR PRECIOUS

// Diference: Counterfactual - Observed

gen dif = predfull - predred if month > 89

// Our results should come from

egen meandif = mean(dif), by(month)

egen p2_5dif = pctile(dif), by(month) p(2.5)

egen p97_5dif = pctile(dif), by(month) p(97.5)

egen p_dif = mean(dif <= 0 ) if dif < ., by(month)

replace p_dif = 2*cond(p_dif < (1 - p_dif), p_dif, 1 - p_dif) if p_dif < .

egen firstobs = tag(month)

format %4.3f p_dif

format %4.1f meandif p2_5dif p97_5dif

list month meandif p2_5dif p97_5dif p_dif if firstobs == 1 & month > 89, clean noobs

twoway line meandif p2_5dif p97_5dif month if firstobs == 1 & month > 89, sort ///

ylab(-200(50)400) ytitle("Counts Diference:" "Counterfactual - Observed") ///

xlab(90 "Jun-2014" ///

97 "Jan-2015" ///

103 "Jul-2015" ///

109 "Jan-2016" ///

115 "Jul-2016" ///

121 "Jan-2017" ///

)xtitle("Calendar Time (months)") ///

legend(order(1 "Mean" 2 "95%CI LB" 3 "95%CI UB")) name("dif", replace)

graph export meandif.png, width(3000) replace

// Cumulative Diference: Counterfactual - Observed

drop if month < 90

sort id month

by id: gen cumdif = dif if _n == 1

by id: replace cumdif = cumdif[_n-1] + dif if _n > 1

egen meancumdif = mean(cumdif), by(month)

egen p2_5cumdif = pctile(cumdif), by(month) p(2.5)

egen p97_5cumdif = pctile(cumdif), by(month) p(97.5)

egen p_cumdif = mean(cumdif <= 0 ) if cumdif < ., by(month)

replace p_cumdif = 2*cond(p_cumdif < (1 - p_cumdif), p_cumdif, 1 - p_cumdif) if p_cumdif < .

gen totmeancumdif=meancumdif*(319)

gen totp2_5cumdif=p2_5cumdif*(319)

gen totp97_5cumdif=p97_5cumdif*(319)

// egen firstobs = tag(month)

format %4.3f p_cumdif

format %4.1f meancumdif p2_5cumdif p97_5cumdif

list month meancumdif p2_5cumdif p97_5cumdif p_cumdif if firstobs == 1 & month > 89, clean noobs

list month totmeancumdif totp2_5cumdif totp97_5cumdif p_cumdif if firstobs == 1 & month > 89, clean noobs

twoway line meancumdif p2_5cumdif p97_5cumdif month if firstobs == 1 & month > 89, sort ///

/// ylab(-200(50)400) ///

ytitle("Counts Cummulative Diference:" "Counterfactual - Observed") ///

xlab(90 "Jun-2014" ///

97 "Jan-2015" ///

103 "Jul-2015" ///

109 "Jan-2016" ///

115 "Jul-2016" ///

121 "Jan-2017" ///

)xtitle("Calendar Time (months)") ///

legend(order(1 "Mean" 2 "95%CI LB" 3 "95%CI UB")) name("cumdif", replace)

graph export meancumdif.png, width(3000) replace

*********************

**** Measles *****

*********************

*** MAKING PREDICTIONS

set scheme plotplain

clear

use analysisdataset

**MEASLES DOSES XTMIXED NO EBOLA

xtmixed measlesdosesgiven time1-time4 population Feb Mar Apr May Jun July Aug Sep Oct Nov Dec ///

|| cluster:time1-time4 if measlesexclude==0 & allmismeasles==0 & month<=89, residuals(ar, t(month))

preserve

// The betas from the fixed part comes from multivariate normal dist of B means and V covariances

// For reduced model

clear

est use measles_reduc_file

mat b = e(b)

matselrc b B, col(1/17) // Extract the fixed part betas

mat v = e(V)

matselrc v V, col(1/17) row(1/17) // Extract the fixed part covariances

local nam = ""

forvalues i = 1/17 {

local nam = "`nam' red`i'"

}

// di "`nam'"

drawnorm `nam', cov(V) mean(B) n(1000) seed(846031265)

gen id = _n

save simbetas_reduced, replace

// For Full model

clear

est use measles_full_file

mat b = e(b)

matselrc b B, col(1/23) // Extract the fixed part betas

mat v = e(V)

matselrc v V, col(1/23) row(1/23) // Extract the fixed part covariances

local nam = ""

forvalues i = 1/23 {

local nam = "`nam' ful`i'"

}

drawnorm `nam', cov(V) mean(B) n(1000) seed(846031265)

gen id = _n

save simbetas_full, replace

merge 1:1 id using simbetas_reduced

drop _merge

save simbetas, replace

restore

// Now lets predict from each month

gen one = 1

collapse (sum) one (p75) population , by(month time1-time7 /// population

Feb Mar Apr May Jun July Aug Sep Oct ///

Nov Dec timeafterebola1 timeafterebola2 timeafternoebola)

drop one

// We need 1000 predictions per month

expand 1000

bysort month: gen id = _n

order id month

sort id month

// Merge to our simulated betas

merge m:1 id using simbetas

gen predfull = time1 * ful1 + ///

time2 * ful2 + ///

time3 * ful3 + ///

time4 * ful4 + ///

time5 * ful5 + ///

time6 * ful6 + ///

time7 * ful7 + ///

population * ful8 + /// population coeficient

Feb * ful9 + ///

Mar * ful10 + ///

Apr * ful11 + ///

May * ful12 + ///

Jun * ful13 + ///

July * ful14 + ///

Aug * ful15 + ///

Sep * ful16 + ///

Oct * ful17 + ///

Nov * ful18 + ///

Dec * ful19 + ///

timeafterebola1 * ful20 + ///

timeafterebola2 * ful21 + ///

timeafternoebola * ful22 + ///

ful23 // The intercept

gen predred = time1 * red1 + ///

time2 * red2 + ///

time3 * red3 + ///

time4 * red4 + ///

population * red5 + /// population coeficient

Feb * red6 + ///

Mar * red7 + ///

Apr * red8 + ///

May * red9 + ///

Jun * red10 + ///

July * red11 + ///

Aug * red12 + ///

Sep * red13 + ///

Oct * red14 + ///

Nov * red15 + ///

Dec * red16 + ///

red17 // The intercept

************ OUR PRECIOUS

// Diference: Counterfactual - Observed

gen dif = predfull - predred if month > 89

// Our results should come from

egen meandif = mean(dif), by(month)

egen p2_5dif = pctile(dif), by(month) p(2.5)

egen p97_5dif = pctile(dif), by(month) p(97.5)

egen p_dif = mean(dif <= 0 ) if dif < ., by(month)

replace p_dif = 2*cond(p_dif < (1 - p_dif), p_dif, 1 - p_dif) if p_dif < .

egen firstobs = tag(month)

format %4.3f p_dif

format %4.1f meandif p2_5dif p97_5dif

list month meandif p2_5dif p97_5dif p_dif if firstobs == 1 & month > 89, clean noobs

twoway line meandif p2_5dif p97_5dif month if firstobs == 1 & month > 89, sort ///

ylab(-200(50)400) ytitle("Counts Diference:" "Counterfactual - Observed") ///

xlab(90 "Jun-2014" ///

97 "Jan-2015" ///

103 "Jul-2015" ///

109 "Jan-2016" ///

115 "Jul-2016" ///

121 "Jan-2017" ///

)xtitle("Calendar Time (months)") ///

legend(order(1 "Mean" 2 "95%CI LB" 3 "95%CI UB")) name("dif", replace)

graph export meandif.png, width(3000) replace

// Cumulative Diference: Counterfactual - Observed

drop if month < 90

sort id month

by id: gen cumdif = dif if _n == 1

by id: replace cumdif = cumdif[_n-1] + dif if _n > 1

egen meancumdif = mean(cumdif), by(month)

egen p2_5cumdif = pctile(cumdif), by(month) p(2.5)

egen p97_5cumdif = pctile(cumdif), by(month) p(97.5)

egen p_cumdif = mean(cumdif <= 0 ) if cumdif < ., by(month)

replace p_cumdif = 2*cond(p_cumdif < (1 - p_cumdif), p_cumdif, 1 - p_cumdif) if p_cumdif < .

gen totmeancumdif=meancumdif*(319)

gen totp2_5cumdif=p2_5cumdif*(319)

gen totp97_5cumdif=p97_5cumdif*(319)

// egen firstobs = tag(month)

format %4.3f p_cumdif

format %4.1f meancumdif p2_5cumdif p97_5cumdif

list month meancumdif p2_5cumdif p97_5cumdif p_cumdif if firstobs == 1 & month > 89, clean noobs

list month totmeancumdif totp2_5cumdif totp97_5cumdif p_cumdif if firstobs == 1 & month > 89, clean noobs

twoway line meancumdif p2_5cumdif p97_5cumdif month if firstobs == 1 & month > 89, sort ///

/// ylab(-200(50)400) ///

ytitle("Counts Cummulative Diference:" "Counterfactual - Observed") ///

xlab(90 "Jun-2014" ///

97 "Jan-2015" ///

103 "Jul-2015" ///

109 "Jan-2016" ///

115 "Jul-2016" ///

121 "Jan-2017" ///

)xtitle("Calendar Time (months)") ///

legend(order(1 "Mean" 2 "95%CI LB" 3 "95%CI UB")) name("cumdif", replace)

graph export meancumdif.png, width(3000) replace

*********************

**** Pentavalent *****

*********************

*** MAKING PREDICTIONS

set scheme plotplain

clear

use analysisdataset

*testing NO EBOLA

xtmixed pentavalent1given time1-time4 population Feb Mar Apr May Jun July Aug Sep Oct Nov Dec ///

|| cluster:time1-time4 if month<=89 & pentaexclude==0 & pentadosesoutlier!=1 & allmispenta==0, ///

residuals(ar, t(month))

preserve

// The betas from the fixed part comes from multivariate normal dist of B means and V covariances

// For reduced model

clear

est use penta_reduc_file

mat b = e(b)

matselrc b B, col(1/17) // Extract the fixed part betas

mat v = e(V)

matselrc v V, col(1/17) row(1/17) // Extract the fixed part covariances

local nam = ""

forvalues i = 1/17 {

local nam = "`nam' red`i'"

}

// di "`nam'"

drawnorm `nam', cov(V) mean(B) n(1000) seed(846031265)

gen id = _n

save simbetas_reduced, replace

// For Full model

clear

est use penta_full_file

mat b = e(b)

matselrc b B, col(1/23) // Extract the fixed part betas

mat v = e(V)

matselrc v V, col(1/23) row(1/23) // Extract the fixed part covariances

local nam = ""

forvalues i = 1/23 {

local nam = "`nam' ful`i'"

}

drawnorm `nam', cov(V) mean(B) n(1000) seed(846031265)

gen id = _n

save simbetas_full, replace

merge 1:1 id using simbetas_reduced

drop _merge

save simbetas, replace

restore

// Now lets predict from each month

gen one = 1

collapse (sum) one (p75) population , by(month time1-time7 /// population

Feb Mar Apr May Jun July Aug Sep Oct ///

Nov Dec timeafterebola1 timeafterebola2 timeafternoebola)

drop one

// We need 1000 predictions per month

expand 1000

bysort month: gen id = _n

order id month

sort id month

// Merge to our simulated betas

merge m:1 id using simbetas

gen predfull = time1 * ful1 + ///

time2 * ful2 + ///

time3 * ful3 + ///

time4 * ful4 + ///

time5 * ful5 + ///

time6 * ful6 + ///

time7 * ful7 + ///

population * ful8 + /// population coeficient

Feb * ful9 + ///

Mar * ful10 + ///

Apr * ful11 + ///

May * ful12 + ///

Jun * ful13 + ///

July * ful14 + ///

Aug * ful15 + ///

Sep * ful16 + ///

Oct * ful17 + ///

Nov * ful18 + ///

Dec * ful19 + ///

timeafterebola1 * ful20 + ///

timeafterebola2 * ful21 + ///

timeafternoebola * ful22 + ///

ful23 // The intercept

gen predred = time1 * red1 + ///

time2 * red2 + ///

time3 * red3 + ///

time4 * red4 + ///

population * red5 + /// population coeficient

Feb * red6 + ///

Mar * red7 + ///

Apr * red8 + ///

May * red9 + ///

Jun * red10 + ///

July * red11 + ///

Aug * red12 + ///

Sep * red13 + ///

Oct * red14 + ///

Nov * red15 + ///

Dec * red16 + ///

red17 // The intercept

************ OUR PRECIOUS

// Diference: Counterfactual - Observed

gen dif = predfull - predred if month > 89

// Our results should come from

egen meandif = mean(dif), by(month)

egen p2_5dif = pctile(dif), by(month) p(2.5)

egen p97_5dif = pctile(dif), by(month) p(97.5)

egen p_dif = mean(dif <= 0 ) if dif < ., by(month)

replace p_dif = 2*cond(p_dif < (1 - p_dif), p_dif, 1 - p_dif) if p_dif < .

egen firstobs = tag(month)

format %4.3f p_dif

format %4.1f meandif p2_5dif p97_5dif

list month meandif p2_5dif p97_5dif p_dif if firstobs == 1 & month > 89, clean noobs

twoway line meandif p2_5dif p97_5dif month if firstobs == 1 & month > 89, sort ///

ylab(-200(50)400) ytitle("Counts Diference:" "Counterfactual - Observed") ///

xlab(90 "Jun-2014" ///

97 "Jan-2015" ///

103 "Jul-2015" ///

109 "Jan-2016" ///

115 "Jul-2016" ///

121 "Jan-2017" ///

)xtitle("Calendar Time (months)") ///

legend(order(1 "Mean" 2 "95%CI LB" 3 "95%CI UB")) name("dif", replace)

graph export meandif.png, width(3000) replace

// Cumulative Diference: Counterfactual - Observed

drop if month < 90

sort id month

by id: gen cumdif = dif if _n == 1

by id: replace cumdif = cumdif[_n-1] + dif if _n > 1

egen meancumdif = mean(cumdif), by(month)

egen p2_5cumdif = pctile(cumdif), by(month) p(2.5)

egen p97_5cumdif = pctile(cumdif), by(month) p(97.5)

egen p_cumdif = mean(cumdif <= 0 ) if cumdif < ., by(month)

replace p_cumdif = 2*cond(p_cumdif < (1 - p_cumdif), p_cumdif, 1 - p_cumdif) if p_cumdif < .

gen totmeancumdif=meancumdif*(319)

gen totp2_5cumdif=p2_5cumdif*(319)

gen totp97_5cumdif=p97_5cumdif*(319)

// egen firstobs = tag(month)

format %4.3f p_cumdif

format %4.1f meancumdif p2_5cumdif p97_5cumdif

list month meancumdif p2_5cumdif p97_5cumdif p_cumdif if firstobs == 1 & month > 89, clean noobs

list month totmeancumdif totp2_5cumdif totp97_5cumdif p_cumdif if firstobs == 1 & month > 89, clean noobs

twoway line meancumdif p2_5cumdif p97_5cumdif month if firstobs == 1 & month > 89, sort ///

/// ylab(-200(50)400) ///

ytitle("Counts Cummulative Diference:" "Counterfactual - Observed") ///

xlab(90 "Jun-2014" ///

97 "Jan-2015" ///

103 "Jul-2015" ///

109 "Jan-2016" ///

115 "Jul-2016" ///

121 "Jan-2017" ///

)xtitle("Calendar Time (months)") ///

legend(order(1 "Mean" 2 "95%CI LB" 3 "95%CI UB")) name("cumdif", replace)

graph export meancumdif.png, width(3000) replace

*********************

**** 1st ANC visits *****

*********************

*** PREDICTIONS

set scheme plotplain

clear

use analysisdataset

*TESTING ANC1 NO EBOLA

xtmixed stancvisit time1-time4 population Feb Mar Apr May Jun July Aug Sep Oct Nov Dec ///

|| cluster:time1-time4 if antenatalexclude==0 & antenataldosesoutlier!=1 & allmisantenatal==0 & month<=89, ///

residuals(ar, t(month))

preserve

// The betas from the fixed part comes from multivariate normal dist of B means and V covariances

// For reduced model

clear

est use ANC_reduc_file

mat b = e(b)

matselrc b B, col(1/17) // Extract the fixed part betas

mat v = e(V)

matselrc v V, col(1/17) row(1/17) // Extract the fixed part covariances

local nam = ""

forvalues i = 1/17 {

local nam = "`nam' red`i'"

}

// di "`nam'"

drawnorm `nam', cov(V) mean(B) n(1000) seed(846031265)

gen id = _n

save simbetas_reduced, replace

// For Full model

clear

est use ANC_full_file

mat b = e(b)

matselrc b B, col(1/23) // Extract the fixed part betas

mat v = e(V)

matselrc v V, col(1/23) row(1/23) // Extract the fixed part covariances

local nam = ""

forvalues i = 1/23 {

local nam = "`nam' ful`i'"

}

drawnorm `nam', cov(V) mean(B) n(1000) seed(846031265)

gen id = _n

save simbetas_full, replace

merge 1:1 id using simbetas_reduced

drop _merge

save simbetas, replace

restore

// Now lets predict from each month

gen one = 1

collapse (sum) one (p75) population , by(month time1-time7 /// population

Feb Mar Apr May Jun July Aug Sep Oct ///

Nov Dec timeafterebola1 timeafterebola2 timeafternoebola)

drop one

// We need 1000 predictions per month

expand 1000

bysort month: gen id = _n

order id month

sort id month

// Merge to our simulated betas

merge m:1 id using simbetas

gen predfull = time1 * ful1 + ///

time2 * ful2 + ///

time3 * ful3 + ///

time4 * ful4 + ///

time5 * ful5 + ///

time6 * ful6 + ///

time7 * ful7 + ///

population * ful8 + /// population coeficient

Feb * ful9 + ///

Mar * ful10 + ///

Apr * ful11 + ///

May * ful12 + ///

Jun * ful13 + ///

July * ful14 + ///

Aug * ful15 + ///

Sep * ful16 + ///

Oct * ful17 + ///

Nov * ful18 + ///

Dec * ful19 + ///

timeafterebola1 * ful20 + ///

timeafterebola2 * ful21 + ///

timeafternoebola * ful22 + ///

ful23 // The intercept

gen predred = time1 * red1 + ///

time2 * red2 + ///

time3 * red3 + ///

time4 * red4 + ///

population * red5 + /// population coeficient

Feb * red6 + ///

Mar * red7 + ///

Apr * red8 + ///

May * red9 + ///

Jun * red10 + ///

July * red11 + ///

Aug * red12 + ///

Sep * red13 + ///

Oct * red14 + ///

Nov * red15 + ///

Dec * red16 + ///

red17 // The intercept

************ OUR PRECIOUS

// Diference: Counterfactual - Observed

gen dif = predfull - predred if month > 89

// Our results should come from

egen meandif = mean(dif), by(month)

egen p2_5dif = pctile(dif), by(month) p(2.5)

egen p97_5dif = pctile(dif), by(month) p(97.5)

egen p_dif = mean(dif <= 0 ) if dif < ., by(month)

replace p_dif = 2*cond(p_dif < (1 - p_dif), p_dif, 1 - p_dif) if p_dif < .

egen firstobs = tag(month)

format %4.3f p_dif

format %4.1f meandif p2_5dif p97_5dif

list month predfull predred meandif p2_5dif p97_5dif p_dif if firstobs == 1 & month > 89, clean noobs

twoway line meandif p2_5dif p97_5dif month if firstobs == 1 & month > 89, sort ///

ylab(-200(50)400) ytitle("Counts Diference:" "Counterfactual - Observed") ///

xlab(90 "Jun-2014" ///

97 "Jan-2015" ///

103 "Jul-2015" ///

109 "Jan-2016" ///

115 "Jul-2016" ///

121 "Jan-2017" ///

)xtitle("Calendar Time (months)") ///

legend(order(1 "Mean" 2 "95%CI LB" 3 "95%CI UB")) name("dif", replace)

graph export meandif.png, width(3000) replace

// Cumulative Diference: Counterfactual - Observed

drop if month < 90

sort id month

by id: gen cumdif = dif if _n == 1

by id: replace cumdif = cumdif[_n-1] + dif if _n > 1

egen meancumdif = mean(cumdif), by(month)

egen p2_5cumdif = pctile(cumdif), by(month) p(2.5)

egen p97_5cumdif = pctile(cumdif), by(month) p(97.5)

egen p_cumdif = mean(cumdif <= 0 ) if cumdif < ., by(month)

replace p_cumdif = 2*cond(p_cumdif < (1 - p_cumdif), p_cumdif, 1 - p_cumdif) if p_cumdif < .

gen totmeancumdif=meancumdif*(276)

gen totp2_5cumdif=p2_5cumdif*(276)

gen totp97_5cumdif=p97_5cumdif*(276)

// egen firstobs = tag(month)

format %4.3f p_cumdif

format %4.1f meancumdif p2_5cumdif p97_5cumdif

list month meancumdif p2_5cumdif p97_5cumdif p_cumdif if firstobs == 1 & month > 89, clean noobs

list month totmeancumdif totp2_5cumdif totp97_5cumdif p_cumdif if firstobs == 1 & month > 89, clean noobs

twoway line meancumdif p2_5cumdif p97_5cumdif month if firstobs == 1 & month > 89, sort ///

/// ylab(-200(50)400) ///

ytitle("Counts Cummulative Diference:" "Counterfactual - Observed") ///

xlab(90 "Jun-2014" ///

97 "Jan-2015" ///

103 "Jul-2015" ///

109 "Jan-2016" ///

115 "Jul-2016" ///

121 "Jan-2017" ///

)xtitle("Calendar Time (months)") ///

legend(order(1 "Mean" 2 "95%CI LB" 3 "95%CI UB")) name("cumdif", replace)

graph export meancumdif.png, width(3000) replace

*********************

**** Institutional Births *****

*********************

*** PREDICTIONS

set scheme plotplain

clear

use analysisdataset

** Skilled delivery facility no ebola

xtmixed skilleddelfacility time1-time4 population Feb Mar Apr May Jun July Aug Sep Oct Nov Dec ///

|| cluster:time1-time4 if deliveryexclude==0 & deliverydosesoutlier!=1 & allmisdelivery==0 & month<=89, ///

residuals(ar, t(month))

preserve

// The betas from the fixed part comes from multivariate normal dist of B means and V covariances

// For reduced model

clear

est use delivery_reduc_file

mat b = e(b)

matselrc b B, col(1/17) // Extract the fixed part betas

mat v = e(V)

matselrc v V, col(1/17) row(1/17) // Extract the fixed part covariances

local nam = ""

forvalues i = 1/17 {

local nam = "`nam' red`i'"

}

// di "`nam'"

drawnorm `nam', cov(V) mean(B) n(1000) seed(846031265)

gen id = _n

save simbetas_reduced, replace

// For Full model

clear

est use delivery_full_file

mat b = e(b)

matselrc b B, col(1/23) // Extract the fixed part betas

mat v = e(V)

matselrc v V, col(1/23) row(1/23) // Extract the fixed part covariances

local nam = ""

forvalues i = 1/23 {

local nam = "`nam' ful`i'"

}

drawnorm `nam', cov(V) mean(B) n(1000) seed(846031265)

gen id = _n

save simbetas_full, replace

merge 1:1 id using simbetas_reduced

drop _merge

save simbetas, replace

restore

// Now lets predict from each month

gen one = 1

collapse (sum) one (p75) population , by(month time1-time7 /// population

Feb Mar Apr May Jun July Aug Sep Oct ///

Nov Dec timeafterebola1 timeafterebola2 timeafternoebola)

drop one

// We need 1000 predictions per month

expand 1000

bysort month: gen id = _n

order id month

sort id month

// Merge to our simulated betas

merge m:1 id using simbetas

gen predfull = time1 * ful1 + ///

time2 * ful2 + ///

time3 * ful3 + ///

time4 * ful4 + ///

time5 * ful5 + ///

time6 * ful6 + ///

time7 * ful7 + ///

population * ful8 + /// population coeficient

Feb * ful9 + ///

Mar * ful10 + ///

Apr * ful11 + ///

May * ful12 + ///

Jun * ful13 + ///

July * ful14 + ///

Aug * ful15 + ///

Sep * ful16 + ///

Oct * ful17 + ///

Nov * ful18 + ///

Dec * ful19 + ///

timeafterebola1 * ful20 + ///

timeafterebola2 * ful21 + ///

timeafternoebola * ful22 + ///

ful23 // The intercept

gen predred = time1 * red1 + ///

time2 * red2 + ///

time3 * red3 + ///

time4 * red4 + ///

population * red5 + /// population coeficient

Feb * red6 + ///

Mar * red7 + ///

Apr * red8 + ///

May * red9 + ///

Jun * red10 + ///

July * red11 + ///

Aug * red12 + ///

Sep * red13 + ///

Oct * red14 + ///

Nov * red15 + ///

Dec * red16 + ///

red17 // The intercept

************ OUR PRECIOUS

// Diference: Counterfactual - Observed

gen dif = predfull - predred if month > 89

// Our results should come from

egen meandif = mean(dif), by(month)

egen p2_5dif = pctile(dif), by(month) p(2.5)

egen p97_5dif = pctile(dif), by(month) p(97.5)

egen p_dif = mean(dif <= 0 ) if dif < ., by(month)

replace p_dif = 2*cond(p_dif < (1 - p_dif), p_dif, 1 - p_dif) if p_dif < .

egen firstobs = tag(month)

format %4.3f p_dif

format %4.1f meandif p2_5dif p97_5dif

list month meandif p2_5dif p97_5dif p_dif if firstobs == 1 & month > 89, clean noobs

twoway line meandif p2_5dif p97_5dif month if firstobs == 1 & month > 89, sort ///

ylab(-200(50)400) ytitle("Counts Diference:" "Counterfactual - Observed") ///

xlab(90 "Jun-2014" ///

97 "Jan-2015" ///

103 "Jul-2015" ///

109 "Jan-2016" ///

115 "Jul-2016" ///

121 "Jan-2017" ///

)xtitle("Calendar Time (months)") ///

legend(order(1 "Mean" 2 "95%CI LB" 3 "95%CI UB")) name("dif", replace)

graph export meandif.png, width(3000) replace

// Cumulative Diference: Counterfactual - Observed

drop if month < 90

sort id month

by id: gen cumdif = dif if _n == 1

by id: replace cumdif = cumdif[_n-1] + dif if _n > 1

egen meancumdif = mean(cumdif), by(month)

egen p2_5cumdif = pctile(cumdif), by(month) p(2.5)

egen p97_5cumdif = pctile(cumdif), by(month) p(97.5)

egen p_cumdif = mean(cumdif <= 0 ) if cumdif < ., by(month)

replace p_cumdif = 2*cond(p_cumdif < (1 - p_cumdif), p_cumdif, 1 - p_cumdif) if p_cumdif < .

gen totmeancumdif=meancumdif*(275)

gen totp2_5cumdif=p2_5cumdif*(275)

gen totp97_5cumdif=p97_5cumdif*(275)

// egen firstobs = tag(month)

format %4.3f p_cumdif

format %4.1f meancumdif p2_5cumdif p97_5cumdif

list month meancumdif p2_5cumdif p97_5cumdif p_cumdif if firstobs == 1 & month > 89, clean noobs

list month totmeancumdif totp2_5cumdif totp97_5cumdif p_cumdif if firstobs == 1 & month > 89, clean noobs

twoway line meancumdif p2_5cumdif p97_5cumdif month if firstobs == 1 & month > 89, sort ///

/// ylab(-200(50)400) ///

ytitle("Counts Cummulative Diference:" "Counterfactual - Observed") ///

xlab(90 "Jun-2014" ///

97 "Jan-2015" ///

103 "Jul-2015" ///

109 "Jan-2016" ///

115 "Jul-2016" ///

121 "Jan-2017" ///

)xtitle("Calendar Time (months)") ///

legend(order(1 "Mean" 2 "95%CI LB" 3 "95%CI UB")) name("cumdif", replace)

graph export meancumdif.png, width(3000) replace

*********************

**** PNC within 6 weeks *****

*********************

*** PREDICTIONS

set scheme plotplain

clear

use analysisdataset

*testing *PNC NO EBOLA

xtmixed pncwithin6weeks time3-time4 population Feb Mar Apr May Jun July Aug Sep Oct Nov Dec ///

|| cluster:time3-time4 if month<=89 & pncexclude==0 & pncdosesoutlier!=1 & allmispnc==0 , ///

residuals(ar, t(month))

preserve

// The betas from the fixed part comes from multivariate normal dist of B means and V covariances

// For reduced model

clear

est use PNC_reduc_file

est replay

mat b = e(b)

matselrc b B, col(1/15) // Extract the fixed part betas

mat v = e(V)

matselrc v V, col(1/15) row(1/15) // Extract the fixed part covariances

local nam = ""

forvalues i = 1/15 {

local nam = "`nam' red`i'"

}

// di "`nam'"

drawnorm `nam', cov(V) mean(B) n(1000) seed(846031265)

gen id = _n

save simbetas_reduced, replace

// For Full model

clear

est use PNC_full_file

est replay

mat b = e(b)

matselrc b B, col(1/21) // Extract the fixed part betas

mat v = e(V)

matselrc v V, col(1/21) row(1/21) // Extract the fixed part covariances

local nam = ""

forvalues i = 1/21 {

local nam = "`nam' ful`i'"

}

drawnorm `nam', cov(V) mean(B) n(1000) seed(846031265)

gen id = _n

save simbetas_full, replace

merge 1:1 id using simbetas_reduced

drop _merge

save simbetas, replace

restore

// Now lets predict from each month

gen one = 1

collapse (sum) one (p75) population , by(month time1-time7 /// population

Feb Mar Apr May Jun July Aug Sep Oct ///

Nov Dec timeafterebola1 timeafterebola2 timeafternoebola)

drop one

// We need 1000 predictions per month

expand 1000

bysort month: gen id = _n

order id month

sort id month

// Merge to our simulated betas

merge m:1 id using simbetas

gen predfull = time3 * ful1 + ///

time4 * ful2 + ///

time5 * ful3 + ///

time6 * ful4 + ///

time7 * ful5 + ///

population * ful6 + /// population coeficient

Feb * ful7 + ///

Mar * ful8 + ///

Apr * ful9 + ///

May * ful10 + ///

Jun * ful11 + ///

July * ful12 + ///

Aug * ful13 + ///

Sep * ful14 + ///

Oct * ful15 + ///

Nov * ful16 + ///

Dec * ful17 + ///

timeafterebola1 * ful18 + ///

timeafterebola2 * ful19 + ///

timeafternoebola * ful20 + ///

ful21 // The intercept

gen predred = time3 * red1 + ///

time4 * red2 + ///

population * red3 + /// population coeficient

Feb * red4 + ///

Mar * red5 + ///

Apr * red6 + ///

May * red7 + ///

Jun * red8 + ///

July * red9 + ///

Aug * red10 + ///

Sep * red11 + ///

Oct * red12 + ///

Nov * red13 + ///

Dec * red14 + ///

red15 // The intercept

************ OUR PRECIOUS

// Diference: Counterfactual - Observed

gen dif = predfull - predred if month > 89

// Our results should come from

egen meandif = mean(dif), by(month)

egen p2_5dif = pctile(dif), by(month) p(2.5)

egen p97_5dif = pctile(dif), by(month) p(97.5)

egen p_dif = mean(dif <= 0 ) if dif < ., by(month)

replace p_dif = 2*cond(p_dif < (1 - p_dif), p_dif, 1 - p_dif) if p_dif < .

egen firstobs = tag(month)

format %4.3f p_dif

format %4.1f meandif p2_5dif p97_5dif

list month meandif p2_5dif p97_5dif p_dif if firstobs == 1 & month > 89, clean noobs

twoway line meandif p2_5dif p97_5dif month if firstobs == 1 & month > 89, sort ///

ylab(-200(50)400) ytitle("Counts Diference:" "Counterfactual - Observed") ///

xlab(90 "Jun-2014" ///

97 "Jan-2015" ///

103 "Jul-2015" ///

109 "Jan-2016" ///

115 "Jul-2016" ///

121 "Jan-2017" ///

)xtitle("Calendar Time (months)") ///

legend(order(1 "Mean" 2 "95%CI LB" 3 "95%CI UB")) name("dif", replace)

graph export meandif.png, width(3000) replace

// Cumulative Diference: Counterfactual - Observed

drop if month < 90

sort id month

by id: gen cumdif = dif if _n == 1

by id: replace cumdif = cumdif[_n-1] + dif if _n > 1

egen meancumdif = mean(cumdif), by(month)

egen p2_5cumdif = pctile(cumdif), by(month) p(2.5)

egen p97_5cumdif = pctile(cumdif), by(month) p(97.5)

egen p_cumdif = mean(cumdif <= 0 ) if cumdif < ., by(month)

replace p_cumdif = 2*cond(p_cumdif < (1 - p_cumdif), p_cumdif, 1 - p_cumdif) if p_cumdif < .

gen totmeancumdif=meancumdif*(274)

gen totp2_5cumdif=p2_5cumdif*(274)

gen totp97_5cumdif=p97_5cumdif*(274)

// egen firstobs = tag(month)

format %4.3f p_cumdif

format %9.1f meancumdif p2_5cumdif p97_5cumdif

// list month meancumdif p2_5cumdif p97_5cumdif p_cumdif if firstobs == 1 & month > 89, clean noobs

list month totmeancumdif totp2_5cumdif totp97_5cumdif p_cumdif if firstobs == 1 & month > 89, clean noobs abbr(10)

twoway line meancumdif p2_5cumdif p97_5cumdif month if firstobs == 1 & month > 89, sort ///

/// ylab(-200(50)400) ///

ytitle("Counts Cummulative Diference:" "Counterfactual - Observed") ///

xlab(90 "Jun-2014" ///

97 "Jan-2015" ///

103 "Jul-2015" ///

109 "Jan-2016" ///

115 "Jul-2016" ///

121 "Jan-2017" ///

)xtitle("Calendar Time (months)") ///

legend(order(1 "Mean" 2 "95%CI LB" 3 "95%CI UB")) name("cumdif", replace)

graph export meancumdif.png, width(3000) replace

*********************

**** ACT treatment *****

*********************

*** PREDICTIONS

set scheme plotplain

clear

use analysisdataset

** ACT no ebola

xtmixed acttreatment time1-time4 population Feb Mar Apr May Jun July Aug Sep Oct Nov Dec ///

|| cluster:time1-time4 if actexclude==0 & actdosesoutlier!=1 & allmisact==0 & month<=89, ///

residuals(ar, t(month))

preserve

// The betas from the fixed part comes from multivariate normal dist of B means and V covariances

// For reduced model

clear

est use ACT_reduc_file

mat b = e(b)

matselrc b B, col(1/17) // Extract the fixed part betas

mat v = e(V)

matselrc v V, col(1/17) row(1/17) // Extract the fixed part covariances

local nam = ""

forvalues i = 1/17 {

local nam = "`nam' red`i'"

}

// di "`nam'"

drawnorm `nam', cov(V) mean(B) n(1000) seed(846031265)

gen id = _n

save simbetas_reduced, replace

// For Full model

clear

est use ACT_full_file

mat b = e(b)

matselrc b B, col(1/23) // Extract the fixed part betas

mat v = e(V)

matselrc v V, col(1/23) row(1/23) // Extract the fixed part covariances

local nam = ""

forvalues i = 1/23 {

local nam = "`nam' ful`i'"

}

drawnorm `nam', cov(V) mean(B) n(1000) seed(846031265)

gen id = _n

save simbetas_full, replace

merge 1:1 id using simbetas_reduced

drop _merge

save simbetas, replace

restore

// Now lets predict from each month

gen one = 1

collapse (sum) one (p75) population , by(month time1-time7 /// population

Feb Mar Apr May Jun July Aug Sep Oct ///

Nov Dec timeafterebola1 timeafterebola2 timeafternoebola)

drop one

// We need 1000 predictions per month

expand 1000

bysort month: gen id = _n

order id month

sort id month

// Merge to our simulated betas

merge m:1 id using simbetas

gen predfull = time1 * ful1 + ///

time2 * ful2 + ///

time3 * ful3 + ///

time4 * ful4 + ///

time5 * ful5 + ///

time6 * ful6 + ///

time7 * ful7 + ///

population * ful8 + /// population coeficient

Feb * ful9 + ///

Mar * ful10 + ///

Apr * ful11 + ///

May * ful12 + ///

Jun * ful13 + ///

July * ful14 + ///

Aug * ful15 + ///

Sep * ful16 + ///

Oct * ful17 + ///

Nov * ful18 + ///

Dec * ful19 + ///

timeafterebola1 * ful20 + ///

timeafterebola2 * ful21 + ///

timeafternoebola * ful22 + ///

ful23 // The intercept

gen predred = time1 * red1 + ///

time2 * red2 + ///

time3 * red3 + ///

time4 * red4 + ///

population * red5 + /// population coeficient

Feb * red6 + ///

Mar * red7 + ///

Apr * red8 + ///

May * red9 + ///

Jun * red10 + ///

July * red11 + ///

Aug * red12 + ///

Sep * red13 + ///

Oct * red14 + ///

Nov * red15 + ///

Dec * red16 + ///

red17 // The intercept

************ OUR PRECIOUS

// Diference: Counterfactual - Observed

gen dif = predfull - predred if month > 89

// Our results should come from

egen meandif = mean(dif), by(month)

egen p2_5dif = pctile(dif), by(month) p(2.5)

egen p97_5dif = pctile(dif), by(month) p(97.5)

egen p_dif = mean(dif <= 0 ) if dif < ., by(month)

replace p_dif = 2*cond(p_dif < (1 - p_dif), p_dif, 1 - p_dif) if p_dif < .

egen firstobs = tag(month)

format %4.3f p_dif

format %4.1f meandif p2_5dif p97_5dif

list month meandif p2_5dif p97_5dif p_dif if firstobs == 1 & month > 89, clean noobs

twoway line meandif p2_5dif p97_5dif month if firstobs == 1 & month > 89, sort ///

ylab(-200(50)400) ytitle("Counts Diference:" "Counterfactual - Observed") ///

xlab(90 "Jun-2014" ///

97 "Jan-2015" ///

103 "Jul-2015" ///

109 "Jan-2016" ///

115 "Jul-2016" ///

121 "Jan-2017" ///

)xtitle("Calendar Time (months)") ///

legend(order(1 "Mean" 2 "95%CI LB" 3 "95%CI UB")) name("dif", replace)

graph export meandif.png, width(3000) replace

// Cumulative Diference: Counterfactual - Observed

drop if month < 90

sort id month

by id: gen cumdif = dif if _n == 1

by id: replace cumdif = cumdif[_n-1] + dif if _n > 1

egen meancumdif = mean(cumdif), by(month)

egen p2_5cumdif = pctile(cumdif), by(month) p(2.5)

egen p97_5cumdif = pctile(cumdif), by(month) p(97.5)

egen p_cumdif = mean(cumdif <= 0 ) if cumdif < ., by(month)

replace p_cumdif = 2*cond(p_cumdif < (1 - p_cumdif), p_cumdif, 1 - p_cumdif) if p_cumdif < .

gen totmeancumdif=meancumdif*(379)

gen totp2_5cumdif=p2_5cumdif*(379)

gen totp97_5cumdif=p97_5cumdif*(379)

// egen firstobs = tag(month)

format %4.3f p_cumdif

format %4.1f meancumdif p2_5cumdif p97_5cumdif

list month meancumdif p2_5cumdif p97_5cumdif p_cumdif if firstobs == 1 & month > 89, clean noobs

list month totmeancumdif totp2_5cumdif totp97_5cumdif p_cumdif if firstobs == 1 & month > 89, clean noobs

twoway line meancumdif p2_5cumdif p97_5cumdif month if firstobs == 1 & month > 89, sort ///

/// ylab(-200(50)400) ///

ytitle("Counts Cummulative Diference:" "Counterfactual - Observed") ///

xlab(90 "Jun-2014" ///

97 "Jan-2015" ///

103 "Jul-2015" ///

109 "Jan-2016" ///

115 "Jul-2016" ///

121 "Jan-2017" ///

)xtitle("Calendar Time (months)") ///

legend(order(1 "Mean" 2 "95%CI LB" 3 "95%CI UB")) name("cumdif", replace)

graph export meancumdif.png, width(3000) replace

*********************

**** ARI treatment *****

*********************

*** PREDICTIONS

set scheme plotplain

clear

use analysisdataset

** ARIs no ebola

xtmixed ari time3-time4 population Feb Mar Apr May Jun July Aug Sep Oct Nov Dec ///

|| cluster:time3-time4 if month<=89 & respiratoryexclude==0 & respiratorydosesoutlier!=1 ///

& allmisrespiratory==0, residuals(ar, t(month))

preserve

// The betas from the fixed part comes from multivariate normal dist of B means and V covariances

// For reduced model

clear

est use ARI_reduc_file

est replay

mat b = e(b)

matselrc b B, col(1/15) // Extract the fixed part betas

mat v = e(V)

matselrc v V, col(1/15) row(1/15) // Extract the fixed part covariances

local nam = ""

forvalues i = 1/15 {

local nam = "`nam' red`i'"

}

// di "`nam'"

drawnorm `nam', cov(V) mean(B) n(1000) seed(846031265)

gen id = _n

save simbetas_reduced, replace

// For Full model

clear

est use ARI_full_file

est replay

mat b = e(b)

matselrc b B, col(1/21) // Extract the fixed part betas

mat v = e(V)

matselrc v V, col(1/21) row(1/21) // Extract the fixed part covariances

local nam = ""

forvalues i = 1/21 {

local nam = "`nam' ful`i'"

}

drawnorm `nam', cov(V) mean(B) n(1000) seed(846031265)

gen id = _n

save simbetas_full, replace

merge 1:1 id using simbetas_reduced

drop _merge

save simbetas, replace

restore

// Now lets predict from each month

gen one = 1

collapse (sum) one (p75) population , by(month time1-time7 /// population

Feb Mar Apr May Jun July Aug Sep Oct ///

Nov Dec timeafterebola1 timeafterebola2 timeafternoebola)

drop one

// We need 1000 predictions per month

expand 1000

bysort month: gen id = _n

order id month

sort id month

// Merge to our simulated betas

merge m:1 id using simbetas

gen predfull = time3 * ful1 + ///

time4 * ful2 + ///

time5 * ful3 + ///

time6 * ful4 + ///

time7 * ful5 + ///

population * ful6 + /// population coeficient

Feb * ful7 + ///

Mar * ful8 + ///

Apr * ful9 + ///

May * ful10 + ///

Jun * ful11 + ///

July * ful12 + ///

Aug * ful13 + ///

Sep * ful14 + ///

Oct * ful15 + ///

Nov * ful16 + ///

Dec * ful17 + ///

timeafterebola1 * ful18 + ///

timeafterebola2 * ful19 + ///

timeafternoebola * ful20 + ///

ful21 // The intercept

gen predred = time3 * red1 + ///

time4 * red2 + ///

population * red3 + /// population coeficient

Feb * red4 + ///

Mar * red5 + ///

Apr * red6 + ///

May * red7 + ///

Jun * red8 + ///

July * red9 + ///

Aug * red10 + ///

Sep * red11 + ///

Oct * red12 + ///

Nov * red13 + ///

Dec * red14 + ///

red15 // The intercept

************ OUR PRECIOUS

// Diference: Counterfactual - Observed

gen dif = predfull - predred if month > 89

// Our results should come from

egen meandif = mean(dif), by(month)

egen p2_5dif = pctile(dif), by(month) p(2.5)

egen p97_5dif = pctile(dif), by(month) p(97.5)

egen p_dif = mean(dif <= 0 ) if dif < ., by(month)

replace p_dif = 2*cond(p_dif < (1 - p_dif), p_dif, 1 - p_dif) if p_dif < .

egen firstobs = tag(month)

format %4.3f p_dif

format %4.1f meandif p2_5dif p97_5dif

list month meandif p2_5dif p97_5dif p_dif if firstobs == 1 & month > 89, clean noobs

twoway line meandif p2_5dif p97_5dif month if firstobs == 1 & month > 89, sort ///

ylab(-200(50)400) ytitle("Counts Diference:" "Counterfactual - Observed") ///

xlab(90 "Jun-2014" ///

97 "Jan-2015" ///

103 "Jul-2015" ///

109 "Jan-2016" ///

115 "Jul-2016" ///

121 "Jan-2017" ///

)xtitle("Calendar Time (months)") ///

legend(order(1 "Mean" 2 "95%CI LB" 3 "95%CI UB")) name("dif", replace)

graph export meandif.png, width(3000) replace

// Cumulative Diference: Counterfactual - Observed

drop if month < 90

sort id month

by id: gen cumdif = dif if _n == 1

by id: replace cumdif = cumdif[_n-1] + dif if _n > 1

egen meancumdif = mean(cumdif), by(month)

egen p2_5cumdif = pctile(cumdif), by(month) p(2.5)

egen p97_5cumdif = pctile(cumdif), by(month) p(97.5)

egen p_cumdif = mean(cumdif <= 0 ) if cumdif < ., by(month)

replace p_cumdif = 2*cond(p_cumdif < (1 - p_cumdif), p_cumdif, 1 - p_cumdif) if p_cumdif < .

gen totmeancumdif=meancumdif*(244)

gen totp2_5cumdif=p2_5cumdif*(244)

gen totp97_5cumdif=p97_5cumdif*(244)

// egen firstobs = tag(month)

format %4.3f p_cumdif

format %9.1f meancumdif p2_5cumdif p97_5cumdif

// list month meancumdif p2_5cumdif p97_5cumdif p_cumdif if firstobs == 1 & month > 89, clean noobs

list month totmeancumdif totp2_5cumdif totp97_5cumdif p_cumdif if firstobs == 1 & month > 89, clean noobs abbr(10)

twoway line meancumdif p2_5cumdif p97_5cumdif month if firstobs == 1 & month > 89, sort ///

/// ylab(-200(50)400) ///

ytitle("Counts Cummulative Diference:" "Counterfactual - Observed") ///

xlab(90 "Jun-2014" ///

97 "Jan-2015" ///

103 "Jul-2015" ///

109 "Jan-2016" ///

115 "Jul-2016" ///

121 "Jan-2017" ///

)xtitle("Calendar Time (months)") ///

legend(order(1 "Mean" 2 "95%CI LB" 3 "95%CI UB")) name("cumdif", replace)

graph export meancumdif.png, width(3000) replace

*********************

**** Depo provera *****

*********************

**PREDICTIONS

set scheme plotplain

clear

use analysisdataset

*testing NO EBOLA DEPO

xtmixed depoprovera time1-time4 population Feb Mar Apr May Jun July Aug Sep Oct Nov Dec ///

|| cluster:time1-time4 if depoexclude==0 & depodosesoutlier!=1 & allmisdepo==0 & month<=89, ///

residuals(ar, t(month))

preserve

// The betas from the fixed part comes from multivariate normal dist of B means and V covariances

// For reduced model

clear

est use depo_reduc_file

mat b = e(b)

matselrc b B, col(1/17) // Extract the fixed part betas

mat v = e(V)

matselrc v V, col(1/17) row(1/17) // Extract the fixed part covariances

local nam = ""

forvalues i = 1/17 {

local nam = "`nam' red`i'"

}

// di "`nam'"

drawnorm `nam', cov(V) mean(B) n(1000) seed(846031265)

gen id = _n

save simbetas_reduced, replace

// For Full model

clear

est use depo_full_file

mat b = e(b)

matselrc b B, col(1/23) // Extract the fixed part betas

mat v = e(V)

matselrc v V, col(1/23) row(1/23) // Extract the fixed part covariances

local nam = ""

forvalues i = 1/23 {

local nam = "`nam' ful`i'"

}

drawnorm `nam', cov(V) mean(B) n(1000) seed(846031265)

gen id = _n

save simbetas_full, replace

merge 1:1 id using simbetas_reduced

drop _merge

save simbetas, replace

restore

// Now lets predict from each month

gen one = 1

collapse (sum) one (p75) population , by(month time1-time7 /// population

Feb Mar Apr May Jun July Aug Sep Oct ///

Nov Dec timeafterebola1 timeafterebola2 timeafternoebola)

drop one

// We need 1000 predictions per month

expand 1000

bysort month: gen id = _n

order id month

sort id month

// Merge to our simulated betas

merge m:1 id using simbetas

gen predfull = time1 * ful1 + ///

time2 * ful2 + ///

time3 * ful3 + ///

time4 * ful4 + ///

time5 * ful5 + ///

time6 * ful6 + ///

time7 * ful7 + ///

population * ful8 + /// population coeficient

Feb * ful9 + ///

Mar * ful10 + ///

Apr * ful11 + ///

May * ful12 + ///

Jun * ful13 + ///

July * ful14 + ///

Aug * ful15 + ///

Sep * ful16 + ///

Oct * ful17 + ///

Nov * ful18 + ///

Dec * ful19 + ///

timeafterebola1 * ful20 + ///

timeafterebola2 * ful21 + ///

timeafternoebola * ful22 + ///

ful23 // The intercept

gen predred = time1 * red1 + ///

time2 * red2 + ///

time3 * red3 + ///

time4 * red4 + ///

population * red5 + /// population coeficient

Feb * red6 + ///

Mar * red7 + ///

Apr * red8 + ///

May * red9 + ///

Jun * red10 + ///

July * red11 + ///

Aug * red12 + ///

Sep * red13 + ///

Oct * red14 + ///

Nov * red15 + ///

Dec * red16 + ///

red17 // The intercept

************ OUR PRECIOUS

// Diference: Counterfactual - Observed

gen dif = predfull - predred if month > 89

// Our results should come from

egen meandif = mean(dif), by(month)

egen p2_5dif = pctile(dif), by(month) p(2.5)

egen p97_5dif = pctile(dif), by(month) p(97.5)

egen p_dif = mean(dif <= 0 ) if dif < ., by(month)

replace p_dif = 2*cond(p_dif < (1 - p_dif), p_dif, 1 - p_dif) if p_dif < .

egen firstobs = tag(month)

format %4.3f p_dif

format %4.1f meandif p2_5dif p97_5dif

list month meandif p2_5dif p97_5dif p_dif if firstobs == 1 & month > 89, clean noobs

twoway line meandif p2_5dif p97_5dif month if firstobs == 1 & month > 89, sort ///

ylab(-200(50)400) ytitle("Counts Diference:" "Counterfactual - Observed") ///

xlab(90 "Jun-2014" ///

97 "Jan-2015" ///

103 "Jul-2015" ///

109 "Jan-2016" ///

115 "Jul-2016" ///

121 "Jan-2017" ///

)xtitle("Calendar Time (months)") ///

legend(order(1 "Mean" 2 "95%CI LB" 3 "95%CI UB")) name("dif", replace)

graph export meandif.png, width(3000) replace

// Cumulative Diference: Counterfactual - Observed

drop if month < 90

sort id month

by id: gen cumdif = dif if _n == 1

by id: replace cumdif = cumdif[_n-1] + dif if _n > 1

egen meancumdif = mean(cumdif), by(month)

egen p2_5cumdif = pctile(cumdif), by(month) p(2.5)

egen p97_5cumdif = pctile(cumdif), by(month) p(97.5)

egen p_cumdif = mean(cumdif <= 0 ) if cumdif < ., by(month)

replace p_cumdif = 2*cond(p_cumdif < (1 - p_cumdif), p_cumdif, 1 - p_cumdif) if p_cumdif < .

gen totmeancumdif=meancumdif*(272)

gen totp2_5cumdif=p2_5cumdif*(272)

gen totp97_5cumdif=p97_5cumdif*(272)

// egen firstobs = tag(month)

format %4.3f p_cumdif

format %4.1f meancumdif p2_5cumdif p97_5cumdif

list month meancumdif p2_5cumdif p97_5cumdif p_cumdif if firstobs == 1 & month > 89, clean noobs

list month totmeancumdif totp2_5cumdif totp97_5cumdif p_cumdif if firstobs == 1 & month > 89, clean noobs

twoway line meancumdif p2_5cumdif p97_5cumdif month if firstobs == 1 & month > 89, sort ///

/// ylab(-200(50)400) ///

ytitle("Counts Cummulative Diference:" "Counterfactual - Observed") ///

xlab(90 "Jun-2014" ///

97 "Jan-2015" ///

103 "Jul-2015" ///

109 "Jan-2016" ///

115 "Jul-2016" ///

121 "Jan-2017" ///

)xtitle("Calendar Time (months)") ///

legend(order(1 "Mean" 2 "95%CI LB" 3 "95%CI UB")) name("cumdif", replace)

graph export meancumdif.png, width(3000) replace
